# Supplementary material for: A two-part model to estimate inpatient, outpatient, prescribing and care home costs associated with atrial fibrillation in Scotland
Source: BMJ Open. 2020 Mar 18;10(3):e028575. doi: 10.1136/bmjopen-2018-028575 (PMC7150597; doi:10.1136/bmjopen-2018-028575)
Supplement: Supplementary data [file bmjopen-2018-028575supp001.pdf]

ONLINE SUPPLEMENT

Equation I. Probability of healthcare utilisation

$$\Pr(HCE_{it} > 0) = \alpha + \beta_1 \sum_{s=2}^{10} A_{it} + \beta_2 G_i + \beta_3 Y_i + \beta_4 \sum_{s=2}^{5} S_i + \beta_5 \sum_{u=2}^{8} U_i + \beta_6 \sum_{h=2}^{14} H_i$$
$$+ \beta_7 \sum_{c=2}^{3} C_{it} + \beta_8 D_i + \left(\beta_9 \sum_{s=2}^{5} S_i * D_i\right) + \left(\beta_{10} \sum_{c=2}^{3} C_{it} * \sum_{s=2}^{10} A_{it}\right) + u_i$$

Where: A is age at the time of admission (reference category: 0-49 age group); G is sex (reference category: male); Y year of admission; S is SIMD quintile (reference category: most deprived quintile (1)); U is the urban/rural classification (reference category: large urban area);H is health board of inpatient admission (reference category: Greater Glasgow & Clyde); C is the Charlson comorbidity index (reference category: no comorbidities); D is mortality during five year follow-up; u_i is the error term for patient *i* at time *t*.

Equation II. Cost estimation

$$E[HCE] = g(x\beta)$$

Where xβ is the linear predictor for HCE

Equation III. Multiplying first and second part

$$E[HCE|X] = \Pr(HCE > 0|X) * E[HCE|HCE > 0, X]$$

Ciminata G, *et al. BMJ Open* 2020; 10:e028575. doi: 10.1136/bmjopen-2018-028575

Table I. Regression interactions: probability of healthcare resources utilisation and cost estimation

Covariates	Probability (1st modelling part) Coefficient (95%CI)	Std. Err	Probability (2nd modelling part) Coefficient (95%CI)	Std. Err
**Interaction: SIMD - Mortality within 5 years**				
1	Reference			
2	-0.015 (-0.069, 0.038)	0.027	0.056 (0.027, 0.086)	0.015
3	0.046 (-0.007, 0.100)	0.027	0.067 (0.036, 0.097)	0.016
4	0.017 (-0.037, 0.071)	0.028	0.089 (0.059, 0.120)	0.016
5	0.069 (0.013, 0.125)	0.029	0.100 (0.069, 0.132)	0.016
**Interaction: age (year) - Charlson score (1 comorbidity)**				
0-49	Reference			
50-54	-0.195 (-0.342, -0.049)	0.075	-0.016 (-0.124, 0.092)	0.055
55-59	-0.384 (-0.514, -0.255)	0.066	-0.081 (-0.173, 0.012)	0.047
60-64	-0.459 (-0.579, -0.340)	0.061	-0.116 (-0.202, -0.031)	0.044
65-69	-0.500 (-0.613, -0.386)	0.058	-0.161 (-0.244, -0.078)	0.042
70-74	-0.510 (-0.621, -0.399)	0.057	-0.202 (-0.283, -0.121)	0.041
75-79	-0.570 (-0.680, -0.461)	0.056	-0.197 (-0.276, -0.117)	0.041
80-84	-0.594 (-0.704, -0.484)	0.056	-0.209 (-0.290, -0.128)	0.041
85-89	-0.643 (-0.756, -0.531)	0.058	-0.215 (-0.298, -0.132)	0.042
90-max	-0.709 (-0.828, -0.590)	0.061	-0.267 (-0.357, -0.178)	0.045
**Interaction: age (year) - Charlson score (>1 comorbidities)**				
0-49	Reference			
50-54	-0.449 (-0.685, -0.213)	0.121	-0.214 (-0.316, -0.111)	0.052
55-59	-0.539 (-0.751, -0.327)	0.108	-0.209 (-0.305, -0.112)	0.049
60-64	-0.534 (-0.734, -0.334)	0.102	-0.323 (-0.412, -0.234)	0.045
65-69	-0.573 (-0.767, -0.378)	0.099	-0.436 (-0.523, -0.350)	0.044
70-74	-0.650 (-0.842, -0.459)	0.098	-0.520 (-0.604, -0.436)	0.043
75-79	-0.767 (-0.957, -0.577)	0.097	-0.556 (-0.639, -0.473)	0.042
80-84	-0.857 (-1.047, -0.667)	0.097	-0.625 (-0.709, -0.541)	0.043
85-89	-0.967 (-1.159, -0.775)	0.098	-0.661 (-0.747, -0.576)	0.044
90-max	-1.074 (-1.270, -0.878)	0.100	-0.805 (-0.896, -0.714)	0.046

Ciminata G, *et al. BMJ Open* 2020; 10:e028575. doi: 10.1136/bmjopen-2018-028575

Equation IV. Probability of healthcare utilisation (alive at the end of the five-year follow-up period)

$$\Pr(HCE_{it} > 0) = \alpha + \beta_1 \sum_{s=2}^{10} A_{it} + \beta_2 G_i + \beta_3 Y_i + \beta_4 \sum_{s=2}^{5} S_i + \beta_5 \sum_{u=2}^{8} U_i + \beta_6 \sum_{h=2}^{14} H_i + \beta_7 \sum_{c=2}^{3} C_{it} + \left(\beta_8 \sum_{c=2}^{3} C_{it} * \sum_{s=2}^{10} A_{it} \right) + u_i$$

Where: A is age at the time of admission (reference category: 0 -49 age group); G is sex (reference category: male); Y year of admission; S is SIMD quintile (reference category: most deprived quintile (1)); U is the urban/rural classification (reference category: large urban area);H is health board of inpatient admission (reference category: Greater Glasgow & Clyde); C is the Charlson comorbidity index (reference category: no comorbidities); u_i is the error term for patient *i* at time *t*.

Ciminata G, *et al. BMJ Open* 2020; 10:e028575. doi: 10.1136/bmjopen-2018-028575

Table II. Regression interactions: probability of healthcare resources utilisation and cost estimation (alive at the end of the five-year follow-up period)

Covariates	Probability (1st modelling part)		Probability (2nd modelling part)	
	Coefficient (95%CI)	Std. Err	Coefficient (95%CI)	Std. Err
**Interaction: age (year) - Charlson score (1 comorbidity)**				
0-49	Reference			
50-54	-0.190 (-0.341, -0.039)	0.077	-0.015 (-0.126, 0.097)	0.057
55-59	-0.400 (-0.534, -0.266)	0.069	-0.087 (-0.187, 0.014)	0.051
60-64	-0.484 (-0.608, -0.360)	0.063	-0.116 (-0.208, -0.023)	0.047
65-69	-0.531 (-0.649, -0.412)	0.060	-0.143 (-0.233, -0.053)	0.046
70-74	-0.559 (-0.674, -0.443)	0.059	-0.195 (-0.283, -0.107)	0.045
75-79	-0.635 (-0.750, -0.521)	0.058	-0.207 (-0.293, -0.121)	0.044
80-84	-0.680 (-0.796, -0.565)	0.059	-0.215 (-0.304, -0.125)	0.046
85-89	-0.730 (-0.850, -0.610)	0.061	-0.254 (-0.349, -0.159)	0.048
90-max	-0.827 (-0.959, -0.695)	0.067	-0.281 (-0.393, -0.169)	0.057
**Interaction: age (year) - Charlson score (>1 comorbidities)**				
0-49	Reference			
50-54	-0.408 (-0.658, -0.158)	0.127	-0.226 (-0.342, -0.109)	0.059
55-59	-0.504 (-0.726, -0.281)	0.114	-0.235 (-0.348, -0.122)	0.058
60-64	-0.525 (-0.735, -0.316)	0.107	-0.339 (-0.443, -0.236)	0.053
65-69	-0.580 (-0.784, -0.377)	0.104	-0.448 (-0.549, -0.347)	0.051
70-74	-0.713 (-0.913, -0.513)	0.102	-0.565 (-0.663, -0.467)	0.050
75-79	-0.820 (-1.019, -0.621)	0.101	-0.648 (-0.745, -0.552)	0.049
80-84	-0.938 (-1.137, -0.739)	0.102	-0.702 (-0.801, -0.603)	0.051
85-89	-1.074 (-1.276, -0.872)	0.103	-0.769 (-0.873, -0.665)	0.053
90-max	-1.196 (-1.406, -0.986)	0.107	-0.932 (-1.051, -0.814)	0.060

Ciminata G, *et al. BMJ Open* 2020; 10:e028575. doi: 10.1136/bmjopen-2018-028575

Figure I. Average annual costs per patient hospitalised with AF by sector. Cost components with confidence interval are presented for each age group (alive at the end of the five-year follow-up period)

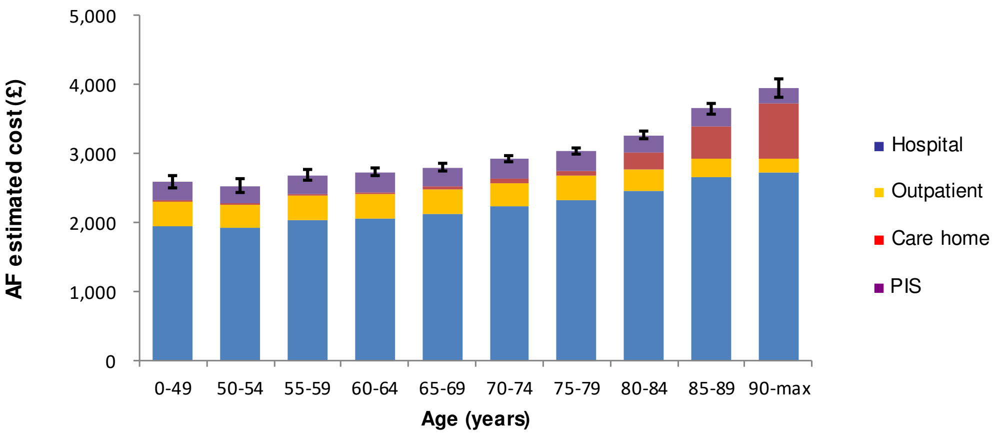

Age groups	Inpatient total cost (£) Mean (95%)	Outpatient total cost (£) Mean (95%)	Care home total cost (£) Mean (95%)	PIS total cost (£) Mean (95%)
(0-49)	1956 (1846, 2066)	360 (344, 375)	17 (16, 49)	267 (243, 291)
(50-54)	1916 (1826, 2007)	344 (332, 356)	14 (8, 37)	259 (242, 275)
(55-59)	2041 (1973, 2110)	351 (342, 360)	17 (1,37)	280 (269, 292)
(60-64)	2071 (2022, 2119)	351 (344, 358)	26 (13, 38)	290 (281, 299)
(65-69)	2135 (2093, 2178)	352 (347, 357)	32 (21, 44)	285 (279, 291)
(70-74)	2230 (2195, 2266)	352 (347, 356)	63 (49, 76)	286 (281, 291)
(75-79)	2329 (2297, 2361)	346 (342, 350)	84 (71, 97)	277 (273, 281)
(80-84)	2457 (2420, 2494)	314 (310, 318)	236 (212, 260)	266 (262, 269)
(85-89)	2651 (2603, 2698)	272 (267, 278)	480 (439, 522)	252 (248, 257)
(90-max)	2734 (2663, 2806)	188 (181, 194)	811 (735, 887)	225 (219, 231)
Total	2253 (2238, 2267)	321 (319, 323)	199 (188, 209)	264 (262, 266)

Ciminata G, *et al. BMJ Open* 2020; 10:e028575. doi: 10.1136/bmjopen-2018-028575

Equation V. Probability of healthcare utilisation (dead at the end of the five-year follow-up period)

$$\Pr(HCE_{it} > 0) = \alpha + \beta_1 \sum_{s=2}^{10} A_{it} + \beta_2 G_i + \beta_3 Y_i + \beta_4 \sum_{s=2}^{5} S_i + \beta_5 \sum_{u=2}^{8} U_i + \beta_6 \sum_{h=2}^{14} H_i + \beta_7 \sum_{c=2}^{3} C_{it} + \left(\beta_8 \sum_{c=2}^{3} C_{it} * \sum_{s=2}^{10} A_{it} \right)$$

Where: A is age at the time of admission (reference category: 0 -49 age group); G is sex (reference category: male); Y year of admission; S is SIMD quintile (reference category: most deprived quintile (1)); U is the urban/rural classification (reference category: large urban area);H is health board of inpatient admission (reference category: Greater Glasgow & Clyde); C is the Charlson comorbidity index (reference category: no comorbidities); u_i is the error term for patient *i* at time *t*.

Note: the model for care home does not include the 0 -49 and 50-54 age groups, as none of those patients incurred any cost related to care home.

Table III. Regression interactions: probability of healthcare resources utilisation and cost estimation (dead at the end of the five-year follow-up period)

Covariates	Probability (1st modelling part) Coefficient (95%CI)	Std. Err	Probability (2nd modelling part) Coefficient (95%CI)	Std. Err
**Interaction: age (year) - Charlson score (1 comorbidity)**				
0-49	Reference			
50-54	-0.057 (-0.456, 0.343)	0.204	0.124 (-0.268, 0.515)	0.200
55-59	0.130 (-0.207, 0.468)	0.172	0.054 (-0.282, 0.389)	0.171
60-64	0.083 (-0.223, 0.389)	0.156	-0.009 (-0.309, 0.291)	0.153
65-69	0.028 (-0.263, 0.320)	0.149	-0.054 (-0.342, 0.234)	0.147
70-74	-0.006 (-0.290, 0.278)	0.145	-0.051 (-0.334, 0.232)	0.144
75-79	-0.078 (-0.358, 0.201)	0.143	0.002 (-0.278, 0.282)	0.143
80-84	-0.200 (-0.478, 0.078)	0.142	-0.048 (-0.326, 0.230)	0.142
85-89	-0.160 (-0.437, 0.117)	0.141	-0.039 (-0.316, 0.239)	0.142
90-max	-0.154 (-0.431, 0.123)	0.141	-0.079 (-0.358, 0.200)	0.142
**Interaction: age (year) - Charlson score (>1 comorbidities)**				
0-49	Reference			
50-54	0.000 (-0.340, 0.339)	0.173	0.066 (-0.268, 0.400)	0.170
55-59	-0.147 (-0.436, 0.142)	0.147	0.041 (-0.240, 0.322)	0.143
60-64	-0.135 (-0.398, 0.128)	0.134	-0.114 (-0.360, 0.132)	0.126
65-69	-0.215 (-0.466, 0.036)	0.128	-0.164 (-0.401, 0.074)	0.121
70-74	-0.192 (-0.436, 0.053)	0.125	-0.237 (-0.470, -0.005)	0.119
75-79	-0.311 (-0.553, -0.070)	0.123	-0.248 (-0.478, -0.018)	0.117
80-84	-0.443 (-0.683, -0.204)	0.122	-0.367 (-0.595, -0.139)	0.116
85-89	-0.394 (-0.633, -0.154)	0.122	-0.394 (-0.623, -0.166)	0.116
90-max	-0.312 (-0.551, -0.073)	0.122	-0.483 (-0.713, -0.254)	0.117

Ciminata G, *et al. BMJ Open* 2020; 10:e028575. doi: 10.1136/bmjopen-2018-028575

Figure II. Average annual costs per patient hospitalised with AF by sector. Cost components with confidence interval are presented for each age group (dead at the end of the five-year follow-up period)

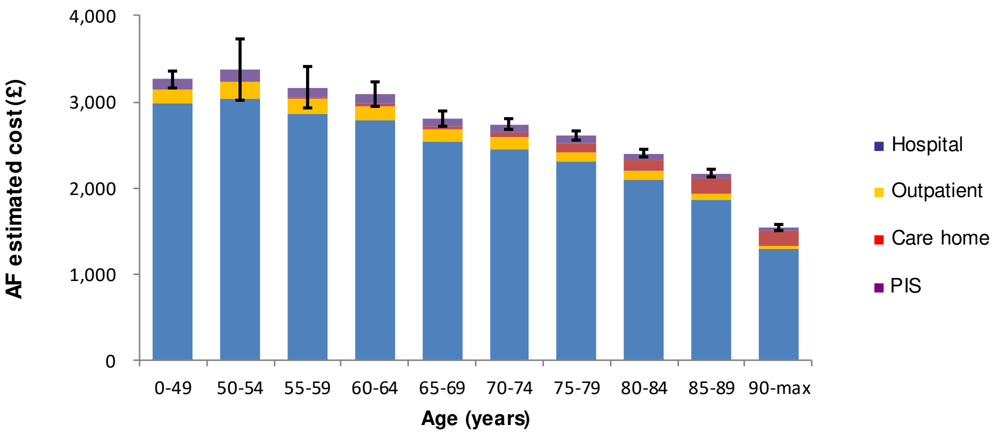

Age groups	Inpatient total cost (£) Mean (95%)	Outpatient total cost (£) Mean (95%)	Care home total cost (£) Mean (95%)	PIS total cost (£) Mean (95%)
(0-49)	2976 (2603, 3349)	161 (136, 186)	n/a	125 (65, 185)
(50-54)	3037 (2712, 3362)	203 (177, 228)	n/a	130 (97, 164)
(55-59)	2865 (2649, 3080)	181 (165, 197)	9 (5, 23)	115 (95, 135)
(60-64)	2795 (2661, 2929)	162 (153, 170)	28 (3, 53)	111 (99, 122)
(65-69)	2536 (2450, 2621)	154 (147, 160)	26 (12, 39)	95 (88, 102)
(70-74)	2448 (2386, 2510)	138 (134, 142)	60 (42, 78)	97 (91, 102)
(75-79)	2302 (2257, 2347)	118 (115, 121)	100 (80, 120)	83 (80, 86)
(80-84)	2099 (2062, 2136)	93 (91, 95)	139 (121, 157)	70 (67, 72)
(85-89)	1859 (1826, 1892)	69 (67, 71)	187 (167, 206)	58 (56, 60)
(90-max)	1289 (1263, 1316)	33 (31, 34)	180 (162, 199)	35 (34, 36)
Total	2018 (2001, 2034)	91 (90, 93)	142 (132, 152)	66 (65, 67)

Note: the care home total cost estimation does not include the 0 -49 and 50-54 age groups, as none of those patients incurred any cost related to care home.

Ciminata G, *et al. BMJ Open* 2020; 10:e028575. doi: 10.1136/bmjopen-2018-028575
