# Supplementary material for: A two-part model to estimate inpatient, outpatient, prescribing and care home costs associated with atrial fibrillation in Scotland
Source: BMJ Open. 2020 Mar 18;10(3):e028575. doi: 10.1136/bmjopen-2018-028575 (PMC7150597; doi:10.1136/bmjopen-2018-028575)
Supplement: Author's manuscript [file bmjopen-2018-028575.draft_revisions.pdf]

BMJ Open is committed to open peer review. As part of this commitment we make the peer review history of every article we publish publicly available.

When an article is published we post the peer reviewers' comments and the authors' responses online. We also post the versions of the paper that were used during peer review. These are the versions that the peer review comments apply to.

The versions of the paper that follow are the versions that were submitted during the peer review process. They are not the versions of record or the final published versions. They should not be cited or distributed as the published version of this manuscript.

BMJ Open is an open access journal and the full, final, typeset and author-corrected version of record of the manuscript is available on our site with no access controls, subscription charges or pay-per-view fees (http://bmjopen.bmj.com).

If you have any questions on BMJ Open's open peer review process please email info.bmjopen@bmj.com

Inpatient, Outpatient, Prescribing and Care Home Costs associated with Atrial Fibrillation

Journal:	*BMJ Open*
Manuscript ID	bmjopen-2018-028575
Article Type:	Research
Date Submitted by the Author:	14-Dec-2018

Complete List of Authors:	Ciminata, Giorgio; University of Glasgow Institute of Health and Wellbeing, Health Economics and Health Technology Assessment (HEHTA)<br>Geue, Claudia; University of Glasgow, Institute of Health and Wellbeing<br>Langhorne, Peter; University of Glasgow, Institute of Cardiovascular and Medical Sciences<br>Wu, O; University of Glasgow

Keywords:	HEALTH ECONOMICS, Atrial Fibrillation, Stroke < NEUROLOGY, Cost Analysis

SCHOLARONE™
Manuscripts

I, the Submitting Author has the right to grant and does grant on behalf of all authors of the Work (as defined in the below author licence), an exclusive licence and/or a non-exclusive licence for contributions from authors who are: i) UK Crown employees; ii) where BMJ has agreed a CC-BY licence shall apply, and/or iii) in accordance with the terms applicable for US Federal Government officers or employees acting as part of their official duties; on a worldwide, perpetual, irrevocable, royalty-free basis to BMJ Publishing Group Ltd ("BMJ") its licensees and where the relevant Journal is co-owned by BMJ to the co-owners of the Journal, to publish the Work in this journal and any other BMJ products and to exploit all rights, as set out in our licence.

The Submitting Author accepts and understands that any supply made under these terms is made by BMJ to the Submitting Author unless you are acting as an employee on behalf of your employer or a postgraduate student of an affiliated institution which is paying any applicable article publishing charge ("APC") for Open Access articles. Where the Submitting Author wishes to make the Work available on an Open Access basis (and intends to pay the relevant APC), the terms of reuse of such Open Access shall be governed by a Creative Commons licence – details of these licences and which Creative Commons licence will apply to this Work are set out in our licence referred to above.

Other than as permitted in any relevant BMJ Author's Self Archiving Policies, I confirm this Work has not been accepted for publication elsewhere, is not being considered for publication elsewhere and does not duplicate material already published. I confirm all authors consent to publication of this Work and authorise the granting of this licence.

For peer review only

Inpatient, Outpatient, Prescribing and Care Home Costs associated with Atrial Fibrillation

Authors: Ciminata G (Research Assistant)[1], Geue C (Lecturer in Health Economics)[1], Langhorne P (Professor of Stroke Care)[2], Wu O (Professor of Health Technology Assessment)[1].

1. Health Economics and Health Technology Assessment (HEHTA), Institute of Health & Wellbeing, University of Glasgow, Glasgow G12 8RZ, UK
2. Institute of Cardiovascular & Medical Sciences, University of Glasgow, Glasgow G31 2ER, UK

Corresponding author:
Mr Giorgio Ciminata (MSc), g.ciminata.1@research.gla.ac.uk

Cover title: Inpatient, Outpatient, Prescribing and Care Home Costs associated with Atrial Fibrillation

Total number of tables and figures: Figures: 2, Tables: 3

Subject terms: Atrial Fibrillation

Key words: Atrial Fibrillation, Stroke, cost analysis

Word count: 3296

For peer review only

Abstract

Objective: To estimate inpatient, outpatient, prescribing and care-home costs associated with atrial fibrillation (AF), using population-based, individual-level linked data.

Design: A two-part model was employed to estimate the probability of resource utilisation and costs conditional on positive utilisation using individual-level linked data.

Settings: Scotland, five years following first hospitalisation for AF between 1997 and 2015.

Participants: Patients aged ≥50 years, hospitalised with a known diagnosis of AF or atrial flutter.

Primary and secondary outcome measures: Inpatient, outpatient, prescribing and care home costs.

Results: The mean annual cost associated with an AF patient was estimated at £3861 (95% CI £3842-£3880). Inpatient admissions and outpatient visits accounted for 77% and 5% of total costs, respectively; prescriptions and care home stay accounted for 4% and 14% of total costs. Inpatient costs was the main driver across all age groups. While inpatient cost contributions (~80%) were constant between 50 and 84 years, they decreased for patients over 85 years. This is offset by increasing care-home cost contributions. Mean annual costs associated with AF increased significantly with increasing number of comorbidities.

Conclusion: This study used a contemporary and representative cohort, and a comprehensive approach to estimate costs associated with AF, taking into account resource utilisation beyond hospital care. While overall costs, considerably affected by comorbidity, did not increase with increasing age, care-home costs increased proportionally with age. Inpatient admission was the main contributor to the overall financial burden of AF, highlighting the need for improved mechanisms of early diagnosis to prevent hospitalisations.

For peer review only

Article summary

Strengths and limitations of this study

- Costs are estimated through an incidence-based approach using patient-level morbidity records.
- Sufficient follow-up time is used to capture all relevant costs to generate a contemporary estimate of health and care home costs related to AF.
- Scotland offers a robust record linkage system, where administrative patient-level health data are routinely collected.
- Data on primary care consultations were not available for linkage at a national level, however the impact this might have on overall costs is expected to be small.
- The potential risk of AF going undiagnosed and clinical miscoding of morbidity records may lead to an underestimation of the AF cohort and associated costs.
- Other limitations are those inherent to the nature of administrative data, such as miscoding or incomplete records.

For peer review only

Introduction

Atrial Fibrillation (AF) is the most common form of arrhythmia. In Scotland AF affects 1.8% of the adult population, and rises to 6% among those aged 65 years or over [1]. In an ageing population, AF has a substantial impact on the economic burden of the healthcare system.

A number of cost analyses on estimating the economic burden of AF exist. The majority of these studies are of selective cohorts of AF patients, based on data sourced from administrative database [2-4], health insurance databases [2, 5-7], hospital records [8, 9] and surveys [10]. Direct medical costs related to inpatient admissions, outpatient visits, as well as prescriptions have been included in these estimates; [2-10] indirect costs related to loss of productivity have been estimated among patients who were at working ages [6, 7].

Many of these studies included relatively young patients – those aged 18-20 years or older [2, 4-6, 8-10], or those under the age of 65 years [7]. However, the prevalence of AF increases significantly with age, and is most affected by patients who are older than 50 years; in patients under the age of 50, AF is often associated with structural heart disease, hyperthyroidism, or alcohol excess [11]. Hence, inclusion of younger patients and the exclusion of older patients may result in imprecise cost estimates.

There is a lack of generalisable studies based on large national population datasets that examine the total and the distribution of costs associated with AF [12]. The aim of this study was to quantify the inpatient, outpatient, prescribing and care home costs associated with AF over a five-year period. Using record-linkage of national datasets from Scotland, we also examined the distribution of costs that are attributable to AF.

For peer review only

Methods

Cost analyses or cost of illness studies typically adopt either the prevalence or incidence based approaches [13]. In the context of AF, the prevalence based approach determines costs attributable to all cases of AF in a given year, while the incidence based approach determines costs of new cases of AF in a given time period. In the present study, costs were estimated with an incidence-based approach.

For peer review only

Data

Data were obtained from the Information Services Division (ISD) of NHS Scotland as part of a wider project that used routinely collected data to evaluate clinical effectiveness and cost-effectiveness of Direct Oral Anticoagulants (DOACs) in the prevention of stroke in the AF population. Inpatient records for patients with a diagnosis of AF or atrial flutter between 1997 and 2015 were extracted from the General Acute Inpatient and Day Case Scottish Morbidity Records 01 (SMR01). These records contain all general acute admissions, categorized as inpatients or day cases, discharged from non-obstetric and non-psychiatric specialties [14]. Incident AF events (ICD10 code I48) were identified using all six diagnostic positions in SMR01, with a look back period of five years to minimise double counting. After checking for data entry errors and removal of duplicate records, an initial AF cohort consisting of 279,883 individuals hospitalised with a diagnosis of AF or atrial flutter was identified. For the purpose of this analysis, only patients aged 50 years or older were included. Based on clinical advice and the evidence that prevalence and incidence of AF typically increase exponentially from 50 years onwards [11], the analysis including 50 years age group at the lowest range would be inclusive of all patients potentially at risk of AF. The final dataset for analysis consisted of 272,716 patients.

Individual-level data linkage was then carried out with outpatient clinic attendance (Outpatient Attendance Scottish Morbidity Records 00; SMR00), the prescribing information system (PIS), care home census and mortality records (National Records for Scotland, NRS). Records from SMR00 include information on new and follow up outpatient appointments for any clinical specialty [15]. The PIS database includes prescribing records for all medicines and their associated costs, which are prescribed and dispensed by community pharmacies, dispensing doctors and a small number of specialist appliance suppliers [16]. The quality of PIS data is guaranteed by an electronic data capture, and it passes several stages of quality control before and after data are submitted [17]. The care home census combines the former Residential Care Home Census (run by the Scottish Government) and the Private Nursing Homes Census (run by ISD Scotland). Items reported in the care home census include discharge dates to care home residency such as NHS and private nursing homes, as well as an indication on whether nursing care is required [16].

Patients were followed up for five years following incident AF event in terms of their healthcare resource use, care home admissions and mortality. Since AF is often a precursor of stroke and cardiovascular conditions, an estimation of costs for a period of five years post AF event would allow us to fully capture costs associated with an AF patient.

Costing

Inpatient care costs were obtained from the latest (2013/2014) Scottish National Tariff (SNT), a list of standard average prices based on Healthcare Resource Groups (HRGs) [17, 18]. The SNT uses HRG4 for grouping clinically similar treatments that use similar levels of healthcare resources. After defining a total cost per episode, the total cost for a continuous inpatient stay (CIS) was calculated.

For peer review only

A CIS describes the entire duration of an inpatient stay from the date of admission to the date of discharge and can consist of several episodes in different specialties. Since the SNT is based on spells of care (inpatient stay within the same specialty) rather than individual inpatient episodes or a CIS, a CIS was partitioned into spells when a change in specialty occurred [17]. If within a CIS, two or more episodes were in the same specialty, only the highest incurred cost was taken into account, and the remaining episodes were replaced with a zero cost. Outpatient costs were obtained by assigning outpatient specialty costs, to outpatient attendances [17]. Unit costs were specific to whether the outpatient attendance took place at a consultant or nurse led clinic [15].

The cost of each prescription dispensed per patient was obtained from PIS [19]. Firstly, the price per unit was obtained by dividing the item price by the pack size. Secondly, the total number of items dispensed was obtained by multiplying the number of items dispensed by the number of instalments. Care home costs, obtained from the care home census, were based on length of stay or residency. Care home residency was established from care home census records, reporting admission to a care home like structure [16]. An average of care home charges for long stay residents was calculated using information on whether nursing care was provided or not. The average weekly care home charge was expressed per day, so that only the effective days spent in a care home were costed.

Econometric model

Healthcare expenditure data are typically characterised by: i) a significant proportion of zero-cost observations for individuals who have not utilised any healthcare resources in a given time period, and ii) akewed distribution for positive costs. A two-part model was used [20, 21].

For peer review only

In the first part of the model, the probability of using a healthcare service in a given time period was estimated using a probit model (Equation I, please see online supplement). The same explanatory variables were used in the second part of the model, with a gamma distribution and log link, estimating costs conditional on having incurred positive costs (Equation II, please see online supplement). Mean costs per patient per year following their incident AF event were calculated by multiplying first and second modelling part (Equation III, please see online supplement).

In order to account for the skewed nature of cost data, generalised linear models (GLMs) were used. These were compared against ordinary least squares regression (OLS) and log transformed OLS by means of the Akaike Information Criterion (AIC), which measures goodness of fit. When comparing the different models, GLM reported the lowest AIC indicating the best fit for the given set of data. A user-written STATA programme "glmdiagnostic.do" [20], performing four different tests simultaneously, was used to identify the most appropriate distributional family and link function.

Econometric model covariates

The two-part model adjusted for age, sex, year of inpatient admission, socio-economic status, urban-rural classification, health board, comorbidities and mortality. These covariates are considered to be the main confounders that have an effect on costs incurred by an AF population. We controlled for age because AF and associated comorbidities are age-related conditions, and may have an impact on the overall costs. We also assumed costs to vary between males and females, in particular those for care home residency. Variation in healthcare utilisation and associated costs and care home residency by socio-economic status is controlled for using the Scottish Index of Multiple Deprivation (SIMD).

For peer review only

The SIMD reflects areas of multiple deprivation ranked from the most to the least deprived and expressed as quintiles where the most and the least deprived areas are represented by 1 and 5 respectively [22]. In Scotland, there are 14 regional health boards responsible for the provision of healthcare [23]. Hence, potential differences in healthcare utilisation and prescribing costs, may reflect variation in clinical practice and prescribing behaviour rather than the ability of patients to access care. Patients living in urban areas may have easier access to care compared to patients living in more remote areas, which is controlled for including the 8-fold classification measuring rurality [24].

Patients with one or more comorbidities are expected to incur significantly higher costs than those with none. We accounted for this by including the Charlson comorbidity index, where 1 indicates the absence of comorbidities, 2 the presence of only a single comorbidity and 3 the presence of more than one comorbidity [25]. Two interaction terms between age and comorbidities, and mortality and SIMD were included in the econometric model. Intuitively, a relationship of direct proportionality between age and comorbidities suggests that the level of comorbidities increases, as patients get older. Similarly, the socio-economic status may significantly influence the rate of socio-economic inequalities in mortality [26].

Ethics statement

The authors state that no ethical approval was needed.

For peer review only

Results

Cohort characteristics

Of the 272,716 AF patients with a mean age of 71 years (SD 10.6), the majority were identified in the two largest urban health board areas (Greater Glasgow & Clyde and Lothian), accounting for 22.3% and 14.9% respectively. This is also reflected in our categorisation of geographical areas, where large urban represented 38.6% and other urban areas represented 29.8% of the total AF cohort. Greater proportion of patients live in areas belonging to the most deprived quintile compared with those living in the least deprived areas – SIMD quintile 1 and quintile 5 representing 22.6% and 16.4% of the AF cohort respectively (Table1).

Econometric modelling results

Regression results for both modelling parts are presented in Table 2. Overall, an inversely U-shaped association between age and the likelihood of utilising any health or social care services was observed – a gradual increment in the likelihood in resource use with advancing age up to 80 years, when compared with the reference group (50-54 years), while patients 80 years or older showing a decreased probability of utilising healthcare services. However, this association was not observed in the second modelling part model, estimating costs conditional on having incurred positive costs, where a statistically significant gradient between age and costs indicated increasing costs as the cohort ages. The use of health or social care services and associated costs also increased significantly for patients living in the most deprived areas, when compared with patients living in areas with the lowest level of deprivation. The effect of socio-economic status on healthcare utilisation was also measured for those who are alive at the end of the five-year follow-up period through an interaction term between SIMD and mortality, but no statistically significant effect was found.

For peer review only

Full details of regression results for interaction terms are presented in the supplementary online material (Table I, please see online supplement).

For patients with comorbidities, the probabilities of utilising healthcare services were 49.5% (one comorbidity) and 78.6% (two or more comorbidities) greater than the probability for those with no comorbidities. Although healthcare utilisation increased with the number of comorbidities, the interaction term between age and comorbidities indicated that as patients get older the use of healthcare services on average is lower for patients with one or more comorbidities than those with none. The decrease in healthcare utilisation by age is more pronounced in patients with more comorbidities than in those with only one comorbidity. The difference in healthcare costs between comorbidity categories indicated that in the presence of one or more comorbidities, on average healthcare costs decrease as patients get older.

Cost estimates

The estimated mean annual cost per AF patient was £3861 (95% CI: £3842-£3880). The estimated total costs and distribution of costs according to sex are shown in Table 3. While there is little difference between the total costs and the distribution of costs for inpatient, outpatient and prescription costs, the difference seems more pronounced when comparing the care home component of costs (5% of total costs among males vs 7% of total costs among females). The average annual cost per AF patient by age and for each health or care home sector is shown in Figure 1.

For peer review only

Considering the individual contribution of each cost component to the overall costs, inpatient costs was the main driver across all age groups. While inpatient cost contribution remained constant with an average contribution of about 80% to the overall cost for patients aged between 50 and 84 years, it decreased for patients over 85 years of age. Similar patterns was observed for outpatient and prescribing costs. On the contrary, the contribution of care home costs to the overall costs increased with age (0.5% for patients aged 50-54 years and approximately 11% for patients who are 90 years or older). The contribution of each setting to the total health and care home costs by the number of existing comorbidities is illustrated in Figure 2. While inpatient and total costs vary considerably with the number of comorbidities, outpatient and care home contributions remain fairly constant.

Discussion

A greater proportion of AF patients were found in areas with the highest index of deprivation. This, combined with the likelihood for people living in the most deprived quintile having longer inpatient stays due to a lack of support at home, may explain the difference in inpatient care utilisation between patients from the most and the least deprived areas, with associated costs being higher for the former group. As AF is more likely to affect the elderly, AF related costs were expected to increase with age. As health deteriorates with age, older age groups are assumed to make greater use of healthcare services, and therefore incur higher costs than younger age groups. However, age was found to have a modest impact on overall healthcare costs, being fairly consistent across age groups. This finding is in line with existing evidence indicating that healthcare expenditure depends not only on patients' calendar age, but is also significantly associated with remaining lifetime [27].

For peer review only

Any observed correlation between healthcare expenditure and age may therefore be attributable to the fact that the proportion of patients who are at the end of their lives is substantially greater in older rather than younger age groups [27]. On the other hand, comorbidity had a considerable effect on the overall cost, increasing significantly in patients with more than one comorbidity. However, the decrease in healthcare utilisation by age is more pronounced in patients with more comorbidities than in those with only one comorbidity. Decreasing inpatient and outpatient costs for the oldest patients were offset by increasing care home costs, in particular for women. Indeed, the main cause for higher overall costs incurred by women is attributable to the higher likelihood for elderly women to reside in care homes. Interestingly, care home contribution to the overall costs was noticeably lower for patients with multiple comorbidities than for those with none or one comorbidity. This may suggest that sicker patients are more likely to be in hospital than in a care home.

To date, only one single study published in 2004 has estimated the cost of AF in Scotland; the authors estimated the cost of AF in 1995/1996, and projected these to the year 2000 [28]. Previous work has focussed on a 12-months follow-up, which seems limited in order to capture all healthcare resource utilisation for AF patients. Our study offers a longer follow-up and a contemporary estimate of healthcare costs related to AF including all relevant care settings. Our study offers a distinct advantage over previous work as costs, rather than being based on extrapolated rates using a prevalence-based approach [28], are estimated with an incidence-based method using patient-level morbidity records. Using an incidence based approach to costing and a broad perspective to capture the majority of costs associated with AF, several routinely collected administrative datasets from Scotland were combined, including care home utilisation.

For peer review only

Existing studies, including ours, regardless of econometric model choice and covariates used, show that costs due to inpatient admission are the main contributor to overall AF related healthcare cost. This is a pertinent finding and highlights strategies to improve diagnosis of AF. In Scotland, 1 in 3 patients with AF are currently undiagnosed and hence do not receive treatment that could prevent their AF to progress and potentially require inpatient admission [29]. The European AF management guidelines and the Scottish Cross-Party Group 'Heart Disease and Stroke', recently recommended that people who are 65 years or older and at risk of AF and associated comorbidities such as cardiovascular disease, diabetes or respiratory disease should be screened opportunistically in primary care, pharmacies or community settings [29, 30]. With rigorous screening and appropriate treatment, hospitalisations could be avoided and costs reduced.

Although we have captured most healthcare sectors and related costs, we were not able to obtain national data on primary care consultations, as these data are currently not routinely available for linkage in Scotland. However, the costs associated with primary care consultations is expected to have a limited impact on the overall total AF related costs. Further, there is potential risk of AF going undiagnosed and clinical miscoding of morbidity records, leading to an underestimation of the AF cohort and associated costs. Nevertheless, by using a cohort of patients hospitalised with AF we were able to capture more severe cases of AF. Prescribing and care home data were only available respectively from 2009 to 2012, their contribution to overall AF related costs might also be underestimated. Other limitations are inherent to the nature of administrative data, such as missing records or incomplete data. Recognising these limitations, we were nevertheless able to harness high quality patient-level linked data to identify a cohort of AF patients and to estimate AF related costs in Scotland.

For peer review only

The inclusion of all available cost components is crucial for establishing overall costs, as these often extend beyond hospitalisation. The study identifies hospitalisation as the main cost driver and suggests that the implementation of AF screening policies could substantially reduce AF related health care costs. Most importantly, the study concludes that patient's age has a limited impact on the overall AF related cost, and therefore may contribute much less to future growth of AF related cost in an ever-ageing Scottish population . Future work will be able to utilise Scottish Stroke Care Audit (SSCA) records, allowing for the identification of additional AF patients; these are patients hospitalised with a stroke, where AF has been recorded in audit data as an underlying comorbidity. Being able to complement inpatient records with SSCA records will allow us to capture more AF patients in Scotland. Moreover, future research may be able to include indirect costs associated with productivity-loss by linking morbidity and prescribing data to national data from the Department for Work and Pensions, for instance.

For peer review only

Acknowledgements: We thank the members of the Information Services Division, National Services Scotland, for their support. We acknowledge the support from The Farr Institute @ Scotland. The Farr Institute @ Scotland is supported by a 10-funder consortium: Arthritis Research UK, the British Heart Foundation, Cancer Research UK, the Economic and Social Research Council, the Engineering and Physical Sciences Research Council, the Medical Research Council, the National Institute of Health Research, the National Institute for Social Care and Health Research (Welsh Assembly Government), the Chief Scientist Office (Scottish Government Health Directorates), the Wellcome Trust (MRC Grant No: MR/K007017/1).

Author contributions: All authors conceived the article. GC carried out the statistical analysis and prepared the first draft of the manuscript. All authors contributed to editing the manuscript and approved the final version submitted for publication.

Funding sources: This work was supported by Farr Institute for Health Informatics Research, Scotland, MRC, grant number MR/K007017/1

Competing interest: The authors declare no conflict of interest.

Data Statement: All data underlying the analyses are confidential and subject to disclosure control. Data can only be obtained through application to ISD via the Public Benefit and Privacy Panel (PBPP).

For review only

References

1. Scotland, H.I. *Prevention of stroke in patients with atrial fibrillation*. 2013 [cited 2016 January 2016]; Available from: http://www.sign.ac.uk/assets/af_publication.pdf.
2. Bennell, M.C., et al., *Identifying predictors of cumulative healthcare costs in incident atrial fibrillation: a population-based study.* J Am Heart Assoc, 2015. **4**(4).
3. Hallinen, T., et al., *Direct costs of warfarin treatment among patients with atrial fibrillation in a Finnish health care setting.* Curr Med Res Opin, 2006. **22**(4): p. 683-92.
4. Nelson, W.W., et al., *Out-of-range international normalized ratio values and healthcare cost among new warfarin patients with non-valvular atrial fibrillation.* J Med Econ, 2015. **18**(5): p. 333-40.
5. Ghate, S.R., et al., *All-cause and bleeding-related health care costs in warfarin-treated patients with atrial fibrillation.* J Manag Care Pharm, 2011. **17**(9): p. 672-84.
6. Rohrbacker, N.J., et al., *The burden of atrial fibrillation and other cardiac arrhythmias in an employed population: associated costs, absences, and objective productivity loss.* J Occup Environ Med, 2010. **52**(4): p. 383-91.
7. Wu, E.Q., et al., *Economic burden and co-morbidities of atrial fibrillation in a privately insured population.* Curr Med Res Opin, 2005. **21**(10): p. 1693-9.
8. Holstenson, E., et al., *Predictors of costs related to cardiovascular disease among patients with atrial fibrillation in five European countries.* Europace, 2011. **13**(1): p. 23-30.
9. Reynolds, M.R., et al., *Healthcare resource utilization and costs associated with recurrent episodes of atrial fibrillation: the FRACTAL registry.* J Cardiovasc Electrophysiol, 2007. **18**(6): p. 628-33.
10. Jonsson, L., et al., *Cost of illness and drivers of cost in atrial fibrillation in Sweden and Germany.* Appl Health Econ Health Policy, 2010. **8**(5): p. 317-25.
11. Sankaranarayanan, R., et al., *Comparison of Atrial Fibrillation in the Young versus That in the Elderly: A Review.* Cardiology Research and Practice, 2013. **2013**: p. 16.
12. Johnsen, S.P., et al., *Cost of illness of atrial fibrillation: a nationwide study of societal impact.* BMC Health Services Research, 2017. **17**(1): p. 714.
13. Tarricone, R., *Cost-of-illness analysis. What room in health economics?* Health Policy, 2006. **77**(1): p. 51-63.
14. Scotland, I.S.D. *General Acute Inpatient and Day Case - Scottish Morbidity Record (SMR01)*. 2015 [cited 2017; Available from: http://www.ndc.scot.nhs.uk/National-Datasets/data.asp?SubID=2.
15. Scotland, I.S.D. *Outpatient Attendance (SMR00)*. 2015 [cited 2015 January 2015]; Available from: http://www.ndc.scot.nhs.uk/National-Datasets/data.asp?SubID=2.
16. Scotland, I.S.D. *Care Home Census 2015*. 2015 November 2015]; Available from: http://www.isdscotland.org/Health-Topics/Health-and-Social-Community-Care/Care-Homes/Previous-Publications/.
17. Scotland, I.S.D. *Scottish National Tariff*. 2015 [cited 2015 December 2015]; Available from: http://www.isdscotland.org/Health-Topics/Finance/Scottish-National-Tariff/.
18. Groups, H.R. *Healthcare Resource Groups*. 2015 November 2015]; Available from: http://www.hscic.gov.uk/hrg.
19. Alvarez-Madrazo, S., et al., *Data Resource Profile: The Scottish National Prescribing Information System (PIS).* International Journal of Epidemiology, 2016.
20. Henry, A.G., et al., *Economic Evaluation in Clinical Trials*. 'Oxford University Press': Oxford, UK.
21. Manning, W.G., A. Basu, and J. Mullahy, *Generalized modeling approaches to risk adjustment of skewed outcomes data.* Journal of Health Economics, 2005. **24**(3): p. 465-488.

For peer review only

22. Government, T.S. *Scottish Index of Multiple Deprivation*. 2015 [cited 2015 November 2015]; Available from: http://www.gov.scot/Topics/Statistics/SIMD.
23. Government, T.S. *NHS Boards*. 2015 [cited 2015 January 2015]; Available from: http://www.gov.scot/Topics/Health/NHS-Workforce/NHS-Boards.
24. Government, T.S. *Scottish Government Urban Rural Classification*. 2015 [cited 2015 January 2015]; Available from: http://www.gov.scot/Topics/Statistics/About/Methodology/UrbanRuralClassification.
25. Charlson, M.E., et al., *A new method of classifying prognostic comorbidity in longitudinal studies: Development and validation.* Journal of Chronic Diseases, 1987. **40**(5): p. 373-383.
26. Leyland, A.H., et al., *Cause-specific inequalities in mortality in Scotland: two decades of change. A population-based study.* BMC Public Health, 2007. **7**: p. 172.
27. Zweifel, P., S. Felder, and M. Meiers, *Ageing of population and health care expenditure: a red herring?* Health Econ, 1999. **8**(6): p. 485-96.
28. Stewart, S., et al., *Cost of an emerging epidemic: an economic analysis of atrial fibrillation in the UK.* Heart, 2004. **90**(3): p. 286-292.
29. Stroke, C.-P.G.o.H.D.a. *A Focus on Atrial Fibrillation in Scotland 2017*. 2017 March 2018]; Available from: https://www.chss.org.uk/documents/2018/01/a-focus-on-atrial-fibrillation-in-scotland-pdf.pdf.
30. Camm, A.J., et al., *2012 focused update of the ESC Guidelines for the management of atrial fibrillation: an update of the 2010 ESC Guidelines for the management of atrial fibrillation. Developed with the special contribution of the European Heart Rhythm Association.* Eur Heart J, 2012. **33**(21): p. 2719-47.

For peer review only

Figure legends

Figure 1. Average annual costs per patient hospitalised with AF by sector. Cost components with confidence interval are presented for each age group.

Figure 2. Average cost per patient hospitalised with AF by Charlson Comorbidity Index. Cost components with confidence interval are presented for each Comorbidity category.

For peer review only

Tables

Table 1. Baseline characteristics of AF patients 50 years or older

Characteristics	N (%)
Number of patients	272,716
Mean age at first admission*(SD)**(range)	71 *(10.6) **(50 -108)
Sex	
Male	135,683 (49.8)
Female	137,033 (50.2)
Health Boards	
Greater Glasgow & Clyde	60,774 (22.3)
Lothian	40,498 (14.9)
Lanarkshire	30,105 (11.0)
Grampian	25,208 (9.2)
Ayrshire & Arran	24,468 (7.9)
Tayside	21,543 (9.0)
Fife	18,584 (6.8)
Highland	17,612 (6.5)
Forth valley	13,308 (4.9)
Dumfries & Galloway	9,645 (3.5)
Borders	7,148 (2.6)
Western isles	1,812 (0.7)
Shetland	1,009 (0.4)
Orkney	1,002 (0.4)
Geography	
Large/urban	104,841 (38.4)
Other/urban	80,794 (29.6)
Accessible small towns	24,492 (9.0)
Remote small towns	8,126 (3.0)
Very remote small towns	3,712 (1.4)
Accessible rural	30,122 (11.1)
Remote rural	10,277 (3.8)
Very remote rural	9,908 (3.6)
SIMD quintile	
1	61,686 (22.6)
2	61,704 (22.6)
3	54,937 (20.1)
4	49,448 (18.1)
5	44,933 (16.4)
Comorbidity	
no comorbidity	36,345 (13.4)
1 comorbidity	52,159 (19.2)
>1 comorbidities	182,827 (67.4)

Table 2. Regression results: probability of healthcare resources utilisation and cost estimation

Covariates	Probability (1st modelling part)		Cost Ratios (2nd modelling part)	
	Coefficient (95% CI)	Std. Err	Coefficient (95% CI)	Std. Err
Age group (years)				
50-54	Reference			
55-59	0.036 (-0.034, 0.107)	0.036	0.045 (-0.005, 0.096)	0.026
60-64	0.113 (0.045, 0.180)	0.034	0.088 (0.040, 0.136)	0.025
65-69	0.134 (0.069, 0.199)	0.033	0.120 (0.073, 0.167)	0.024
70-74	0.126 (0.062, 0.191)	0.033	0.176 (0.130, 0.221)	0.023
75-79	0.182 (0.118, 0.247)	0.033	0.183 (0.138, 0.228)	0.023
80-84	0.075 (0.009, 0.141)	0.034	0.246 (0.200, 0.292)	0.023
85-89	0.022 (-0.048, 0.091)	0.035	0.334 (0.286, 0.382)	0.024
90-max	-0.208 (-0.283, -0.132)	0.039	0.474 (0.420, 0.527)	0.027
Sex				
Male	Reference			
Female	0.029 (0.012, 0.047)	0.009	0.052 (0.042, 0.062)	0.005
Date of admission	0.173 (0.172, 0.175)	0.001	-0.023 (-0.024, -0.022)	0.001
SIMD quintile				
1	Reference			
2	0.036 (-0.012, 0.083)	0.024	-0.054 (-0.079, -0.028)	0.013
3	-0.028 (-0.075, 0.019)	0.024	-0.075 (-0.102, -0.049)	0.014
4	-0.031 (-0.078, 0.017)	0.024	-0.106 (-0.132, -0.080)	0.013
5	-0.042 (-0.090, 0.006)	0.024	-0.139 (-0.165, -0.113)	0.013
Geography				
Large urban	Reference			
Other urban	-0.144 (-0.171, -0.118)	0.013	-0.024 (-0.039, -0.010)	0.007
Accessible small towns	-0.163 (-0.197, -0.128)	0.018	-0.040 (-0.059, -0.021)	0.010
Accessible rural	-0.213 (-0.247, -0.180)	0.017	-0.044 (-0.063, -0.025)	0.010
Remote small towns	-0.160 (-0.213, -0.106)	0.027	0.002 (-0.030, 0.034)	0.016
Remote rural	-0.298 (-0.347, -0.250)	0.025	-0.038 (-0.067, -0.009)	0.015
Very remote small towns	-0.398 (-0.480, -0.315)	0.042	-0.053 (-0.105, -0.002)	0.026
Very remote rural	-0.371 (-0.434, -0.308)	0.032	-0.059 (-0.100, -0.018)	0.021
Health boards				
Great Glasgow and Clyde	Reference			
Lothian	-0.041 (-0.073, -0.010)	0.016	-0.032 (-0.049, -0.016)	0.008
Lanarkshire	0.003 (-0.031, 0.038)	0.018	-0.065 (-0.084, -0.047)	0.009
Ayrshire and Arran	-0.356 (-0.393, -0.318)	0.019	-0.044 (-0.066, -0.022)	0.011
Grampian	0.020 (-0.018, 0.058)	0.019	-0.059 (-0.079, -0.039)	0.010
Tayside	-0.401 (-0.436, -0.365)	0.018	-0.084 (-0.104, -0.063)	0.010
Fife	-0.055 (-0.099, -0.012)	0.022	-0.007 (-0.031, 0.018)	0.012
Highland	-0.156 (-0.209, -0.104)	0.027	-0.047 (-0.078, -0.016)	0.016
Forth Valley	-0.473 (-0.516, -0.431)	0.022	-0.113 (-0.139, -0.087)	0.013

Dumfries and Galloway	-0.303 (-0.353, -0.252)	0.026	-0.137 (-0.167, -0.106)	0.015
Borders	-0.487 (-0.541, -0.434)	0.027	-0.090 (-0.124, -0.056)	0.017
Western Isles	-1.075 (-1.176, -0.974)	0.052	0.473 (0.396, 0.550)	0.039
Orkney	-0.374 (-0.507, -0.240)	0.068	-0.030 (-0.119, 0.059)	0.045
Shetland	-0.490 (-0.622, -0.359)	0.067	-0.094 (-0.192, 0.003)	0.050
Mortality within 5 years				
Alive	Reference			
Dead	0.444 (0.400, 0.488)	0.023	0.662 (0.639 - 0.684)	0.011
Comorbidity				
no comorbidities	Reference			
1 comorbidity	0.495 (0.382, 0.607)	0.057	0.358 (0.277, 0.438)	0.041
>1 comorbidities	0.786 (0.631, 0.942)	0.079	0.774 (0.702, 0.845)	0.037

Table 3. Average annual costs per patient hospitalised with AF by sex

Sex	Cost estimates	
	Mean total cost (%)	95% CI
Male		
Inpatient	3004 (80.06)	(2983, 3025)
Outpatient	314 (8.38)	(312, 317)
Care home	172 (4.58)	(160, 184)
PIS	247 (6.59)	(245, 250)
Total	3752	(3726, 3779)
Female		
Inpatient	3079 (77.60)	(3058, 3100)
Outpatient	311 (7.83)	(308, 313)
Care home	279 (7.03)	(265, 292)
PIS	263 (6.63)	(261, 266)
Total	3968	(3940, 3996)

For peer review only - http://bmjopen.bmj.com/site/about/guidelines.xhtml

For peer review only

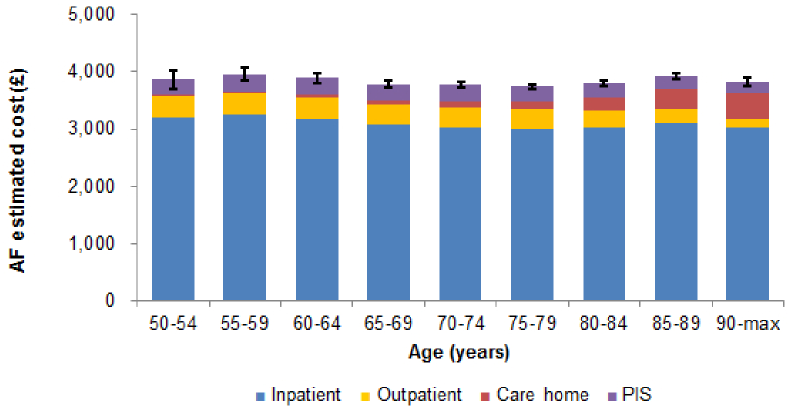

Age group	Inpatient total cost (£) Mean (95% CI)	Outpatient total cost (£) Mean (95% CI)	Care home total cost (£) Mean (95% CI)	PIS total cost (£) Mean (95% CI)
(50-54)	3198 (3065, 3331)	371 (358, 385)	20 (9, 48)	275 (257, 293)
(55-59)	3263 (3167, 3360)	373 (364, 382)	23 (2, 44)	295 (282, 307)
(60-64)	3173 (3108, 3237)	367 (360, 374)	49 (29, 69)	297 (288, 306)
(65-69)	3071 (3021, 3121)	365 (360, 370)	55 (38, 71)	289 (283, 295)
(70-74)	3028 (2989, 3067)	357 (353, 361)	98 (81, 115)	285 (281, 290)
(75-79)	3013 (2981, 3044)	341 (337, 345)	122 (107, 137)	270 (266, 273)
(80-84)	3019 (2986, 3051)	300 (297, 304)	232 (214, 250)	249 (246, 252)
(85-89)	3094 (3057, 3131)	250 (246, 254)	352 (328, 375)	226 (223, 229)
(90-max)	3023 (2977, 3069)	161 (157, 165)	449 (417, 480)	192 (188, 196)
Total	3042 (3027, 3057)	313 (311, 314)	235 (226, 244)	255 (253, 257)

185x159mm (96 x 96 DPI)

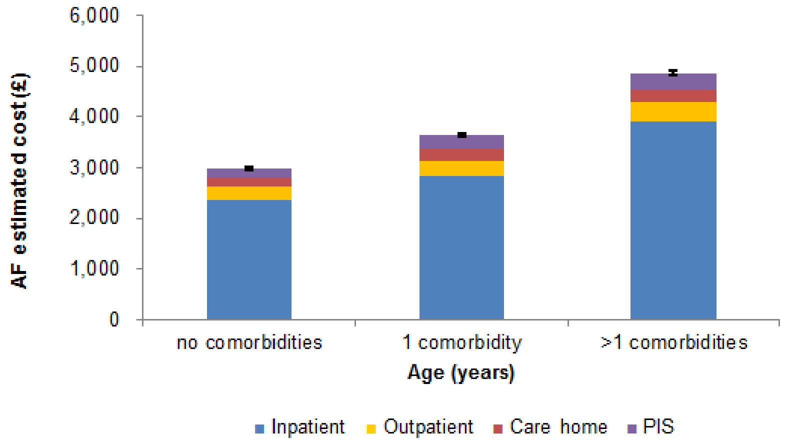

Component	No comorbidity cost (£) Mean (95% CI)	1 comorbidity cost (£) Mean (95% CI)	> 1 comorbidity cost (£) Mean (95% CI)
Inpatient	2346 (2325, 2368)	2,830 (2805, 2858)	3,903 (3873, 3934)
Outpatient	279 (277, 282)	293 (290, 298)	379 (376, 383)
Care home	191 (174, 207)	259 (240, 278)	244 (230, 258)
PIS	180 (177, 182)	250 (247, 258)	341 (337, 345)
Total	2994 (2965, 3023)	3,643 (3609, 3678)	4,909 (4870, 4948)

175x128mm (96 x 96 DPI)

ONLINE SUPPLEMENT

Table I. **Regression interactions: probability of healthcare resources utilisation and cost estimation**

Covariates	Probability (1st modelling part)		Cost Ratios (2nd modelling part)	
	Coefficient (95% CI)	Std. Err	Coefficient (95% CI)	Std. Err
Interaction: SIMD - Mortality within 5 years				
1	Reference			
2	-0.023 (-0.079, 0.034)	0.029	0.056 (0.026, 0.086)	0.015
3	0.037 (-0.019, 0.093)	0.029	0.064 (0.032, 0.095)	0.016
4	0.005 (-0.051, 0.062)	0.029	0.081 (0.049, 0.112)	0.016
5	0.041 (-0.018, 0.099)	0.030	0.092 (0.060, 0.124)	0.016
Interaction: age (year) - Charlson score (1 comorbidity)				
50-54	Reference			
55-59	-0.212 (-0.348, -0.076)	0.069	-0.065 (-0.159, 0.030)	0.048
60-64	-0.289 (-0.419, -0.158)	0.067	-0.100 (-0.191, -0.010)	0.046
65-69	-0.329 (-0.454, -0.204)	0.064	-0.145 (-0.233, -0.057)	0.045
70-74	-0.338 (-0.461, -0.215)	0.063	-0.186 (-0.272, -0.100)	0.044
75-79	-0.399 (-0.520, -0.277)	0.062	-0.180 (-0.265, -0.096)	0.043
80-84	-0.421 (-0.543, -0.300)	0.062	-0.193 (-0.278, -0.107)	0.044
85-89	-0.471 (-0.596, -0.347)	0.064	-0.199 (-0.287, -0.111)	0.045
90-max	-0.536 (-0.667, -0.406)	0.066	-0.251 (-0.345, -0.158)	0.048
Interaction: age (year) - Charlson score (>1 comorbidities)				
50-54	Reference			
55-59	-0.121 (-0.304, 0.062)	0.093	0.005 (-0.083, 0.093)	0.045
60-64	-0.117 (-0.290, 0.057)	0.088	-0.109 (-0.191, -0.027)	0.042
65-69	-0.156 (-0.323, 0.011)	0.085	-0.222 (-0.301, -0.143)	0.040
70-74	-0.234 (-0.398, 0.070)	0.084	-0.306 (-0.382, -0.229)	0.039
75-79	-0.350 (-0.513, 0.188)	0.083	-0.342 (-0.417, -0.266)	0.039
80-84	-0.440 (-0.603, 0.278)	0.083	-0.410 (-0.487, -0.334)	0.039
85-89	-0.550 (-0.715, -0.386)	0.084	-0.447 (-0.525, -0.369)	0.040
90-max	-0.657 (-0.826, -0.488)	0.086	-0.590 (-0.674, -0.506)	0.043

For peer review only - http://bmjopen.bmj.com/site/about/guidelines.xhtml

Equation I. Probability of Healthcare Utilisation

$$\Pr(HCE_{it} > 0) = \alpha + \beta_1 A_{it} + \beta_2 G_i + \beta_3 Y_i + \sum_{s=2}^{5} \beta_4 S_i + \sum_{u=2}^{8} \beta_5 U_i + \sum_{h=2}^{14} \beta_6 H_i$$

$$+ \sum_{c=2}^{3} \beta_7 C_{it} + \beta_8 D_i + \left(\beta_9 SD \sum_{s=2}^{5} SD_i * D_i \right) + \left(\beta_{10} CA \sum_{c=2}^{3} C_{it} * A_{it} \right) + u_i$$

Where: A is age at the time of admission (reference category: 50 -54 years); G is sex (reference category: male); Y is year of admission; S is SIMD quintile (reference category: most deprived quintile (1)); U is the urban/rural classification (reference category: large urban area); H is health board of inpatient admission (reference category: Greater Glasgow & Clyde); C is the Charlson comorbidity index (reference category: no comorbidities); D is mortality during five-year follow-up, SD is the interaction between SIMD and mortality; CA is the interaction between comorbidity and age; u_i is the error term for patient *i* at time *t*.

Equation II. Cost Estimation

$$E[HCE] = g(x\beta)$$

Where xβ is the linear predictor for HCE

Equation III. Multiplying First and Second Part

$$E[HCE|X] = \Pr(HCE > 0|X) * E[HCE|HCE > 0, X]$$

For peer review only - http://bmjopen.bmj.com/site/about/guidelines.xhtml

STROBE 2007 (v4) Statement—Checklist of items that should be included in reports of *cohort studies*

Section/Topic	Item #	Recommendation	Reported on page #
**Title and abstract**	1	(*a*) Indicate the study's design with a commonly used term in the title or the abstract	1
		(*b*) Provide in the abstract an informative and balanced summary of what was done and what was found	1
**Introduction**			
Background/rationale	2	Explain the scientific background and rationale for the investigation being reported	3
Objectives	3	State specific objectives, including any prespecified hypotheses	3
**Methods**			
Study design	4	Present key elements of study design early in the paper	4
Setting	5	Describe the setting, locations, and relevant dates, including periods of recruitment, exposure, follow-up, and data collection	4
Participants	6	(*a*) Give the eligibility criteria, and the sources and methods of selection of participants. Describe methods of follow-up	4
		(*b*) For matched studies, give matching criteria and number of exposed and unexposed	n/a
Variables	7	Clearly define all outcomes, exposures, predictors, potential confounders, and effect modifiers. Give diagnostic criteria, if applicable	7-8
Data sources/ measurement	8*	For each variable of interest, give sources of data and details of methods of assessment (measurement). Describe comparability of assessment methods if there is more than one group	n/a
Bias	9	Describe any efforts to address potential sources of bias	7
Study size	10	Explain how the study size was arrived at	4
Quantitative variables	11	Explain how quantitative variables were handled in the analyses. If applicable, describe which groupings were chosen and why	7-8
Statistical methods	12	(*a*) Describe all statistical methods, including those used to control for confounding	6-8
		(*b*) Describe any methods used to examine subgroups and interactions	6-7
		(*c*) Explain how missing data were addressed	n/a
		(*d*) If applicable, explain how loss to follow-up was addressed	n/a
		(*e*) Describe any sensitivity analyses	n/a
**Results**			

For peer review only

Participants	13*	(a) Report numbers of individuals at each stage of study—eg numbers potentially eligible, examined for eligibility, confirmed eligible, included in the study, completing follow-up, and analysed	n/a
		(b) Give reasons for non-participation at each stage	n/a
		(c) Consider use of a flow diagram	n/a
Descriptive data	14*	(a) Give characteristics of study participants (eg demographic, clinical, social) and information on exposures and potential confounders	n/a
		(b) Indicate number of participants with missing data for each variable of interest	n/a
		(c) Summarise follow-up time (eg, average and total amount)	n/a
Outcome data	15*	Report numbers of outcome events or summary measures over time	n/a
Main results	16	(*a*) Give unadjusted estimates and, if applicable, confounder-adjusted estimates and their precision (eg, 95% confidence interval). Make clear which confounders were adjusted for and why they were included	9-11
		(*b*) Report category boundaries when continuous variables were categorized	n/a
		(*c*) If relevant, consider translating estimates of relative risk into absolute risk for a meaningful time period	n/a
Other analyses	17	Report other analyses done—eg analyses of subgroups and interactions, and sensitivity analyses	9
**Discussion**			
Key results	18	Summarise key results with reference to study objectives	11
**Limitations**			
Interpretation	20	Give a cautious overall interpretation of results considering objectives, limitations, multiplicity of analyses, results from similar studies, and other relevant evidence	12-14
Generalisability	21	Discuss the generalisability (external validity) of the study results	14
**Other information**			
Funding	22	Give the source of funding and the role of the funders for the present study and, if applicable, for the original study on which the present article is based	15

*Give information separately for cases and controls in case-control studies and, if applicable, for exposed and unexposed groups in cohort and cross-sectional studies.

Note: An Explanation and Elaboration article discusses each checklist item and gives methodological background and published examples of transparent reporting. The STROBE checklist is best used in conjunction with this article (freely available on the Web sites of PLoS Medicine at http://www.plosmedicine.org/, Annals of Internal Medicine at http://www.annals.org/, and Epidemiology at http://www.epidem.com/). Information on the STROBE Initiative is available at www.strobe-statement.org.

For peer review only

BMJ Open

Inpatient, Outpatient, Prescribing and Care Home Costs associated with Atrial Fibrillation

Journal:	*BMJ Open*
Manuscript ID	bmjopen-2018-028575.R1
Article Type:	Original research
Date Submitted by the Author:	23-Jul-2019
Complete List of Authors:	Ciminata, Giorgio; University of Glasgow Institute of Health and Wellbeing, Health Economics and Health Technology Assessment (HEHTA)<br>Geue, Claudia; University of Glasgow, Institute of Health and Wellbeing<br>Langhorne, Peter; University of Glasgow, Institute of Cardiovascular and Medical Sciences<br>Wu, O; University of Glasgow
**Primary Subject Heading**:	Health economics
Secondary Subject Heading:	Cardiovascular medicine
Keywords:	HEALTH ECONOMICS, Atrial Fibrillation, Stroke < NEUROLOGY, Cost Analysis

SCHOLARONE™
Manuscripts

I, the Submitting Author has the right to grant and does grant on behalf of all authors of the Work (as defined in the below author licence), an exclusive licence and/or a non-exclusive licence for contributions from authors who are: i) UK Crown employees; ii) where BMJ has agreed a CC-BY licence shall apply, and/or iii) in accordance with the terms applicable for US Federal Government officers or employees acting as part of their official duties; on a worldwide, perpetual, irrevocable, royalty-free basis to BMJ Publishing Group Ltd ("BMJ") its licensees and where the relevant Journal is co-owned by BMJ to the co-owners of the Journal, to publish the Work in this journal and any other BMJ products and to exploit all rights, as set out in our licence.

The Submitting Author accepts and understands that any supply made under these terms is made by BMJ to the Submitting Author unless you are acting as an employee on behalf of your employer or a postgraduate student of an affiliated institution which is paying any applicable article publishing charge ("APC") for Open Access articles. Where the Submitting Author wishes to make the Work available on an Open Access basis (and intends to pay the relevant APC), the terms of reuse of such Open Access shall be governed by a Creative Commons licence – details of these licences and which Creative Commons licence will apply to this Work are set out in our licence referred to above.

Other than as permitted in any relevant BMJ Author's Self Archiving Policies, I confirm this Work has not been accepted for publication elsewhere, is not being considered for publication elsewhere and does not duplicate material already published. I confirm all authors consent to publication of this Work and authorise the granting of this licence.

For peer review only

Inpatient, Outpatient, Prescribing and Care Home Costs associated with Atrial Fibrillation

Authors: Ciminata G (Research Assistant)[1], Geue C (Lecturer in Health Economics)[1], Langhorne P (Professor of Stroke Care)[2], Wu O (Professor of Health Technology Assessment)[1].

1. Health Economics and Health Technology Assessment (HEHTA), Institute of Health & Wellbeing, University of Glasgow, Glasgow G12 8RZ, UK
2. Institute of Cardiovascular & Medical Sciences, University of Glasgow, Glasgow G31 2ER, UK

Corresponding author:
Mr Giorgio Ciminata (MSc), g.ciminata.1@research.gla.ac.uk

Cover title: Inpatient, Outpatient, Prescribing and Care Home Costs associated with Atrial Fibrillation

Total number of tables and figures: Figures: 2, Tables: 3

Subject terms: Atrial Fibrillation

Key words: Atrial Fibrillation, Stroke, cost analysis

Word count: 3778

For peer review only

Abstract

Objective: To estimate inpatient, outpatient, prescribing and care-home costs associated with atrial fibrillation (AF), using population-based, individual-level linked data.

Design: A two-part model was employed to estimate the probability of resource utilisation and costs conditional on positive utilisation using individual-level linked data.

Settings: Scotland, five years following first hospitalisation for AF between 1997 and 2015.

Participants: Patients aged ≥50 years, hospitalised with a known diagnosis of AF or atrial flutter.

Primary and secondary outcome measures: Inpatient, outpatient, prescribing and care home costs.

Results: The mean annual cost associated with an AF patient was estimated at £3861 (95% CI £3842-£3880). Inpatient admissions and outpatient visits accounted for 77% and 5% of total costs, respectively; prescriptions and care home stay accounted for 4% and 14% of total costs. Inpatient costs was the main driver across all age groups. While inpatient cost contributions (~80%) were constant between 50 and 84 years, they decreased for patients over 85 years. This is offset by increasing care-home cost contributions. Mean annual costs associated with AF increased significantly with increasing number of comorbidities.

Conclusion: This study used a contemporary and representative cohort, and a comprehensive approach to estimate costs associated with AF, taking into account resource utilisation beyond hospital care. While overall costs, considerably affected by comorbidity, did not increase with increasing age, care-home costs increased proportionally with age. Inpatient admission was the main contributor to the overall financial burden of AF, highlighting the need for improved mechanisms of early diagnosis to prevent hospitalisations.

Article summary

Strengths and limitations of this study

- Costs are estimated through an incidence-based approach using patient-level morbidity records.
- Sufficient follow-up time is used to capture all relevant costs to generate a contemporary estimate of health and care home costs related to AF.
- Scotland offers a robust record linkage system, where administrative patient-level health data are routinely collected.
- Data on primary care consultations were not available for linkage at a national level, however the impact this might have on overall costs is expected to be small.
- The potential risk of AF going undiagnosed and clinical miscoding of morbidity records may lead to an underestimation of the AF cohort and associated costs.

For peer review only

Introduction

Atrial Fibrillation (AF) is the most common form of arrhythmia. In Scotland AF affects 1.8% of the adult population, and rises to 6% among those aged 65 years or over [1]. In an ageing population, AF has a substantial impact on the economic burden of the healthcare system.

A number of cost analyses on estimating the economic burden of AF exist. The majority of these studies used various definition of the AF study population , based on data sourced from administrative database [2-4], health insurance databases [2, 5-7], hospital records [8, 9] and surveys [10]. Direct medical costs related to inpatient admissions, outpatient visits, as well as prescriptions have been included in these estimates; [2-10] indirect costs related to loss of productivity have been estimated among patients who were at working ages [6, 7].

Many of these studies included relatively young patients – those aged 18-20 years or older [2, 4-6, 8-10], or those under the age of 65 years [7]. However, the prevalence of AF increases significantly with age, and is most affected by patients who are older than 50 years; in patients under the age of 50, AF is often associated with structural heart disease, hyperthyroidism, or alcohol excess [11]. Hence, inclusion of younger patients and the exclusion of older patients may result in imprecise cost estimates.

There is a lack of generalisable studies based on large national population datasets that examine the total and the distribution of costs associated with AF [12]. The aim of this study was to quantify the inpatient, outpatient, prescribing and care home costs associated with AF over a five-year period. Using record-linkage of national datasets from Scotland, we also examined the distribution of costs that are attributable to AF.

For peer review only

Methods

Cost analyses or cost of illness studies typically adopt either the prevalence or incidence based approaches [13]. In the context of AF, the prevalence based approach determines costs attributable to all cases of AF in a given year, while the incidence based approach determines costs of new cases of AF in a given time period. In the present study, costs were estimated with an incidence-based approach.

Data

Data were obtained from the Information Services Division (ISD) of NHS Scotland as part of a wider project that used routinely collected data to evaluate clinical effectiveness and cost-effectiveness of Direct Oral Anticoagulants (DOACs) in the prevention of stroke in the AF population. Inpatient records for patients with a diagnosis of AF or atrial flutter between 1997 and 2015 were extracted from the General Acute Inpatient and Day Case Scottish Morbidity Records 01 (SMR01). These records contain all general acute admissions, categorized as inpatients or day cases, discharged from non-obstetric and non-psychiatric specialties [14]. Incident AF events (ICD10 code I48) were identified using all six diagnostic positions in SMR01, with a look back period of five years to minimise double counting. After checking for data entry errors and removal of duplicate records, an initial AF cohort consisting of 279,883 individuals hospitalised with a diagnosis of AF or atrial flutter was identified.

For the purpose of this analysis, only patients aged 50 years or older were included. Based on clinical advice and the evidence that prevalence and incidence of AF typically increase exponentially from 50 years onwards [11], the analysis including 50 years age group at the lowest range would be inclusive of all patients potentially at risk of AF.

For peer review only

The choice on the age cut-off for our AF cohort was also based on the indication of oral anticoagulants for the AF population. Most AF patients in our cohort are also on direct oral anticoagulants, and patients who are 50 years or older are likely to be on anticoagulants only because of AF, while patients younger than 50 (only about 3% of AF patients in our cohort) could be on anticoagulants for reasons other than AF. The final dataset for analysis consisted of 272,716 patients.

Individual-level data linkage was then carried out with outpatient clinic attendance (Outpatient Attendance Scottish Morbidity Records 00; SMR00), the prescribing information system (PIS), care home census and mortality records (National Records for Scotland, NRS). Records from SMR00 include information on new and follow up outpatient appointments for any clinical specialty [15]. The PIS database includes prescribing records for all medicines and their associated costs, which are prescribed and dispensed by community pharmacies, dispensing doctors and a small number of specialist appliance suppliers [16]. The quality of PIS data is guaranteed by an electronic data capture, and it passes several stages of quality control before and after data are submitted [17]. The care home census combines the former Residential Care Home Census (run by the Scottish Government) and the Private Nursing Homes Census (run by ISD Scotland). Items reported in the care home census include discharge dates to care home residency such as NHS and private nursing homes, as well as an indication on whether nursing care is required [16].

Patients were followed up for five years following incident AF event in terms of their healthcare resource use, care home admissions and mortality. Since AF is often a precursor of stroke and cardiovascular conditions, an estimation of costs for a period of five years post AF event would allow us to fully capture costs associated with an AF patient. **Costing**

For peer review only

Inpatient care costs were obtained from the latest (2013/2014) Scottish National Tariff (SNT), a list of standard average prices based on Healthcare Resource Groups (HRGs) [17, 18]. The SNT uses HRG4 for grouping clinically similar treatments that use similar levels of healthcare resources. After defining a total cost per episode, the total cost for a continuous inpatient stay (CIS) was calculated. A CIS describes the entire duration of an inpatient stay from the date of admission to the date of discharge and can consist of several episodes in different specialties. Since the SNT is based on spells of care (inpatient stay within the same specialty) rather than individual inpatient episodes or a CIS, a CIS was partitioned into spells when a change in specialty occurred [17]. If within a CIS, two or more episodes were in the same specialty, only the highest incurred cost was taken into account, and the remaining episodes were replaced with a zero cost. Outpatient costs were obtained by assigning outpatient specialty costs, to outpatient attendances [17]. Unit costs were specific to whether the outpatient attendance took place at a consultant or nurse led clinic [15].

The cost of each prescription dispensed per patient was obtained from PIS [19]. Firstly, the price per unit was obtained by dividing the item price by the pack size. Secondly, the total number of items dispensed was obtained by multiplying the number of items dispensed by the number of instalments. Care home costs, obtained from the care home census, were based on length of stay or residency. Care home residency was established from care home census records, reporting admission to a care home like structure [16]. An average of care home charges for long stay residents was calculated using information on whether nursing care was provided or not. The average weekly care home charge was expressed per day, so that only the effective days spent in a care home were costed. The tariffs used for costing account for inflation, therefore further cost adjustment was not needed.

For peer review only

Econometric model

Healthcare expenditure data are typically characterised by: i) a significant proportion of zero-cost observations for individuals who have not utilised any healthcare resources in a given time period, and ii) a skewed distribution for positive costs. A two-part model was used [20, 21].

In the first part of the model, the probability of using a healthcare service in a given time period was estimated using a probit model (Equation I, please see online supplement). The same explanatory variables were used in the second part of the model, with a gamma distribution and log link, estimating costs conditional on having incurred positive costs (Equation II, please see online supplement). Mean costs per patient per year following their incident AF event were calculated by multiplying first and second modelling part (Equation III, please see online supplement).

In order to account for the skewed nature of cost data, generalised linear models (GLMs) were used. These were compared against ordinary least squares regression (OLS) and log transformed OLS by means of the Akaike Information Criterion (AIC), which measures goodness of fit. When comparing the different models, GLM reported the lowest AIC indicating the best fit for the given set of data. A user-written STATA programme "glmdiagnostic.do" [20], performing four different tests simultaneously, was used to identify the most appropriate distributional family and link function.

Econometric model covariates

The two-part model adjusted for age, sex, year of inpatient admission, socio-economic status, urban-rural classification, health board, comorbidities and mortality. These covariates are considered to be the main confounders that have an effect on costs incurred by an AF population.

For peer review only

We controlled for age because AF and associated comorbidities are age-related conditions, and may have an impact on the overall costs. We also assumed costs to vary between males and females, in particular those for care home residency. Variation in healthcare utilisation and associated costs and care home residency by socio-economic status is controlled for using the Scottish Index of Multiple Deprivation (SIMD). The SIMD reflects areas of multiple deprivation ranked from the most to the least deprived and expressed as quintiles where the most and the least deprived areas are represented by 1 and 5 respectively [22]. In Scotland, there are 14 regional health boards responsible for the provision of healthcare [23]. Hence, potential differences in healthcare utilisation and prescribing costs, may reflect variation in clinical practice and prescribing behaviour rather than the ability of patients to access care. Patients living in urban areas may have easier access to care compared to patients living in more remote areas, which is controlled for including the 8-fold classification measuring rurality [24].

Patients with one or more comorbidities are expected to incur significantly higher costs than those with none. We accounted for this by including the Charlson comorbidity index, where 1 indicates the absence of comorbidities, 2 the presence of only a single comorbidity and 3 the presence of more than one comorbidity [25]. Two interaction terms between age and comorbidities, and mortality and SIMD were included in the econometric model. Intuitively, a relationship of direct proportionality between age and comorbidities suggests that the level of comorbidities increases, as patients get older. Similarly, the socio-economic status may significantly influence the rate of socio-economic inequalities in mortality [26].

For peer review only

Sensitivity analyses

In order to ascertain whether mortality had an impact on overall AF related healthcare costs, average annual cost per patient by age and for each health or care home sector, was estimated for patients who were alive and those who were dead at the end of the five-year follow-up period. The two econometric models (Equation IV and V, please see online supplement) followed the same structure of the model described in the previous section and used for the main analysis; however, those models were not adjusted for mortality.

Ethics statement

The authors state that no ethical approval was needed.

Patients and public involvement

There was no patients or public involvement

Results

Cohort characteristics

Of the 272,716 AF patients with a mean age of 71 years (SD 10.6), the majority were identified in the two largest urban health board areas (Greater Glasgow & Clyde and Lothian), accounting for 22.3% and 14.9% respectively. This is also reflected in our categorisation of geographical areas, where large urban represented 38.6% and other urban areas represented 29.8% of the total AF cohort. Greater proportion of patients live in areas belonging to the most deprived quintile compared with those living in the least deprived areas – SIMD quintile 1 and quintile 5 representing 22.6% and 16.4% of the AF cohort respectively (Table1).

For peer review only

Econometric modelling results

Regression results for both modelling parts are presented in Table 2. Overall, an inversely U-shaped association between age and the likelihood of utilising any health or social care services was observed – a gradual increment in the likelihood in resource use with advancing age up to 80 years, when compared with the reference group (50-54 years), while patients 80 years or older showing a decreased probability of utilising healthcare services. However, this association was not observed in the second modelling part model, estimating costs conditional on having incurred positive costs, where a statistically significant gradient between age and costs indicated increasing costs as the cohort ages. The use of health or social care services and associated costs also increased significantly for patients living in the most deprived areas, when compared with patients living in areas with the lowest level of deprivation. The effect of socio-economic status on healthcare utilisation was also measured for those who are alive at the end of the five-year follow-up period through an interaction term between SIMD and mortality, but no statistically significant effect was found. Full details of regression results for interaction terms are presented in the supplementary online material (Table I, please see online supplement).

For patients with comorbidities, the probabilities of utilising healthcare services were 49.5% (one comorbidity) and 78.6% (two or more comorbidities) greater than the probability for those with no comorbidities. Although healthcare utilisation increased with the number of comorbidities, the interaction term between age and comorbidities indicated that as patients get older the use of healthcare services on average is lower for patients with one or more comorbidities than those with none. The decrease in healthcare utilisation by age is more pronounced in patients with more comorbidities than in those with only one comorbidity.

For peer review only

The difference in healthcare costs between comorbidity categories indicated that in the presence of one or more comorbidities, on average healthcare costs decrease as patients get older.

Full details of regression results for patients who were alive and those who were dead at the end of the five-year follow-up period are presented in the supplementary online material (Table II - V, please see online supplement).

Cost estimates

The estimated mean annual cost per AF patient was £3861 (95% CI: £3842-£3880). The estimated total costs and distribution of costs according to sex are shown in Table 3. While there is little difference between the total costs and the distribution of costs for inpatient, outpatient and prescription costs, the difference seems more pronounced when comparing the care home component of costs (5% of total costs among males vs 7% of total costs among females).

The average annual cost per AF patient by age and for each health or care home sector is shown in Figure 1. Considering the individual contribution of each cost component to the overall costs, inpatient costs was the main driver across all age groups. While inpatient cost contribution remained constant with an average contribution of about 80% to the overall cost for patients aged between 50 and 84 years, it decreased for patients over 85 years of age. Similar patterns were observed for outpatient and prescribing costs. On the contrary, the contribution of care home costs to the overall costs increased with age (0.5% for patients aged 50-54 years and approximately 11% for patients who are 90 years or older).

For peer review only

The contribution of each setting to the total health and care home costs by the number of existing comorbidities is illustrated in Figure 2. While inpatient and total costs vary considerably with the number of comorbidities, outpatient and care home contributions remain fairly constant.

The estimated mean annual cost per AF patient alive at the end of the five-year follow-up period was £3110 (95% CI: £3090-£3131). The average annual cost per AF patient by age and for each health or care home sector is presented in the supplementary online material (Figure I, please see online supplement). For these patients, inpatient costs was the main driver across all age groups; a gradient between age and costs indicated increasing costs as the cohort ages. Similar patterns were observed for care home costs. On the contrary, outpatient and prescribing costs remained constant up to 74 years, but decreased slightly for older patients.

The estimated mean annual cost per AF patient who died during the five-year follow-up period, was £2299 (95% CI: £2279-£2319) (Figure II, please see online supplement). For these patients, inpatient costs was the main driver across all age groups; a gradient between age and costs indicated decreasing costs as the cohort ages. This was also observed for outpatient and prescribing costs; but care home costs on average increased across age groups.

For peer review only

Discussion

A greater proportion of AF patients were found in areas with the highest index of deprivation. This, combined with the likelihood for people living in the most deprived quintile having longer inpatient stays due to a lack of support at home, may explain the difference in inpatient care utilisation between patients from the most and the least deprived areas, with associated costs being higher for the former group. As AF is more likely to affect the elderly, AF related costs were expected to increase with age. As health deteriorates with age, older age groups are assumed to make greater use of healthcare services, and therefore incur higher costs than younger age groups. However, age was found to have a modest impact on overall healthcare costs, being fairly consistent across age groups. This finding is in line with existing evidence indicating that healthcare expenditure depends not only on patients' calendar age, but is also significantly associated with remaining lifetime [27].

Any observed correlation between healthcare expenditure and age may therefore be attributable to the fact that the proportion of patients who are at the end of their lives is substantially greater in older rather than younger age groups [27]. On the other hand, comorbidity had a considerable effect on the overall cost, increasing significantly in patients with more than one comorbidity. However, the decrease in healthcare utilisation by age is more pronounced in patients with more comorbidities than in those with only one comorbidity. Decreasing inpatient and outpatient costs for the oldest patients were offset by increasing care home costs, in particular for women. Indeed, the main cause for higher overall costs incurred by women is attributable to the higher likelihood for elderly women to reside in care homes. Interestingly, care home contribution to the overall costs was noticeably lower for patients with multiple comorbidities than for those with none or one comorbidity. This may suggest that sicker patients are more likely to be in hospital than in a care home.

For peer review only

To date, only one single study published in 2004 has estimated the cost of AF in Scotland; the authors estimated the cost of AF in 1995/1996, and projected these to the year 2000 [28]. Previous work has focussed on a 12-months follow-up, which seems limited in order to capture all healthcare resource utilisation for AF patients. Our study offers a longer follow-up and a contemporary estimate of healthcare costs related to AF including all relevant care settings. Our study offers a distinct advantage over previous work as costs, rather than being based on extrapolated rates using a prevalence-based approach [28], are estimated with an incidence-based method using patient-level morbidity records. Using an incidence based approach to costing and a broad perspective to capture the majority of costs associated with AF, several routinely collected administrative datasets from Scotland were combined, including care home utilisation.

Existing studies, including ours, regardless of econometric model choice and covariates used, show that costs due to inpatient admission are the main contributor to overall AF related healthcare cost. This is a pertinent finding that may well support future policies on opportunistic screening in the population at risk of AF, and in particular in Scotland where 1 in 3 patients with AF are currently undiagnosed [29]. The European AF management guidelines and the Scottish Cross-Party Group 'Heart Disease and Stroke', recently recommended that people who are 65 years or older and at risk of AF and associated comorbidities such as cardiovascular disease, diabetes or respiratory disease should be screened opportunistically in primary care, pharmacies or community settings [29, 30]. With rigorous screening and appropriate treatment, hospitalisations could be avoided and costs reduced.

For peer review only

Although we have captured most healthcare sectors and related costs, we were not able to obtain national data on primary care consultations, as these data are currently not routinely available for linkage in Scotland. Not capturing these data, may lead to an underestimation of the size of the AF cohort and associated costs.

However, the costs associated with primary care consultations is expected to have a limited impact on the overall total AF related costs. Such underestimation could also result from AF going undiagnosed and clinical miscoding of morbidity records. Nevertheless, by using a cohort of patients hospitalised with AF we were able to capture more severe cases of AF. Prescribing and care home data were only available respectively from 2009 to 2012, their contribution to overall AF related costs might also be underestimated. Other limitations are inherent to the nature of administrative data, such as missing records or incomplete data.

Further, we acknowledge the issue concerning attributing AF related costs to patients with a structural heart disease, as AF may manifest subsequently because of this. In our analysis, we identified about 14% of AF patients with a structural heart disease; these were patients with systolic dysfunction, valvular heart disease or heart valve replacement. However, from the hospital data it was not possible to establish causation between structural heart disease and AF.

Recognising these limitations, we were nevertheless able to harness high quality patient-level linked data to identify a cohort of AF patients and to estimate AF related costs in Scotland.

For peer review only

The inclusion of all available cost components is crucial for establishing overall costs, as these often extend beyond hospitalisation. The study identifies hospitalisation as the main cost driver and suggests that the implementation of AF screening policies could substantially reduce AF related health care costs. Most importantly, the study concludes that patient's age has a limited impact on the overall AF related cost, and therefore may contribute much less to future growth of AF related cost in an ever-ageing Scottish population .

Future work will be able to utilise Scottish Stroke Care Audit (SSCA) records, allowing for the identification of additional AF patients; these are patients hospitalised with a stroke, where AF has been recorded in audit data as an underlying comorbidity. Being able to complement inpatient records with SSCA records will allow us to capture more AF patients in Scotland. Moreover, future research may be able to include indirect costs associated with productivity-loss by linking morbidity and prescribing data to national data from the Department for Work and Pensions, for instance.

For peer review only

Acknowledgements: We thank the members of the Information Services Division, National Services Scotland, for their support. We acknowledge the support from The Farr Institute @ Scotland. The Farr Institute @ Scotland is supported by a 10-funder consortium: Arthritis Research UK, the British Heart Foundation, Cancer Research UK, the Economic and Social Research Council, the Engineering and Physical Sciences Research Council, the Medical Research Council, the National Institute of Health Research, the National Institute for Social Care and Health Research (Welsh Assembly Government), the Chief Scientist Office (Scottish Government Health Directorates), the Wellcome Trust (MRC Grant No: MR/K007017/1).

Author contributions: GC, CG, PL and OW conceived the article. GC carried out the statistical analysis and prepared the first draft of the manuscript. GC, CG, PL and OW contributed to editing the manuscript and approved the final version submitted for publication.

Funding sources: This work was supported by Farr Institute for Health Informatics Research, Scotland, MRC, grant number MR/K007017/1

Competing interest: The authors declare no conflict of interest.

Data Statement: All data underlying the analyses are confidential and subject to disclosure control. Data can only be obtained through application to Information Services Division (ISD) via the Public Benefit and Privacy Panel (PBPP).

For peer review only

References

1. Scotland, H.I. *Prevention of stroke in patients with atrial fibrillation*. 2013 [cited 2016 January 2016]; Available from: http://www.sign.ac.uk/assets/af_publication.pdf.
2. Bennell, M.C., et al., *Identifying predictors of cumulative healthcare costs in incident atrial fibrillation: a population-based study.* J Am Heart Assoc, 2015. **4**(4).
3. Hallinen, T., et al., *Direct costs of warfarin treatment among patients with atrial fibrillation in a Finnish health care setting.* Curr Med Res Opin, 2006. **22**(4): p. 683-92.
4. Nelson, W.W., et al., *Out-of-range international normalized ratio values and healthcare cost among new warfarin patients with non-valvular atrial fibrillation.* J Med Econ, 2015. **18**(5): p. 333-40.
5. Ghate, S.R., et al., *All-cause and bleeding-related health care costs in warfarin-treated patients with atrial fibrillation.* J Manag Care Pharm, 2011. **17**(9): p. 672-84.
6. Rohrbacker, N.J., et al., *The burden of atrial fibrillation and other cardiac arrhythmias in an employed population: associated costs, absences, and objective productivity loss.* J Occup Environ Med, 2010. **52**(4): p. 383-91.
7. Wu, E.Q., et al., *Economic burden and co-morbidities of atrial fibrillation in a privately insured population.* Curr Med Res Opin, 2005. **21**(10): p. 1693-9.
8. Holstenson, E., et al., *Predictors of costs related to cardiovascular disease among patients with atrial fibrillation in five European countries.* Europace, 2011. **13**(1): p. 23-30.
9. Reynolds, M.R., et al., *Healthcare resource utilization and costs associated with recurrent episodes of atrial fibrillation: the FRACTAL registry.* J Cardiovasc Electrophysiol, 2007. **18**(6): p. 628-33.
10. Jonsson, L., et al., *Cost of illness and drivers of cost in atrial fibrillation in Sweden and Germany.* Appl Health Econ Health Policy, 2010. **8**(5): p. 317-25.
11. Sankaranarayanan, R., et al., *Comparison of Atrial Fibrillation in the Young versus That in the Elderly: A Review.* Cardiology Research and Practice, 2013. **2013**: p. 16.
12. Johnsen, S.P., et al., *Cost of illness of atrial fibrillation: a nationwide study of societal impact.* BMC Health Services Research, 2017. **17**(1): p. 714.
13. Tarricone, R., *Cost-of-illness analysis. What room in health economics?* Health Policy, 2006. **77**(1): p. 51-63.
14. Scotland, I.S.D. *General Acute Inpatient and Day Case - Scottish Morbidity Record (SMR01)*. 2015 [cited 2017; Available from: http://www.ndc.scot.nhs.uk/National-Datasets/data.asp?SubID=2.
15. Scotland, I.S.D. *Outpatient Attendance (SMR00)*. 2015 [cited 2015 January 2015]; Available from: http://www.ndc.scot.nhs.uk/National-Datasets/data.asp?SubID=2.
16. Scotland, I.S.D. *Care Home Census 2015*. 2015 November 2015]; Available from: http://www.isdscotland.org/Health-Topics/Health-and-Social-Community-Care/Care-Homes/Previous-Publications/.
17. Scotland, I.S.D. *Scottish National Tariff*. 2015 [cited 2015 December 2015]; Available from: http://www.isdscotland.org/Health-Topics/Finance/Scottish-National-Tariff/.
18. Groups, H.R. *Healthcare Resource Groups*. 2015 November 2015]; Available from: http://www.hscic.gov.uk/hrg.
19. Alvarez-Madrazo, S., et al., *Data Resource Profile: The Scottish National Prescribing Information System (PIS).* International Journal of Epidemiology, 2016.

For peer review only

20. Henry, A.G., et al., *Economic Evaluation in Clinical Trials*. 'Oxford University Press': Oxford, UK.
21. Manning, W.G., A. Basu, and J. Mullahy, *Generalized modeling approaches to risk adjustment of skewed outcomes data.* Journal of Health Economics, 2005. **24**(3): p. 465-488.
22. Government, T.S. *Scottish Index of Multiple Deprivation*. 2015 [cited 2015 November 2015]; Available from: http://www.gov.scot/Topics/Statistics/SIMD.
23. Government, T.S. *NHS Boards*. 2015 [cited 2015 January 2015]; Available from: http://www.gov.scot/Topics/Health/NHS-Workforce/NHS-Boards.
24. Government, T.S. *Scottish Government Urban Rural Classification*. 2015 [cited 2015 January 2015]; Available from: http://www.gov.scot/Topics/Statistics/About/Methodology/UrbanRuralClassification.
25. Charlson, M.E., et al., *A new method of classifying prognostic comorbidity in longitudinal studies: Development and validation.* Journal of Chronic Diseases, 1987. **40**(5): p. 373-383.
26. Leyland, A.H., et al., *Cause-specific inequalities in mortality in Scotland: two decades of change. A population-based study.* BMC Public Health, 2007. **7**: p. 172.
27. Zweifel, P., S. Felder, and M. Meiers, *Ageing of population and health care expenditure: a red herring?* Health Econ, 1999. **8**(6): p. 485-96.
28. Stewart, S., et al., *Cost of an emerging epidemic: an economic analysis of atrial fibrillation in the UK.* Heart, 2004. **90**(3): p. 286-292.
29. Stroke, C.-P.G.o.H.D.a. *A Focus on Atrial Fibrillation in Scotland 2017*. 2017 March 2018]; Available from: https://www.chss.org.uk/documents/2018/01/a-focus-on-atrial-fibrillation-in-scotland-pdf.pdf.
30. Camm, A.J., et al., *2012 focused update of the ESC Guidelines for the management of atrial fibrillation: an update of the 2010 ESC Guidelines for the management of atrial fibrillation. Developed with the special contribution of the European Heart Rhythm Association.* Eur Heart J, 2012. **33**(21): p. 2719-47.

For peer review only

Figure legends

Figure 1. Average annual costs per patient hospitalised with AF by sector. Cost components with confidence interval are presented for each age group.

Figure 2. Average cost per patient hospitalised with AF by Charlson Comorbidity Index. Cost components with confidence interval are presented for each Comorbidity category.

For peer review only

Tables

Table 1. Baseline characteristics of AF patients 50 years or older

Characteristics	N (%)
Number of patients	272,716
Mean age at first admission*(SD)**(range)	71 *(10.6) **(50 -108)
Sex	
Male	135,683 (49.8)
Female	137,033 (50.2)
Health Boards	
Greater Glasgow & Clyde	60,774 (22.3)
Lothian	40,498 (14.9)
Lanarkshire	30,105 (11.0)
Grampian	25,208 (9.2)
Ayrshire & Arran	24,468 (7.9)
Tayside	21,543 (9.0)
Fife	18,584 (6.8)
Highland	17,612 (6.5)
Forth valley	13,308 (4.9)
Dumfries & Galloway	9,645 (3.5)
Borders	7,148 (2.6)
Western isles	1,812 (0.7)
Shetland	1,009 (0.4)
Orkney	1,002 (0.4)
Geography	
Large/urban	104,841 (38.4)
Other/urban	80,794 (29.6)
Accessible small towns	24,492 (9.0)
Remote small towns	8,126 (3.0)
Very remote small towns	3,712 (1.4)
Accessible rural	30,122 (11.1)
Remote rural	10,277 (3.8)
Very remote rural	9,908 (3.6)
SIMD quintile	
1	61,686 (22.6)
2	61,704 (22.6)
3	54,937 (20.1)
4	49,448 (18.1)
5	44,933 (16.4)
Comorbidity	
no comorbidity	36,345 (13.4)
1 comorbidity	52,159 (19.2)
>1 comorbidities	182,827 (67.4)
Re-hospitalised (any condition)	173,120 (63.7)
Admitted to care-home	7,091 (2.6)
Mortality	

Alive	200,446 (73.5)
Dead	72,270 (26.5)

Table 2. Regression results: probability of healthcare resources utilisation and cost estimation

Covariates	Probability (1st modelling part)		Cost Ratios (2nd modelling part)	
	Coefficient (95% CI)	Std. Err	Coefficient (95% CI)	Std. Err
Age group (years)				
50-54	Reference			
55-59	0.036 (-0.034, 0.107)	0.036	0.045 (-0.005, 0.096)	0.026
60-64	0.113 (0.045, 0.180)	0.034	0.088 (0.040, 0.136)	0.025
65-69	0.134 (0.069, 0.199)	0.033	0.120 (0.073, 0.167)	0.024
70-74	0.126 (0.062, 0.191)	0.033	0.176 (0.130, 0.221)	0.023
75-79	0.182 (0.118, 0.247)	0.033	0.183 (0.138, 0.228)	0.023
80-84	0.075 (0.009, 0.141)	0.034	0.246 (0.200, 0.292)	0.023
85-89	0.022 (-0.048, 0.091)	0.035	0.334 (0.286, 0.382)	0.024
90-max	-0.208 (-0.283, -0.132)	0.039	0.474 (0.420, 0.527)	0.027
Sex				
Male	Reference			
Female	0.029 (0.012, 0.047)	0.009	0.052 (0.042, 0.062)	0.005
Date of admission	0.173 (0.172, 0.175)	0.001	-0.023 (-0.024, -0.022)	0.001
SIMD quintile				
1	Reference			
2	0.036 (-0.012, 0.083)	0.024	-0.054 (-0.079, -0.028)	0.013
3	-0.028 (-0.075, 0.019)	0.024	-0.075 (-0.102, -0.049)	0.014
4	-0.031 (-0.078, 0.017)	0.024	-0.106 (-0.132, -0.080)	0.013
5	-0.042 (-0.090, 0.006)	0.024	-0.139 (-0.165, -0.113)	0.013
Geography				
Large urban	Reference			
Other urban	-0.144 (-0.171, -0.118)	0.013	-0.024 (-0.039, -0.010)	0.007
Accessible small towns	-0.163 (-0.197, -0.128)	0.018	-0.040 (-0.059, -0.021)	0.010
Accessible rural	-0.213 (-0.247, -0.180)	0.017	-0.044 (-0.063, -0.025)	0.010
Remote small towns	-0.160 (-0.213, -0.106)	0.027	0.002 (-0.030, 0.034)	0.016
Remote rural	-0.298 (-0.347, -0.250)	0.025	-0.038 (-0.067, -0.009)	0.015
Very remote small towns	-0.398 (-0.480, -0.315)	0.042	-0.053 (-0.105, -0.002)	0.026
Very remote rural	-0.371 (-0.434, -0.308)	0.032	-0.059 (-0.100, -0.018)	0.021
Health boards				
Great Glasgow and Clyde	Reference			
Lothian	-0.041 (-0.073, -0.010)	0.016	-0.032 (-0.049, -0.016)	0.008
Lanarkshire	0.003 (-0.031, 0.038)	0.018	-0.065 (-0.084, -0.047)	0.009
Ayrshire and Arran	-0.356 (-0.393, -0.318)	0.019	-0.044 (-0.066, -0.022)	0.011

Grampian	0.020 (-0.018, 0.058)	0.019	-0.059 (-0.079, -0.039)	0.010
Tayside	-0.401 (-0.436, -0.365)	0.018	-0.084 (-0.104, -0.063)	0.010
Fife	-0.055 (-0.099, -0.012)	0.022	-0.007 (-0.031, 0.018)	0.012
Highland	-0.156 (-0.209, -0.104)	0.027	-0.047 (-0.078, -0.016)	0.016
Forth Valley	-0.473 (-0.516, -0.431)	0.022	-0.113 (-0.139, -0.087)	0.013
Dumfries and Galloway	-0.303 (-0.353, -0.252)	0.026	-0.137 (-0.167, -0.106)	0.015
Borders	-0.487 (-0.541, -0.434)	0.027	-0.090 (-0.124, -0.056)	0.017
Western Isles	-1.075 (-1.176, -0.974)	0.052	0.473 (0.396, 0.550)	0.039
Orkney	-0.374 (-0.507, -0.240)	0.068	-0.030 (-0.119, 0.059)	0.045
Shetland	-0.490 (-0.622, -0.359)	0.067	-0.094 (-0.192, 0.003)	0.050
Mortality within 5 years				
Alive	Reference			
Dead	0.444 (0.400, 0.488)	0.023	0.662 (0.639 - 0.684)	0.011
Comorbidity				
no comorbidities	Reference			
1 comorbidity	0.495 (0.382, 0.607)	0.057	0.358 (0.277, 0.438)	0.041
>1 comorbidities	0.786 (0.631, 0.942)	0.079	0.774 (0.702, 0.845)	0.037

Table 3. Average annual costs per patient hospitalised with AF by sex

Sex	Cost estimates	
	Mean total cost (%)	95% CI
Male		
Inpatient	3004 (80.06)	(2983, 3025)
Outpatient	314 (8.38)	(312, 317)
Care home	172 (4.58)	(160, 184)
PIS	247 (6.59)	(245, 250)
Total	3752	(3726, 3779)
Female		
Inpatient	3079 (77.60)	(3058, 3100)
Outpatient	311 (7.83)	(308, 313)
Care home	279 (7.03)	(265, 292)
PIS	263 (6.63)	(261, 266)
Total	3968	(3940, 3996)

For peer review only - http://bmjopen.bmj.com/site/about/guidelines.xhtml

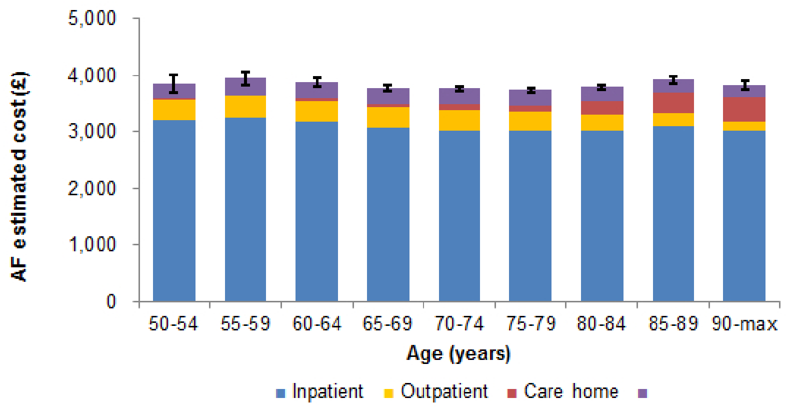

Age group	Inpatient total cost (£) Mean (95% CI)	Outpatient total cost (£) Mean (95% CI)	Care home total cost (£) Mean (95% CI)	PIS total cost (£) Mean (95% CI)
(50-54)	3198 (3065, 3331)	371 (358, 385)	20 (9, 48)	275 (257, 293)
(55-59)	3263 (3167, 3360)	373 (364, 382)	23 (2, 44)	295 (282, 307)
(60-64)	3173 (3108, 3237)	367 (360, 374)	49 (29, 69)	297 (288, 306)
(65-69)	3071 (3021, 3121)	365 (360, 370)	55 (38, 71)	289 (283, 295)
(70-74)	3028 (2989, 3067)	357 (353, 361)	98 (81, 115)	285 (281, 290)
(75-79)	3013 (2981, 3044)	341 (337, 345)	122 (107, 137)	270 (266, 273)
(80-84)	3019 (2986, 3051)	300 (297, 304)	232 (214, 250)	249 (246, 252)
(85-89)	3094 (3057, 3131)	250 (246, 254)	352 (328, 375)	226 (223, 229)
(90-max)	3023 (2977, 3069)	161 (157, 165)	449 (417, 480)	192 (188, 196)
Total	3042 (3027, 3057)	313 (311, 314)	235 (226, 244)	255 (253, 257)

57x48mm (300 x 300 DPI)

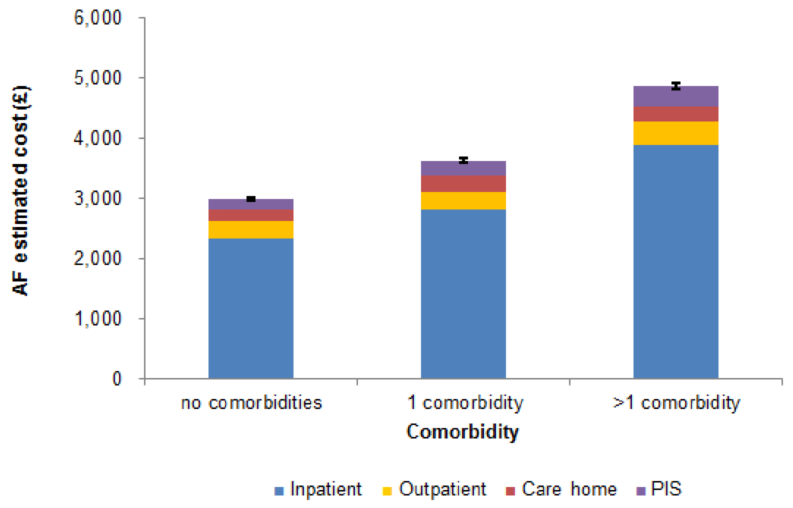

Component	No comorbidity cost (£) Mean (95% CI)	1 comorbidity cost (£) Mean (95% CI)	> 1 comorbidity cost (£) Mean (95% CI)
Inpatient	2346 (2325, 2368)	2,830 (2805, 2858)	3,903 (3873, 3934)
Outpatient	279 (277, 282)	293 (290, 298)	379 (376, 383)
Care home	191 (174, 207)	259 (240, 278)	244 (230, 258)
PIS	180 (177, 182)	250 (247, 258)	341 (337, 345)
Total	2994 (2965, 3023)	3,643 (3609, 3678)	4,909 (4870, 4948)

57x48mm (300 x 300 DPI)

ONLINE SUPPLEMENT

Equation I. Probability of healthcare utilisation

$$\Pr(HCE_{it} > 0) = \alpha + \beta_1 \sum_{s=2}^{9} A_{it} + \beta_2 G_i + \beta_3 Y_i + \beta_4 \sum_{s=2}^{5} S_i + \beta_5 \sum_{u=2}^{8} U_i + \beta_6 \sum_{h=2}^{14} H_i$$

$$+ \beta_7 \sum_{c=2}^{3} C_{it} + \beta_8 D_i + \left(\beta_9 \sum_{s=2}^{5} S_i * D_i\right) + \left(\beta_{10} \sum_{c=2}^{3} C_{it} * \sum_{s=2}^{9} A_{it}\right) + u_i$$

Where: A is age at the time of admission (reference category: 50 -54 age group); G is sex (reference category: male); Y year of admission; S is SIMD quintile (reference category: most deprived quintile (1)); U is the urban/rural classification (reference category: large urban area);H is health board of inpatient admission (reference category: Greater Glasgow & Clyde); C is the Charlson comorbidity index (reference category: no comorbidities); D is mortality during five year follow-up; u_i is the error term for patient *i* at time *t*.

Equation II. Cost estimation

$$E[HCE] = g(x\beta)$$

Where xβ is the linear predictor for HCE

Equation III. Multiplying first and second part

$$E[HCE|X] = \Pr(HCE > 0|X) * E[HCE|HCE > 0, X]$$

For peer review only - http://bmjopen.bmj.com/site/about/guidelines.xhtml

Table I. Regression interactions: probability of healthcare resources utilisation and cost estimation

Covariates	Probability (1st modelling part)		Cost Ratios (2nd modelling part)	
	Coefficient (95% CI)	Std. Err	Coefficient (95% CI)	Std. Err
**Interaction: SIMD - Mortality within 5 years**				
1	Reference			
2	-0.023 (-0.079, 0.034)	0.029	0.056 (0.026, 0.086)	0.015
3	0.037 (-0.019, 0.093)	0.029	0.064 (0.032, 0.095)	0.016
4	0.005 (-0.051, 0.062)	0.029	0.081 (0.049, 0.112)	0.016
5	0.041 (-0.018, 0.099)	0.030	0.092 (0.060, 0.124)	0.016
**Interaction: age (year) - Charlson score (1 comorbidity)**				
50-54	Reference			
55-59	-0.212 (-0.348, -0.076)	0.069	-0.065 (-0.159, 0.030)	0.048
60-64	-0.289 (-0.419, -0.158)	0.067	-0.100 (-0.191, -0.010)	0.046
65-69	-0.329 (-0.454, -0.204)	0.064	-0.145 (-0.233, -0.057)	0.045
70-74	-0.338 (-0.461, -0.215)	0.063	-0.186 (-0.272, -0.100)	0.044
75-79	-0.399 (-0.520, -0.277)	0.062	-0.180 (-0.265, -0.096)	0.043
80-84	-0.421 (-0.543, -0.300)	0.062	-0.193 (-0.278, -0.107)	0.044
85-89	-0.471 (-0.596, -0.347)	0.064	-0.199 (-0.287, -0.111)	0.045
90-max	-0.536 (-0.667, -0.406)	0.066	-0.251 (-0.345, -0.158)	0.048
**Interaction: age (year) - Charlson score (>1 comorbidities)**				
50-54	Reference			
55-59	-0.121 (-0.304, 0.062)	0.093	0.005 (-0.083, 0.093)	0.045
60-64	-0.117 (-0.290, 0.057)	0.088	-0.109 (-0.191, -0.027)	0.042
65-69	-0.156 (-0.323, 0.011)	0.085	-0.222 (-0.301, -0.143)	0.040
70-74	-0.234 (-0.398, -0.070)	0.084	-0.306 (-0.382, -0.229)	0.039
75-79	-0.350 (-0.513, -0.188)	0.083	-0.342 (-0.417, -0.266)	0.039
80-84	-0.440 (-0.603, -0.278)	0.083	-0.410 (-0.487, -0.334)	0.039
85-89	-0.550 (-0.715, -0.386)	0.084	-0.447 (-0.525, -0.369)	0.040
90-max	-0.657 (-0.826, -0.488)	0.086	-0.590 (-0.674, -0.506)	0.043

w only

For peer review only - http://bmjopen.bmj.com/site/about/guidelines.xhtml

Equation IV. Probability of healthcare utilisation (alive at the end of the five-year follow-up period)

$$\Pr(HCE_{it} > 0) = \alpha + \beta_1 \sum_{s=2}^{9} A_{it} + \beta_2 G_i + \beta_3 Y_i + \beta_4 \sum_{s=2}^{5} S_i + \beta_5 \sum_{u=2}^{8} U_i + \beta_6 \sum_{h=2}^{14} H_i$$

$$+ \beta_7 \sum_{c=2}^{3} C_{it} + \left(\beta_8 \sum_{c=2}^{3} C_{it} * \sum_{s=2}^{9} A_{it} \right) + u_i$$

Where: A is age at the time of admission (reference category: 50 -54 age group); G is sex (reference category: male); Y year of admission; S is SIMD quintile (reference category: most deprived quintile (1)); U is the urban/rural classification (reference category: large urban area);H is health board of inpatient admission (reference category: Greater Glasgow & Clyde); C is the Charlson comorbidity index (reference category: no comorbidities); u_i is the error term for patient *i* at time *t*.

For peer review only

Table II. Regression interactions: probability of healthcare resources utilisation and cost estimation (alive at the end of the five-year follow-up period)

Covariates	Probability (1st modelling part)		Probability (2nd modelling part)	
	Coefficient (95%CI)	Std. Err	Coefficient (95%CI)	Std. Err
**Age group (year)**				
50-54	Reference			
55-59	0.059 (-0.014, 0.131)	0.037	0.079 (0.024, 0.134)	0.028
60-64	0.164 (0.095, 0.234)	0.035	0.149 (0.099, 0.200)	0.026
65-69	0.208 (0.141, 0.275)	0.034	0.223 (0.173, 0.273)	0.025
70-74	0.241 (0.175, 0.307)	0.034	0.343 (0.295, 0.391)	0.024
75-79	0.323 (0.257, 0.389)	0.034	0.415 (0.368, 0.462)	0.024
80-84	0.210 (0.142, 0.277)	0.034	0.546 (0.497, 0.596)	0.025
85-89	0.133 (0.061, 0.205)	0.037	0.736 (0.682, 0.790)	0.028
90-max	-0.159 (-0.241, -0.077)	0.042	0.974 (0.908, 1.041)	0.034
**Sex**				
Male	Reference			
Female	0.050 (0.030, 0.070)	0.010	0.047 (0.034, 0.060)	0.007
**Date of admission**	0.176 (0.175, 0.178)	0.001	-0.058 (-0.059, -0.057)	0.001
**SIMD quintile**				
1	Reference			
2	0.026 (-0.005, 0.056)	0.015	-0.049 (-0.069, -0.029)	0.010
3	-0.013 (-0.045, 0.019)	0.016	-0.076 (-0.098, -0.055)	0.011
4	-0.035 (-0.068, -0.003)	0.017	-0.109 (-0.131, -0.087)	0.011
5	-0.034 (-0.067, -0.002)	0.017	-0.154 (-0.176, -0.133)	0.011
**Geography**				
Large urban	Reference			
Other urban	-0.157 (-0.186, -0.127)	0.015	-0.031 (-0.050, -0.012)	0.010
Accessible small towns	-0.184 (-0.223, -0.145)	0.020	-0.049 (-0.075, -0.023)	0.013
Accessible rural	-0.236 (-0.274, -0.199)	0.019	-0.061 (-0.087, -0.036)	0.013
Remote small towns	-0.160 (-0.220, -0.100)	0.031	-0.013 (-0.056, 0.029)	0.021
Remote rural	-0.333 (-0.386, -0.280)	0.027	-0.066 (-0.104, -0.028)	0.019
Very remote small towns	-0.425 (-0.515, -0.335)	0.046	-0.096 (-0.159, -0.033)	0.032
Very remote rural	-0.391 (-0.461, -0.321)	0.036	-0.084 (-0.136, -0.031)	0.027
**Health boards**				
Great Glasgow and Clyde	Reference			
Lothian	-0.051 (-0.087, -0.015)	0.018	-0.052 (-0.073, -0.030)	0.011
Lanarkshire	0.014 (-0.025, 0.053)	0.020	-0.077 (-0.101, -0.053)	0.012
Ayrshire and Arran	-0.396 (-0.438, -0.354)	0.021	-0.063 (-0.092, -0.033)	0.015
Grampian	0.032 (-0.011, 0.075)	0.022	-0.052 (-0.078, -0.025)	0.013
Tayside	-0.455 (-0.495, -0.416)	0.020	-0.096 (-0.123, -0.069)	0.014
Fife	-0.086 (-0.134, -0.037)	0.025	-0.024 (-0.057, 0.009)	0.017
Highland	-0.170 (-0.228, -0.112)	0.030	-0.039 (-0.078, 0.000)	0.020
Forth Valley	-0.518 (-0.566, -0.470)	0.024	-0.113 (-0.147, -0.079)	0.017
Dumfries and Galloway	-0.315 (-0.372, -0.258)	0.029	-0.172 (-0.212, -0.132)	0.020
Borders	-0.535 (-0.595, -0.475)	0.031	-0.108 (-0.154, -0.062)	0.023
Western Isles	-1.175 (-1.278, -1.072)	0.053	0.144 (0.060, 0.227)	0.043
Orkney	-0.409 (-0.554, -0.264)	0.074	0.002 (-0.117, 0.120)	0.060
Shetland	-0.608 (-0.748, -0.467)	0.072	-0.061 (-0.196, 0.074)	0.069
**Comorbidity**				
no comorbidities	Reference			
1 comorbidity	0.534 (0.418, 0.651)	0.059	0.421 (0.339, 0.503)	0.042
>1 comorbidities	0.787 (0.619, 0.956)	0.086	0.912 (0.828, 0.996)	0.043

For peer review only - http://bmjopen.bmj.com/site/about/guidelines.xhtml

Table III. Regression interactions: probability of healthcare resources utilisation and cost estimation (alive at the end of the five-year follow-up period)

Covariates	Probability (1st modelling part)		Probability (2nd modelling part)	
	Coefficient (95%CI)	Std. Err	Coefficient (95%CI)	Std. Err
**Interaction: age (year) - Charlson score (1 comorbidity)**				
50-54	Reference			
55-59	-0.227 (-0.368, -0.086)	0.072	-0.075 (-0.173, 0.024)	0.050
60-64	-0.312 (-0.448, -0.177)	0.069	-0.104 (-0.197, -0.010)	0.048
65-69	-0.359 (-0.489, -0.229)	0.066	-0.131 (-0.222, -0.040)	0.047
70-74	-0.387 (-0.515, -0.259)	0.065	-0.183 (-0.273, -0.094)	0.046
75-79	-0.464 (-0.591, -0.337)	0.065	-0.196 (-0.283, -0.108)	0.045
80-84	-0.510 (-0.637, -0.382)	0.065	-0.203 (-0.294, -0.112)	0.046
85-89	-0.559 (-0.691, -0.426)	0.067	-0.243 (-0.339, -0.147)	0.049
90-max	-0.657 (-0.800, -0.514)	0.073	-0.270 (-0.383, -0.157)	0.058
**Interaction: age (year) - Charlson score (>1 comorbidities)**				
50-54	Reference			
55-59	-0.123 (-0.320, 0.075)	0.101	-0.014 (-0.117, 0.089)	0.053
60-64	-0.145 (-0.334, 0.043)	0.096	-0.118 (-0.214, -0.022)	0.049
65-69	-0.202 (-0.383, -0.020)	0.093	-0.227 (-0.320, -0.133)	0.048
70-74	-0.336 (-0.514, -0.158)	0.091	-0.344 (-0.434, -0.254)	0.046
75-79	-0.444 (-0.621, -0.267)	0.090	-0.428 (-0.517, -0.339)	0.045
80-84	-0.563 (-0.740, -0.386)	0.090	-0.481 (-0.573, -0.390)	0.047
85-89	-0.700 (-0.880, -0.519)	0.092	-0.549 (-0.645, -0.452)	0.049
90-max	-0.823 (-1.013, -0.634)	0.097	-0.712 (-0.824, -0.600)	0.057

For peer review only - http://bmjopen.bmj.com/site/about/guidelines.xhtml

Figure I. Average annual costs per patient hospitalised with AF by sector. Cost components with confidence interval are presented for each age group (alive at the end of the five-year follow-up period)

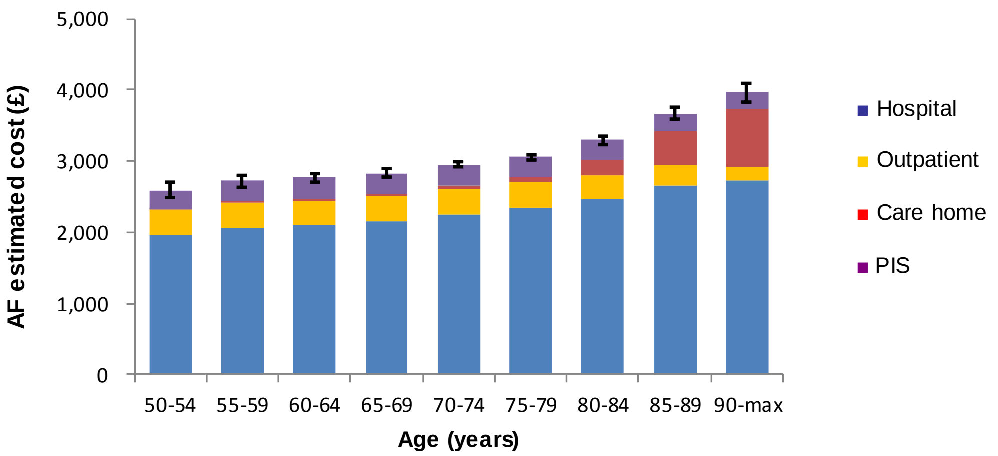

Age groups	Inpatient total cost (£) Mean (95%)	Outpatient total cost (£) Mean (95%)	Care home total cost (£) Mean (95%)	PIS total cost (£) Mean (95%)
(50-54)	1966 (1872, 2060)	348 (336, 361)	14 (9, 37)	264 (247, 282)
(55-59)	2064 (1994, 2134)	354 (345, 363)	17 (1, 36)	286 (274, 297)
(60-64)	2091 (2042, 2141)	354 (347, 361)	26 (14, 38)	294 (285, 304)
(65-69)	2155 (2111, 2198)	354 (349, 359)	33 (21, 44)	289 (283, 295)
(70-74)	2246 (2210, 2283)	354 (349, 358)	63 (49, 77)	290 (285, 295)
(75-79)	2343 (2311, 2375)	347 (343, 351)	84 (71, 97)	281 (277, 285)
(80-84)	2471 (2433, 2508)	315 (311, 319)	237 (213, 260)	268 (265, 272)
(85-89)	2661 (2614, 2708)	273 (268, 278)	481 (439, 522)	255 (251, 259)
(90-max)	2737 (2666, 2808)	188 (182, 194)	811 (735, 887)	227 (221, 233)
Total	2299 (2284, 2315)	324 (322, 326)	201 (190, 212)	270 (269, 273)

For peer review only - http://bmjopen.bmj.com/site/about/guidelines.xhtml

Equation V. Probability of healthcare utilisation (dead at the end of the five-year follow-up period)

$$\Pr(HCE_{it} > 0) = \alpha + \beta_1 \sum_{s=2}^{9} A_{it} + \beta_2 G_i + \beta_3 Y_i + \beta_4 \sum_{s=2}^{5} S_i + \beta_5 \sum_{u=2}^{8} U_i + \beta_6 \sum_{h=2}^{14} H_i$$

$$+ \beta_7 \sum_{c=2}^{3} C_{it} + \left(\beta_8 \sum_{c=2}^{3} C_{it} * \sum_{s=2}^{9} A_{it} \right)$$

Where: A is age at the time of admission (reference category: 50 -54 age group); G is sex (reference category: male); Y year of admission; S is SIMD quintile (reference category: most deprived quintile (1)); U is the urban/rural classification (reference category: large urban area);H is health board of inpatient admission (reference category: Greater Glasgow & Clyde); C is the Charlson comorbidity index (reference category: no comorbidities); u_i is the error term for patient *i* at time *t*.

Note: the model for care home does not include the 50-54 age group, as none of those patients incurred any cost related to care home.

For peer review only

Table IV. Regression interactions: probability of healthcare resources utilisation and cost estimation (dead at the end of the five-year follow-up period)

Covariates	Probability (1st modelling part) Coefficient (95%CI)	Std. Err	Probability (2nd modelling part) Coefficient (95%CI)	Std. Err
**Age group (year)**				
50-54	Reference			
55-59	0.013 (-0.229, 0.256)	0.124	0.153 (-0.119, 0.425)	0.139
60-64	0.008 (-0.207, 0.223)	0.110	0.246 (0.001, 0.491)	0.125
65-69	0.009 (-0.195, 0.213)	0.104	0.235 (-0.003, 0.474)	0.122
70-74	-0.014 (-0.212, 0.184)	0.101	0.263 (0.028, 0.497)	0.120
75-79	0.007 (-0.187, 0.201)	0.099	0.242 (0.009, 0.474)	0.119
80-84	0.011 (-0.182, 0.204)	0.098	0.302 (0.071, 0.533)	0.118
85-89	-0.170 (-0.362, 0.022)	0.098	0.313 (0.082, 0.543)	0.118
90-max	-0.640 (-0.831, -0.448)	0.098	0.344 (0.112, 0.576)	0.118
**Sex**				
Male	Reference			
Female	0.049 (0.033, 0.064)	0.008	0.027 (0.013, 0.042)	0.007
**Date of admission**	-0.040 (-0.042, -0.039)	0.001	0.004 (0.002, 0.005)	0.001
**SIMD quintile**				
1	Reference			
2	0.033 (0.011, 0.055)	0.011	0.015 (-0.005, 0.036)	0.011
3	0.059 (0.034, 0.083)	0.012	-0.007 (-0.030, 0.016)	0.012
4	0.067 (0.041, 0.092)	0.013	-0.017 (-0.042, 0.007)	0.013
5	0.116 (0.090, 0.141)	0.013	-0.026 (-0.050, -0.002)	0.012
**Geography**				
Large urban	Reference			
Other urban	-0.010 (-0.032, 0.012)	0.011	-0.034 (-0.055, -0.013)	0.011
Accessible small towns	-0.004 (-0.034, 0.026)	0.015	-0.051 (-0.080, -0.023)	0.014
Accessible rural	-0.032 (-0.062, -0.002)	0.015	-0.039 (-0.067, -0.011)	0.014
Remote small towns	-0.052 (-0.100, -0.003)	0.025	0.001 (-0.044, 0.047)	0.023
Remote rural	-0.038 (-0.084, 0.008)	0.024	-0.014 (-0.059, 0.032)	0.023
Very remote small towns	-0.066 (-0.148, 0.017)	0.042	0.034 (-0.054, 0.122)	0.045
Very remote rural	0.017 (-0.047, 0.082)	0.033	-0.005 (-0.072, 0.062)	0.034
**Health boards**				
Great Glasgow and Clyde	Reference			
Lothian	0.028 (0.003, 0.053)	0.013	0.030 (0.006, 0.053)	0.012
Lanarkshire	-0.053 (-0.081, -0.025)	0.014	-0.033 (-0.059, -0.006)	0.014
Ayrshire and Arran	-0.125 (-0.158, -0.092)	0.017	0.013 (-0.018, 0.045)	0.016
Grampian	0.075 (0.044, 0.106)	0.016	-0.058 (-0.087, -0.028)	0.015
Tayside	-0.025 (-0.057, 0.006)	0.016	-0.059 (-0.088, -0.031)	0.014
Fife	-0.026 (-0.062, 0.011)	0.018	0.045 (0.010, 0.080)	0.018
Highland	0.032 (-0.018, 0.081)	0.025	-0.062 (-0.115, -0.010)	0.027
Forth Valley	-0.060 (-0.099, -0.020)	0.020	-0.125 (-0.163, -0.087)	0.019
Dumfries and Galloway	-0.027 (-0.074, 0.020)	0.024	-0.014 (-0.058, 0.029)	0.022
Borders	-0.058 (-0.112, -0.005)	0.027	-0.022 (-0.073, 0.030)	0.026
Western Isles	-0.036 (-1.170, 1.098)	0.579	0.308 (-0.163, 0.778)	0.240
Orkney	0.188 (0.052, 0.324)	0.069	-0.177 (-0.315, -0.039)	0.070
Shetland	-0.040 (-0.181, 0.100)	0.072	-0.183 (-0.321, -0.046)	0.070
**Comorbidity**				
no comorbidities	Reference			
1 comorbidity	-0.169 (-0.442, 0.105)	0.140	0.288 (-0.003, 0.579)	0.148
>1 comorbidities	-0.208 (-0.434, 0.018)	0.115	0.788 (0.535, 1.041)	0.129

For peer review only - http://bmjopen.bmj.com/site/about/guidelines.xhtml

Table V. Regression interactions: probability of healthcare resources utilisation and cost estimation (dead at the end of the five-year follow-up period)

Covariates	Probability (1st modelling part)		Probability (2nd modelling part)	
	Coefficient (95%CI)	Std. Err	Coefficient (95%CI)	Std. Err
**Interaction: age (year) - Charlson score (1 comorbidity)**				
50-54	Reference			
55-59	0.123 (-0.221, 0.467)	0.175	-0.087 (-0.434, 0.260)	0.177
60-64	0.076 (-0.232, 0.383)	0.157	-0.149 (-0.465, 0.166)	0.161
65-69	0.021 (-0.271, 0.313)	0.149	-0.195 (-0.498, 0.109)	0.155
70-74	-0.014 (-0.299, 0.272)	0.145	-0.192 (-0.490, 0.107)	0.152
75-79	-0.086 (-0.366, 0.195)	0.143	-0.139 (-0.434, 0.157)	0.151
80-84	-0.207 (-0.486, 0.072)	0.142	-0.188 (-0.482, 0.106)	0.150
85-89	-0.167 (-0.445, 0.111)	0.142	-0.179 (-0.473, 0.114)	0.150
90-max	-0.162 (-0.440, 0.116)	0.142	-0.220 (-0.515, 0.075)	0.151
**Interaction: age (year) - Charlson score (>1 comorbidities)**				
50-54	Reference			
55-59	-0.195 (-0.482, 0.092)	0.147	-0.121 (-0.423, 0.182)	0.154
60-64	-0.183 (-0.438, 0.073)	0.130	-0.276 (-0.548, -0.004)	0.139
65-69	-0.263 (-0.506, -0.021)	0.124	-0.327 (-0.591, -0.062)	0.135
70-74	-0.239 (-0.476, -0.003)	0.121	-0.399 (-0.659, -0.139)	0.133
75-79	-0.359 (-0.592, -0.126)	0.119	-0.410 (-0.668, -0.153)	0.131
80-84	-0.491 (-0.722, -0.260)	0.118	-0.529 (-0.785, -0.273)	0.131
85-89	-0.441 (-0.672, -0.210)	0.118	-0.557 (-0.813, -0.301)	0.131
90-max	-0.360 (-0.590, -0.129)	0.118	-0.645 (-0.902, -0.388)	0.131

For peer review only - http://bmjopen.bmj.com/site/about/guidelines.xhtml

Figure II. Average annual costs per patient hospitalised with AF by sector. Cost components with confidence interval are presented for each age group (dead at the end of the five-year follow-up period)

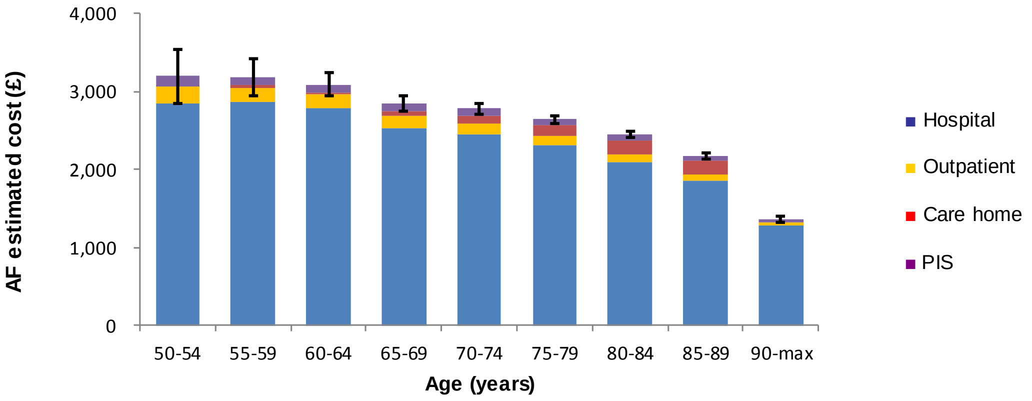

Age groups	Inpatient total cost (£) Mean (95%)	Outpatient total cost (£) Mean (95%)	Care home total cost (£) Mean (95%)	PIS total cost (£) Mean (95%)
(50-54)	2850 (2539, 3161)	205 (180, 230)	n/a	131 (99, 164)
(55-59)	2866 (2650, 3081)	181 (165, 197)	9 (5, 23)	115 (95, 135)
(60-64)	2795 (2661, 2929)	162 (153, 171)	28 (3, 53)	111 (99, 122)
(65-69)	2534 (2449, 2620)	153 (147, 160)	26 (12, 39)	95 (88, 102)
(70-74)	2449 (2386, 2511)	138 (134, 142)	60 (42, 78)	97 (92, 102)
(75-79)	2302 (2257, 2347)	118 (115, 121)	100 (80, 120)	83 (80, 86)
(80-84)	2099 (2062, 2136)	93 (91, 95)	139 (121, 157)	70 (67, 72)
(85-89)	1859 (1826, 1892)	69 (67, 71)	187 (167, 206)	58 (56, 60)
(90-max)	1289 (1263, 1316)	33 (31, 34)	180 (162, 199)	35 (34, 36)
Total	2012 (1996, 2029)	91 (90, 92)	142 (132, 152)	66 (64, 67)

Note: the care home total cost estimation does not include the 50-54 age group, as none of those patients incurred any cost related to care home.

For peer review only - http://bmjopen.bmj.com/site/about/guidelines.xhtml

STROBE 2007 (v4) Statement—Checklist of items that should be included in reports of *cohort studies*

Section/Topic	Item #	Recommendation	Reported on page #
**Title and abstract**	1	(*a*) Indicate the study's design with a commonly used term in the title or the abstract	1
		(*b*) Provide in the abstract an informative and balanced summary of what was done and what was found	1
**Introduction**			
Background/rationale	2	Explain the scientific background and rationale for the investigation being reported	3
Objectives	3	State specific objectives, including any prespecified hypotheses	3
**Methods**			
Study design	4	Present key elements of study design early in the paper	4
Setting	5	Describe the setting, locations, and relevant dates, including periods of recruitment, exposure, follow-up, and data collection	4
Participants	6	(*a*) Give the eligibility criteria, and the sources and methods of selection of participants. Describe methods of follow-up	4
		(*b*) For matched studies, give matching criteria and number of exposed and unexposed	n/a
Variables	7	Clearly define all outcomes, exposures, predictors, potential confounders, and effect modifiers. Give diagnostic criteria, if applicable	7-8
Data sources/ measurement	8*	For each variable of interest, give sources of data and details of methods of assessment (measurement). Describe comparability of assessment methods if there is more than one group	n/a
Bias	9	Describe any efforts to address potential sources of bias	7
Study size	10	Explain how the study size was arrived at	4
Quantitative variables	11	Explain how quantitative variables were handled in the analyses. If applicable, describe which groupings were chosen and why	7-8
Statistical methods	12	(*a*) Describe all statistical methods, including those used to control for confounding	6-8
		(*b*) Describe any methods used to examine subgroups and interactions	6-7
		(*c*) Explain how missing data were addressed	n/a
		(*d*) If applicable, explain how loss to follow-up was addressed	n/a
		(*e*) Describe any sensitivity analyses	n/a
**Results**			

For peer review only

Participants	13*	(a) Report numbers of individuals at each stage of study—eg numbers potentially eligible, examined for eligibility, confirmed eligible, included in the study, completing follow-up, and analysed	n/a
		(b) Give reasons for non-participation at each stage	n/a
		(c) Consider use of a flow diagram	n/a
Descriptive data	14*	(a) Give characteristics of study participants (eg demographic, clinical, social) and information on exposures and potential confounders	n/a
		(b) Indicate number of participants with missing data for each variable of interest	n/a
		(c) Summarise follow-up time (eg, average and total amount)	n/a
Outcome data	15*	Report numbers of outcome events or summary measures over time	n/a
Main results	16	(*a*) Give unadjusted estimates and, if applicable, confounder-adjusted estimates and their precision (eg, 95% confidence interval). Make clear which confounders were adjusted for and why they were included	9-11
		(*b*) Report category boundaries when continuous variables were categorized	n/a
		(*c*) If relevant, consider translating estimates of relative risk into absolute risk for a meaningful time period	n/a
Other analyses	17	Report other analyses done—eg analyses of subgroups and interactions, and sensitivity analyses	9
**Discussion**			
Key results	18	Summarise key results with reference to study objectives	11
**Limitations**			
Interpretation	20	Give a cautious overall interpretation of results considering objectives, limitations, multiplicity of analyses, results from similar studies, and other relevant evidence	12-14
Generalisability	21	Discuss the generalisability (external validity) of the study results	14
**Other information**			
Funding	22	Give the source of funding and the role of the funders for the present study and, if applicable, for the original study on which the present article is based	15

*Give information separately for cases and controls in case-control studies and, if applicable, for exposed and unexposed groups in cohort and cross-sectional studies.

Note: An Explanation and Elaboration article discusses each checklist item and gives methodological background and published examples of transparent reporting. The STROBE checklist is best used in conjunction with this article (freely available on the Web sites of PLoS Medicine at http://www.plosmedicine.org/, Annals of Internal Medicine at http://www.annals.org/, and Epidemiology at http://www.epidem.com/). Information on the STROBE Initiative is available at www.strobe-statement.org.

For peer review only

BMJ Open

Inpatient, Outpatient, Prescribing and Care Home Costs associated with Atrial Fibrillation

Journal:	*BMJ Open*
Manuscript ID	bmjopen-2018-028575.R2
Article Type:	Original research
Date Submitted by the Author:	15-Oct-2019
Complete List of Authors:	Ciminata, Giorgio; University of Glasgow Institute of Health and Wellbeing, Health Economics and Health Technology Assessment (HEHTA)<br>Geue, Claudia; University of Glasgow, Institute of Health and Wellbeing<br>Langhorne, Peter; University of Glasgow, Institute of Cardiovascular and Medical Sciences<br>Wu, O; University of Glasgow
**Primary Subject Heading**:	Health economics
Secondary Subject Heading:	Cardiovascular medicine
Keywords:	HEALTH ECONOMICS, Atrial Fibrillation, Stroke < NEUROLOGY, Cost Analysis

SCHOLARONE™
Manuscripts

I, the Submitting Author has the right to grant and does grant on behalf of all authors of the Work (as defined in the below author licence), an exclusive licence and/or a non-exclusive licence for contributions from authors who are: i) UK Crown employees; ii) where BMJ has agreed a CC-BY licence shall apply, and/or iii) in accordance with the terms applicable for US Federal Government officers or employees acting as part of their official duties; on a worldwide, perpetual, irrevocable, royalty-free basis to BMJ Publishing Group Ltd ("BMJ") its licensees and where the relevant Journal is co-owned by BMJ to the co-owners of the Journal, to publish the Work in this journal and any other BMJ products and to exploit all rights, as set out in our licence.

The Submitting Author accepts and understands that any supply made under these terms is made by BMJ to the Submitting Author unless you are acting as an employee on behalf of your employer or a postgraduate student of an affiliated institution which is paying any applicable article publishing charge ("APC") for Open Access articles. Where the Submitting Author wishes to make the Work available on an Open Access basis (and intends to pay the relevant APC), the terms of reuse of such Open Access shall be governed by a Creative Commons licence – details of these licences and which Creative Commons licence will apply to this Work are set out in our licence referred to above.

Other than as permitted in any relevant BMJ Author's Self Archiving Policies, I confirm this Work has not been accepted for publication elsewhere, is not being considered for publication elsewhere and does not duplicate material already published. I confirm all authors consent to publication of this Work and authorise the granting of this licence.

For peer review only

Inpatient, Outpatient, Prescribing and Care Home Costs associated with Atrial Fibrillation

Authors: Ciminata G (Research Assistant)[1], Geue C (Lecturer in Health Economics)[1], Langhorne P (Professor of Stroke Care)[2], Wu O (Professor of Health Technology Assessment)[1].

1. Health Economics and Health Technology Assessment (HEHTA), Institute of Health & Wellbeing, University of Glasgow, Glasgow G12 8RZ, UK
2. Institute of Cardiovascular & Medical Sciences, University of Glasgow, Glasgow G31 2ER, UK

Corresponding author:
Mr Giorgio Ciminata (MSc), g.ciminata.1@research.gla.ac.uk

Cover title: Inpatient, Outpatient, Prescribing and Care Home Costs associated with Atrial Fibrillation

Total number of tables and figures: Figures: 2, Tables: 5

Subject terms: Atrial Fibrillation

Key words: Atrial Fibrillation, Stroke, cost analysis

Word count: 3753

For peer review only

Abstract

Objective: To estimate global inpatient, outpatient, prescribing and care-home costs for atrial fibrillation (AF) patients, using population-based, individual-level linked data.

Design: A two-part model was employed to estimate the probability of resource utilisation and costs conditional on positive utilisation using individual-level linked data.

Settings: Scotland, five years following first hospitalisation for AF between 1997 and 2015.

Participants: Patients hospitalised with a known diagnosis of AF or atrial flutter.

Primary and secondary outcome measures: Inpatient, outpatient, prescribing and care home costs.

Results: The mean annual cost for an AF patient was estimated at £3785 (95% CI £3767-£3804). Inpatient admissions and outpatient visits accounted for 79% and 8% of total costs, respectively; prescriptions and care home stay accounted for 7% and 6% of total costs. Inpatient cost was the main driver across all age groups. While inpatient cost contributions (~80%) were constant between 0 and 84 years, they decreased for patients over 85 years. This is offset by increasing care-home cost contributions. Mean annual costs associated with AF increased significantly with increasing number of comorbidities.

Conclusion: This study used a contemporary and representative cohort, and a comprehensive approach to estimate global costs associated with AF, taking into account resource utilisation beyond hospital care. While overall costs, considerably affected by comorbidity, did not increase with increasing age, care-home costs increased proportionally with age. Inpatient admission was the main contributor to the overall financial burden of AF, highlighting the need for improved mechanisms of early diagnosis to prevent hospitalisations.

For peer review only

Article summary

Strengths and limitations of this study

- Costs are estimated through an incidence-based approach using patient-level morbidity records.
- Sufficient follow-up time is used to capture all relevant global costs to generate a contemporary estimate of health and care home costs related to AF.
- Scotland offers a robust record linkage system, where administrative patient-level health data are routinely collected.
- Data on primary care consultations were not available for linkage at a national level, however the impact this might have on overall costs is expected to be small.
- The potential risk of AF going undiagnosed and clinical miscoding of morbidity records may lead to an underestimation of the AF cohort and associated costs.

For peer review only

Introduction

Atrial Fibrillation (AF) is the most common form of arrhythmia. In Scotland AF affects 1.8% of the adult population, and rises to 6% among those aged 65 years or over [1]. In an ageing population, AF has a substantial impact on the economic burden of the healthcare system.

A number of cost analyses on estimating the economic burden of AF exist. The majority of these studies used various definition of the AF study population , based on data sourced from administrative database [2-4], health insurance databases [2, 5-7], hospital records [8, 9] and surveys [10]. Direct medical costs related to inpatient admissions, outpatient visits, as well as prescriptions have been included in these estimates; [2-10] indirect costs related to loss of productivity have been estimated among patients who were at working ages [6, 7].

There is a lack of generalisable studies based on large national population datasets that examine the total and the distribution of costs associated with AF [11]. The aim of this study was to quantify the inpatient, outpatient, prescribing and care home costs associated with AF over a five-year period. Using record-linkage of national datasets from Scotland, we also examined the distribution of costs that are attributable to AF.

Methods

Cost analyses or cost of illness studies typically adopt either the prevalence or incidence based approaches [12]. In the context of AF, the prevalence-based approach determines costs attributable to all cases of AF in a given year, while the incidence-based approach determines costs of new cases of AF in a given time period. In the present study, costs were estimated with an incidence-based approach. A further distinction between costing analyses is between the medicalized and the global comprehensive approaches.

For peer review only

In the first case, only expenditures directly attributable to a particular disease are used for estimating the overall costs. While the medicalized approach can be used to identify highly specific expenditures, it may also lead to underestimation or overestimation of the economic burden of a given disease; this may happen when cost estimation is not adequately adjusted for confounders highly correlated with the disease of interest. Conversely, the global comprehensive approach, used in this analysis, includes all the expenditures incurred by a population with a particular disease [13]. These expenditures are not necessarily related to the disease of interest; for instance, expenditures related to orthopaedics surgery or cancer treatment incurred by a patient with AF, will count towards the global comprehensive cost of AF.

Data

Data were obtained from the Information Services Division (ISD) of NHS Scotland as part of a wider project that used routinely collected data to evaluate clinical effectiveness and cost-effectiveness of Direct Oral Anticoagulants (DOACs) in the prevention of stroke in the AF population. Inpatient records for patients with a diagnosis of AF or atrial flutter between 1997 and 2015 were extracted from the General Acute Inpatient and Day Case Scottish Morbidity Records 01 (SMR01). These records contain all general acute admissions, categorized as inpatients or day cases, discharged from non-obstetric and non-psychiatric specialties [14]. Incident AF events (ICD10 code I48) were identified using all six diagnostic positions in SMR01, with a look back period of five years to minimise double counting. After checking for data entry errors and removal of duplicate records, the final AF cohort consisting of 278,286 individuals hospitalised with a diagnosis of AF or atrial flutter was identified.

For peer review only

Individual-level data linkage was then carried out with outpatient clinic attendance (Outpatient Attendance Scottish Morbidity Records 00; SMR00), the prescribing information system (PIS), care home census and mortality records (National Records for Scotland, NRS). Records from SMR00 include information on new and follow up outpatient appointments for any clinical specialty [15]. The PIS database includes prescribing records for all medicines and their associated costs, which are prescribed and dispensed by community pharmacies, dispensing doctors and a small number of specialist appliance suppliers [16]. The quality of PIS data is guaranteed by an electronic data capture, and it passes several stages of quality control before and after data are submitted [17]. The care home census combines the former Residential Care Home Census (run by the Scottish Government) and the Private Nursing Homes Census (run by ISD Scotland). Items reported in the care home census include discharge dates to care home residency such as NHS and private nursing homes, as well as an indication on whether nursing care is required [16].

Patients were followed up for five years following incident AF event in terms of their healthcare resource use, care home admissions and mortality. Since AF is often a precursor of stroke and cardiovascular conditions, an estimation of costs for a period of five years post AF event would allow us to fully capture costs associated with an AF patient.

Costing

Inpatient care costs were obtained from the latest (2013/2014) Scottish National Tariff (SNT), a list of standard average prices based on Healthcare Resource Groups (HRGs) [17, 18]. The SNT uses HRG4 for grouping clinically similar treatments that use similar levels of healthcare resources. After defining a total cost per episode, the total cost for a continuous inpatient stay (CIS) was calculated.

For peer review only

A CIS describes the entire duration of an inpatient stay from the date of admission to the date of discharge and can consist of several episodes in different specialties. Since the SNT is based on spells of care (inpatient stay within the same specialty) rather than individual inpatient episodes or a CIS, a CIS was partitioned into spells when a change in specialty occurred [17]. If within a CIS, two or more episodes were in the same specialty, only the highest incurred cost was taken into account, and the remaining episodes were replaced with a zero cost. Outpatient costs were obtained by assigning outpatient specialty costs, to outpatient attendances [17]. Unit costs were specific to whether the outpatient attendance took place at a consultant or nurse led clinic [15].

The cost of each prescription dispensed per patient was obtained from PIS [19]. Firstly, the price per unit was obtained by dividing the item price by the pack size. Secondly, the total number of items dispensed was obtained by multiplying the number of items dispensed by the number of instalments. Care home costs, obtained from the care home census, were based on length of stay or residency. Care home residency was established from care home census records, reporting admission to a care home like structure [16]. An average of care home charges for long stay residents was calculated using information on whether nursing care was provided or not. The average weekly care home charge was expressed per day, so that only the effective days spent in a care home were costed. The tariffs used for costing account for inflation, therefore further cost adjustment was not needed.

Econometric model

Healthcare expenditure data are typically characterised by: i) a significant proportion of zero-cost observations for individuals who have not utilised any healthcare resources in a given time period, and ii) a skewed distribution for positive costs. A two-part model was used [20, 21].

For peer review only

In the first part of the model, the probability of using a healthcare service in a given time period was estimated using a probit model (Equation I, please see online supplement). The same explanatory variables were used in the second part of the model, with a gamma distribution and log link, estimating costs conditional on having incurred positive costs (Equation II, please see online supplement). Mean costs per patient per year following their incident AF event were calculated by multiplying first and second modelling part (Equation III, please see online supplement).

In order to account for the skewed nature of cost data, generalised linear models (GLMs) were used. These were compared against ordinary least squares regression (OLS) and log transformed OLS by means of the Akaike Information Criterion (AIC), which measures goodness of fit. When comparing the different models, GLM reported the lowest AIC indicating the best fit for the given set of data. A user-written STATA programme "glmdiagnostic.do" [20], performing four different tests simultaneously, was used to identify the most appropriate distributional family and link function.

Econometric model covariates

The two-part model adjusted for age, sex, year of inpatient admission, socio-economic status, urban-rural classification, health board, comorbidities and mortality. These covariates are considered to be the main confounders that have an effect on costs incurred by an AF population. We controlled for age because AF and associated comorbidities are age-related conditions, and may have an impact on the overall costs. We also assumed costs to vary between males and females, in particular those for care home residency. Variation in healthcare utilisation and associated costs and care home residency by socio-economic status is controlled for using the Scottish Index of Multiple Deprivation (SIMD).

For peer review only

The SIMD reflects areas of multiple deprivation ranked from the most to the least deprived and expressed as quintiles where the most and the least deprived areas are represented by 1 and 5 respectively [22]. In Scotland, there are 14 regional health boards responsible for the provision of healthcare [23]. Hence, potential differences in healthcare utilisation and prescribing costs, may reflect variation in clinical practice and prescribing behaviour rather than the ability of patients to access care. Patients living in urban areas may have easier access to care compared to patients living in more remote areas, which is controlled for including the 8-fold classification measuring rurality [24].

Patients with one or more comorbidities are expected to incur significantly higher costs than those with none. We accounted for this by including the Charlson comorbidity index, where 1 indicates the absence of comorbidities, 2 the presence of only a single comorbidity and 3 the presence of more than one comorbidity [25]. Two interaction terms between age and comorbidities, and mortality and SIMD were included in the econometric model. Intuitively, a relationship of direct proportionality between age and comorbidities suggests that the level of comorbidities increases, as patients get older. Similarly, the socio-economic status may significantly influence the rate of socio-economic inequalities in mortality [26].

Sensitivity analyses

In order to ascertain whether mortality had an impact on overall AF related healthcare costs, average annual cost per patient by age and for each health or care home sector, was estimated for patients who were alive and those who were dead at the end of the five-year follow-up period. The two econometric models (Equation IV and V, please see online TABLEent) followed the same structure of the model described in the previous section and used for the main analysis; however, those models were not adjusted for mortality.

For peer review only

Ethics statement

The authors state that no ethical approval was needed.

Patients and public involvement

There was no patients or public involvement

Results

Cohort characteristics

Of the 278,286 AF patients with a mean age of 74 years (SD 12.5), the majority were identified in the two largest urban health board areas (Greater Glasgow & Clyde and Lothian), accounting for 22.2% and 14.8% respectively. This is also reflected in our categorisation of geographical areas, where large urban represented 38.4% and other urban areas represented 29.7% of the total AF cohort. Greater proportion of patients live in areas belonging to the most deprived quintile compared with those living in the least deprived areas – SIMD quintile 1 and quintile 5 representing 22.5% and 16.6% of the AF cohort respectively (Table1).

Econometric modelling results

Regression results for both modelling parts are presented in Table 2. Overall, an inversely U-shaped association between age and the likelihood of utilising any health or social care services was observed – a gradual increment in the likelihood in resource use with advancing age up to 80 years, when compared with the reference group (0-49 years), while patients 80 years or older showing a decreased probability of utilising healthcare services. However, this association was not observed in the second modelling part model, estimating costs conditional on having incurred positive costs, where a statistically significant gradient between age and costs indicated increasing costs as the cohort ages.

For peer review only

The use of health or social care services and associated costs also increased significantly for patients living in the most deprived areas, when compared with patients living in areas with the lowest level of deprivation. The effect of socio-economic status on healthcare utilisation was also measured for those who are alive at the end of the five-year follow-up period through an interaction term between SIMD and mortality, but no statistically significant effect was found. Full details of regression results for interaction terms are presented in the supplementary online material (Table I, please see online supplement).

For patients with comorbidities, the probabilities of utilising healthcare services were greater than the probability for those with no comorbidities. Although healthcare utilisation increased with the number of comorbidities, the interaction term between age and comorbidities indicated that as patients get older the use of healthcare services on average is lower for patients with one or more comorbidities than those with none. The decrease in healthcare utilisation by age is more pronounced in patients with more comorbidities than in those with only one comorbidity. The difference in healthcare costs between comorbidity categories indicated that in the presence of one or more comorbidities, on average healthcare costs decrease as patients get older. Full details of regression results for patients who were alive and those who were dead at the end of the five-year follow-up period are presented in Table 3 and Table 4 respectively, while regression results for interaction terms are presented in the supplementary online material (Table II and Table III, please see online supplement).

Cost estimates

The estimated mean annual cost per AF patient was £3785 (95% CI: £3767-£3804). The estimated total costs and distribution of costs according to sex are shown in Table 5.

For peer review only

While there is little difference between the total costs and the distribution of costs for inpatient, outpatient and prescription costs, the difference seems more pronounced when comparing the care home component of costs (5% of total costs among males vs 7% of total costs among females).

The average annual cost per AF patient by age and for each health or care home sector is shown in Figure 1. Considering the individual contribution of each cost component to the overall costs, inpatient costs was the main driver across all age groups. While inpatient cost contribution remained constant with an average contribution of about 80% to the overall costs for patients aged between 0 and 84 years, it decreased for patients over 85 years of age. Similar patterns were observed for outpatient and prescribing costs. On the contrary, the contribution of care home costs to the overall costs increased with age (0.5% for patients aged 0-49 years and approximately 11% for patients who are 90 years or older). The contribution of each setting to the total health and care home costs by the number of existing comorbidities is illustrated in Figure 2. While inpatient and total costs vary considerably with the number of comorbidities, outpatient and care home contributions remain fairly constant.

The estimated mean annual cost per AF patient alive at the end of the five-year follow-up period was £3047 (95% CI: £3027-£3067). The average annual cost per AF patient by age and for each health or care home sector is presented in the supplementary online material (Figure I, please see online supplement). For these patients, inpatient cost was the main driver across all age groups; a gradient between age and costs indicated increasing costs as the cohort ages. Similar patterns were observed for care home costs. On the contrary, outpatient and prescribing costs remained constant up to 74 years, but decreased slightly for older patients.

For peer review only

The estimated mean annual cost per AF patient who died during the five-year follow-up period, was £2304 (95% CI: £2284-£2324) (Figure II, please see online supplement). For these patients, inpatient cost was the main driver across all age groups; a gradient between age and costs indicated decreasing costs as the cohort ages. This was also observed for outpatient and prescribing costs; but care home costs on average increased across age groups.

Discussion

A greater proportion of AF patients were found in areas with the highest index of deprivation. This, combined with the likelihood for people living in the most deprived quintile having longer inpatient stays due to a lack of support at home, may explain the difference in inpatient care utilisation between patients from the most and the least deprived areas, with associated costs being higher for the former group. As AF is more likely to affect the elderly, so that costs were expected to increase with age. As health deteriorates with age, older age groups are assumed to make greater use of healthcare services, and therefore incur higher costs than younger age groups. However, age was found to have a modest impact on overall healthcare costs, being fairly consistent across age groups. This finding is in line with existing evidence indicating that healthcare expenditure depends not only on patients' calendar age, but is also significantly associated with remaining lifetime [27].

Any observed correlation between healthcare expenditure and age may therefore be attributable to the fact that the proportion of patients who are at the end of their lives is substantially greater in older rather than younger age groups [27]. On the other hand, comorbidity had a considerable effect on the overall costs, increasing significantly in patients with more than one comorbidity. However, the decrease in healthcare utilisation by age is more pronounced in patients with more comorbidities than in those with only one comorbidity.

For peer review only

Decreasing inpatient and outpatient costs for the oldest patients were offset by increasing care home costs, in particular for women. Indeed, the main cause for higher overall costs incurred by women is attributable to the higher likelihood for elderly women to reside in care homes. Interestingly, care home contribution to the overall costs was noticeably lower for patients with multiple comorbidities than for those with none or one comorbidity. This may suggest that sicker patients are more likely to be in hospital than in a care home.

To date, only one single study published in 2004 has estimated the cost of AF in Scotland; the authors estimated the cost of AF in 1995/1996 with the medicalised approach , and projected these to the year 2000 [28]. Previous work has focussed on a 12-months follow-up, which seems limited in order to capture all healthcare resource utilisation for AF patients. Our study offers a longer follow-up and a contemporary estimate of healthcare costs related to AF including all relevant care settings. Our study offers a distinct advantage over previous work as costs, rather than being based on extrapolated rates using a prevalence-based approach [28], are estimated with an incidence-based method using patient-level morbidity records. Using an incidence based approach to costing and a broad perspective to capture the majority of costs associated with AF, several routinely collected administrative datasets from Scotland were combined, including care home utilisation.

Existing studies, including ours, regardless of econometric model choice and covariates used, show that costs due to inpatient admission are the main contributor to overall AF related healthcare cost. This is a pertinent finding that may well support future policies on opportunistic screening in the population at risk of AF, and in particular in Scotland where 1 in 3 patients with AF are currently undiagnosed [29].

For peer review only

The European AF management guidelines and the Scottish Cross-Party Group 'Heart Disease and Stroke', recently recommended that people who are 65 years or older and at risk of AF and associated comorbidities such as cardiovascular disease, diabetes or respiratory disease should be screened opportunistically in primary care, pharmacies or community settings [29, 30]. With rigorous screening and appropriate treatment, hospitalisations could be avoided and costs reduced.

Although we have captured most healthcare sectors and related costs, we were not able to obtain national data on primary care consultations, as these data are currently not routinely available for linkage in Scotland. Not capturing these data, may lead to an underestimation of the size of the AF cohort and associated costs. However, the costs associated with primary care consultations is expected to have a limited impact on the overall total AF related costs. Such underestimation could also result from AF going undiagnosed and clinical miscoding of morbidity records. Nevertheless, by using a cohort of patients hospitalised with AF we were able to capture more severe cases of AF. Prescribing and care home data were only available respectively from 2009 to 2012, their contribution to overall AF related costs might also be underestimated. Other limitations are inherent to the nature of administrative data, such as missing records or incomplete data.

Further, we acknowledge the issue concerning attributing AF related costs to patients with a structural heart disease, as AF may manifest subsequently because of this. In our analysis, we identified about 14% of AF patients with a structural heart disease; these were patients with systolic dysfunction, valvular heart disease or heart valve replacement. However, from the hospital data it was not possible to establish causation between structural heart disease and AF.

For peer review only

In addition, this is likely to have a marginal impact on our conclusions, as the global comprehensive approach used in this study include expenditures that are not necessarily related to AF.

We also acknowledge that specifying whether patients had received cardiovascular procedures (e.g. cardioversion, echocardiograms and angiograms) would improve the accuracy of our cost estimation, as it would indicate whether costs should be attributable to AF or coronary artery disease. However, this information is not currently available in our routinely collected data of hospital admissions. Nevertheless, because AF is coded at discharge, we can be confident that the estimated costs are attributable to AF.

Recognising these limitations, we were nevertheless able to harness high quality patient-level linked data to identify a cohort of AF patients and to estimate their associated healthcare utilisation and costs in Scotland.

The inclusion of all available cost components is crucial for establishing overall costs, as these often extend beyond hospitalisation. The study identifies hospitalisation as the main cost driver and suggests that the implementation of AF screening policies could substantially reduce AF related health care costs. Most importantly, the study concludes that patient's age has a limited impact on the overall AF related cost, and therefore may contribute much less to future growth of AF related cost in an ever-ageing Scottish population.

Future work will be able to utilise Scottish Stroke Care Audit (SSCA) records, allowing for the identification of additional AF patients; these are patients hospitalised with a stroke, where AF has been recorded in audit data as an underlying comorbidity.

For peer review only

Being able to complement inpatient records with SSCA records will allow us to capture more AF patients in Scotland. Moreover, future research may be able to include indirect costs associated with productivity-loss by linking morbidity and prescribing data to national data from the Department for Work and Pensions, for instance.

For peer review only

Acknowledgements: We thank the members of the Information Services Division, National Services Scotland, for their support. We acknowledge the support from The Farr Institute @ Scotland. The Farr Institute @ Scotland is supported by a 10-funder consortium: Arthritis Research UK, the British Heart Foundation, Cancer Research UK, the Economic and Social Research Council, the Engineering and Physical Sciences Research Council, the Medical Research Council, the National Institute of Health Research, the National Institute for Social Care and Health Research (Welsh Assembly Government), the Chief Scientist Office (Scottish Government Health Directorates), the Wellcome Trust (MRC Grant No: MR/K007017/1).

Author contributions: GC, CG, PL and OW conceived the article. GC carried out the statistical analysis and prepared the first draft of the manuscript. GC, CG, PL and OW contributed to editing the manuscript and approved the final version submitted for publication.

Funding sources: This work was supported by Farr Institute for Health Informatics Research, Scotland, MRC, grant number MR/K007017/1

Competing interest: The authors declare no conflict of interest.

Data Statement: All data underlying the analyses are confidential and subject to disclosure control. Data can only be obtained through application to Information Services Division (ISD) via the Public Benefit and Privacy Panel (PBPP).

For peer review only

References

1. Heath Improvement Scotland. *Prevention of stroke in patients with atrial fibrillation.* 2013 [cited 2016 January 2016]; Available from: http://www.sign.ac.uk/assets/af_publication.pdf.
2. Bennell, M.C., et al., *Identifying predictors of cumulative healthcare costs in incident atrial fibrillation: a population-based study.* J Am Heart Assoc, 2015. **4**(4).
3. Hallinen, T., et al., *Direct costs of warfarin treatment among patients with atrial fibrillation in a Finnish health care setting.* Curr Med Res Opin, 2006. **22**(4): p. 683-92.
4. Nelson, W.W., et al., *Out-of-range international normalized ratio values and healthcare cost among new warfarin patients with non-valvular atrial fibrillation.* J Med Econ, 2015. **18**(5): p. 333-40.
5. Ghate, S.R., et al., *All-cause and bleeding-related health care costs in warfarin-treated patients with atrial fibrillation.* J Manag Care Pharm, 2011. **17**(9): p. 672-84.
6. Rohrbacker, N.J., et al., *The burden of atrial fibrillation and other cardiac arrhythmias in an employed population: associated costs, absences, and objective productivity loss.* J Occup Environ Med, 2010. **52**(4): p. 383-91.
7. Wu, E.Q., et al., *Economic burden and co-morbidities of atrial fibrillation in a privately insured population.* Curr Med Res Opin, 2005. **21**(10): p. 1693-9.
8. Holstenson, E., et al., *Predictors of costs related to cardiovascular disease among patients with atrial fibrillation in five European countries.* Europace, 2011. **13**(1): p. 23-30.
9. Reynolds, M.R., et al., *Healthcare resource utilization and costs associated with recurrent episodes of atrial fibrillation: the FRACTAL registry.* J Cardiovasc Electrophysiol, 2007. **18**(6): p. 628-33.
10. Jonsson, L., et al., *Cost of illness and drivers of cost in atrial fibrillation in Sweden and Germany.* Appl Health Econ Health Policy, 2010. **8**(5): p. 317-25.
11. Johnsen, S.P., et al., *Cost of illness of atrial fibrillation: a nationwide study of societal impact.* BMC Health Services Research, 2017. **17**(1): p. 714.
12. Tarricone, R., *Cost-of-illness analysis. What room in health economics?* Health Policy, 2006. **77**(1): p. 51-63.
13. de Lagasnerie, G., et al., *The economic burden of diabetes to French national health insurance: a new cost-of-illness method based on a combined medicalized and incremental approach.* Eur J Health Econ, 2018. **19**(2): p. 189-201.
14. Information Services Division Scotland. *General Acute Inpatient and Day Case - Scottish Morbidity Record (SMR01).* 2015 [cited 2017 November 2017]; Available from: http://www.ndc.scot.nhs.uk/National-Datasets/data.asp?SubID=2.
15. Information Services Division Scotland. *Outpatient Attendance (SMR00).* 2015 [cited 2015 January 2015]; Available from: http://www.ndc.scot.nhs.uk/National-Datasets/data.asp?SubID=2.
16. Information Service Division Scotland. *Care Home Census 2015.* 2015 [cited 2015 November 2015]; Available from: http://www.isdscotland.org/Health-Topics/Health-and-Social-Community-Care/Care-Homes/Previous-Publications/.
17. Information Service Division Scotland. *Scottish National Tariff.* 2015 [cited 2015 December 2015]; Available from: http://www.isdscotland.org/Health-Topics/Finance/Scottish-National-Tariff/.

For peer review only

18. Healthcare Resource Groups. *Healthcare Resource Groups*. 2015 [cited 2015 November 2015]; Available from: http://www.hscic.gov.uk/hrg.
19. Alvarez-Madrazo, S., et al., *Data Resource Profile: The Scottish National Prescribing Information System (PIS).* International Journal of Epidemiology, 2016.
20. Henry, A.G., et al., *Economic Evaluation in Clinical Trials*. 'Oxford University Press': Oxford, UK.
21. Manning, W.G., A. Basu, and J. Mullahy, *Generalized modeling approaches to risk adjustment of skewed outcomes data.* Journal of Health Economics, 2005. **24**(3): p. 465-488.
22. The Scottish Government. *Scottish Index of Multiple Deprivation*. 2015 [cited 2015 November 2015]; Available from: http://www.gov.scot/Topics/Statistics/SIMD.
23. The Scottish Government. *NHS Boards*. 2015 [cited 2015 January 2015]; Available from: http://www.gov.scot/Topics/Health/NHS-Workforce/NHS-Boards.
24. The Scottish Government. *Scottish Government Urban Rural Classification*. 2015 [cited 2015 January 2015]; Available from: http://www.gov.scot/Topics/Statistics/About/Methodology/UrbanRuralClassification.
25. Charlson, M.E., et al., *A new method of classifying prognostic comorbidity in longitudinal studies: Development and validation.* Journal of Chronic Diseases, 1987. **40**(5): p. 373-383.
26. Leyland, A.H., et al., *Cause-specific inequalities in mortality in Scotland: two decades of change. A population-based study.* BMC Public Health, 2007. **7**: p. 172.
27. Zweifel, P., S. Felder, and M. Meiers, *Ageing of population and health care expenditure: a red herring?* Health Econ, 1999. **8**(6): p. 485-96.
28. Stewart, S., et al., *Cost of an emerging epidemic: an economic analysis of atrial fibrillation in the UK.* Heart, 2004. **90**(3): p. 286-292.
29. Stroke, C.-P.G.o.H.D.a. *A Focus on Atrial Fibrillation in Scotland 2017*. 2017 March 2018]; Available from: https://www.chss.org.uk/documents/2018/01/a-focus-on-atrial-fibrillation-in-scotland-pdf.pdf.
30. Camm, A.J., et al., *2012 focused update of the ESC Guidelines for the management of atrial fibrillation: an update of the 2010 ESC Guidelines for the management of atrial fibrillation. Developed with the special contribution of the European Heart Rhythm Association.* Eur Heart J, 2012. **33**(21): p. 2719-47.

For peer review only

Figure legends

Figure 1. Average annual costs per patient hospitalised with AF by sector. Cost components with confidence interval are presented for each age group.

Figure 2. Average cost per patient hospitalised with AF by Charlson Comorbidity Index. Cost components with confidence interval are presented for each Comorbidity category.

For peer review only

Tables

Table 1. Baseline characteristics of AF patients

Characteristics	N (%)
Number of patients	278,286
Mean age at first admission*(SD)**(range)	74 *(12.5) **(0 -108)
Sex	
Male	139,928 (50.3)
Female	138,358 (49.7)
Health Boards	
Greater Glasgow & Clyde	61,822 (22.2)
Lothian	41,169 (14.8)
Lanarkshire	31,049 (11.2)
Grampian	25,728 (9.3)
Ayrshire & Arran	22,003 (7.9)
Tayside	25,003 (9.0)
Fife	17,954 (6.5)
Highland	18,929 (6.9)
Forth valley	13,664 (4.9)
Dumfries & Galloway	9,798 (3.5)
Borders	7,222 (2.6)
Western isles	1,868 (0.7)
Shetland	1,036 (0.4)
Orkney	1,041 (0.4)
Geography	
Large/urban	106,868 (38.4)
Other/urban	82,601 (29.7)
Accessible small towns	24,938 (9.0)
Remote small towns	8,272 (3.0)
Very remote small towns	3,828 (1.4)
Accessible rural	30,826 (11.1)
Remote rural	10,371 (3.7)
Very remote rural	10,087 (3.6)
SIMD quintile	
1	62,730 (22.5)
2	62,632 (22.5)
3	55,943 (20.1)
4	50,691 (18.2)
5	46,279 (16.6)
Comorbidity	
no comorbidity	40,502 (14.6)
1 comorbidity	53,651 (19.3)
>1 comorbidities	184,133 (66.2)
Re-hospitalised (any condition)	179,494 (64.5)
Admitted to care-home	7,235 (2.6)
Mortality	
Alive	204,690 (73.6)
Dead	73,596 (26.4)

Table 2. Regression results: probability of healthcare resources utilisation and cost estimation

Covariates	Probability (1st modelling part)		Cost Ratios (2nd modelling part)	
	Coefficient (95% CI)	Std. Err	Coefficient (95% CI)	Std. Err
Age group (years)				
0-49	Reference			
50-54	0.329 (0.260, 0.398)	0.035	0.036 (-0.016, 0.087)	0.026
55-59	0.388 (0.326, 0.450)	0.031	0.081 (0.036, 0.127)	0.023
60-64	0.464 (0.407, 0.521)	0.029	0.124 (0.082, 0.166)	0.021
65-69	0.486 (0.432, 0.540)	0.028	0.157 (0.116, 0.198)	0.021
70-74	0.479 (0.426, 0.533)	0.027	0.213 (0.174, 0.252)	0.020
75-79	0.536 (0.482, 0.590)	0.027	0.222 (0.183, 0.260)	0.020
80-84	0.431 (0.375, 0.486)	0.028	0.286 (0.246, 0.326)	0.020
85-89	0.378 (0.318, 0.437)	0.030	0.375 (0.332, 0.417)	0.021
90-max	0.150 (0.083, 0.217)	0.034	0.516 (0.468, 0.564)	0.025
Sex				
Male	Reference			
Female	0.045 (0.028, 0.062)	0.009	0.054 (0.044, 0.064)	0.005
Date of admission	0.169 (0.167, 0.171)	0.001	-0.024 (-0.025, -0.023)	0.001
SIMD quintile				
1	Reference			
2	0.027 (-0.018, 0.071)	0.023	-0.055 (-0.080, -0.031)	0.012
3	-0.041 (-0.086, 0.003)	0.023	-0.080 (-0.106, -0.054)	0.013
4	-0.046 (-0.091, -0.002)	0.023	-0.116 (-0.141, -0.090)	0.013
5	-0.072 (-0.117, -0.027)	0.023	-0.147 (-0.172, -0.122)	0.013
Geography				
Large urban	Reference			
Other urban	-0.130 (-0.156, -0.105)	0.013	-0.023 (-0.037, -0.009)	0.007
Accessible small towns	-0.153 (-0.187, -0.119)	0.017	-0.041 (-0.060, -0.022)	0.010
Accessible rural	-0.197 (-0.230, -0.165)	0.016	-0.043 (-0.062, -0.024)	0.010
Remote small towns	-0.145 (-0.197, -0.093)	0.027	0.009 (-0.023, 0.041)	0.016
Remote rural	-0.288 (-0.335, -0.241)	0.024	-0.036 (-0.065, -0.007)	0.015
Very remote small towns	-0.380 (-0.459, -0.300)	0.041	-0.057 (-0.107, -0.006)	0.026
Very remote rural	-0.346 (-0.407, -0.284)	0.031	-0.061 (-0.102, -0.020)	0.021
Health boards				
Great Glasgow and Clyde	Reference			
Lothian	-0.044 (-0.075, -0.014)	0.016	-0.033 (-0.049, -0.017)	0.008
Lanarkshire	-0.005 (-0.038, 0.029)	0.017	-0.063 (-0.081, -0.045)	0.009
Ayrshire and Arran	-0.358 (-0.394, -0.321)	0.019	-0.046 (-0.068, -0.024)	0.011
Grampian	0.017 (-0.019, 0.054)	0.019	-0.059 (-0.078, -0.039)	0.010
Tayside	-0.402 (-0.436, -0.368)	0.018	-0.083 (-0.103, -0.062)	0.010
Fife	-0.059 (-0.101, -0.017)	0.022	-0.009 (-0.033, 0.016)	0.012
Highland	-0.175 (-0.225, -0.124)	0.026	-0.046 (-0.077, -0.015)	0.016
Forth Valley	-0.477 (-0.518, -0.436)	0.021	-0.109 (-0.135, -0.082)	0.013
Dumfries and Galloway	-0.303 (-0.352, -0.253)	0.025	-0.134 (-0.164, -0.104)	0.015
Borders	-0.501 (-0.554, -0.449)	0.027	-0.086 (-0.120, -0.052)	0.017
Western Isles	-1.072 (-1.171, -0.974)	0.050	0.457 (0.381, 0.533)	0.039

Orkney	-0.362 (-0.492, -0.232)	0.066	-0.029 (-0.117, 0.059)	0.045
Shetland	-0.495 (-0.622, -0.368)	0.065	-0.076 (-0.171, 0.018)	0.048
Mortality within 5 years				
Alive	Reference			
Dead	0.418 (0.376, 0.461)	0.022	0.652 (0.630, 0.674)	0.011
Comorbidity				
no comorbidities	Reference			
1 comorbidity	0.666 (0.567, 0.766)	0.051	0.374 (0.299, 0.450)	0.038
>1 comorbidities	1.205 (1.021, 1.390)	0.094	0.990 (0.910, 1.070)	0.041

Table 3. Regression results: probability of healthcare resources utilisation and cost estimation (alive at the end of the five-year follow-up period)

Covariates	Probability (1st modelling part)		Cost Ratios (2nd modelling part)	
	Coefficient (95% CI)	Std. Err	Coefficient (95% CI)	Std. Err
Age group (years)				
0-49	Reference			
50-54	0.352 (0.282, 0.422)	0.036	0.067 (0.013, 0.120)	0.027
55-59	0.424 (0.361, 0.488)	0.032	0.148 (0.098, 0.199)	0.026
60-64	0.528 (0.470, 0.586)	0.030	0.218 (0.174, 0.263)	0.023
65-69	0.571 (0.516, 0.627)	0.028	0.292 (0.248, 0.336)	0.022
70-74	0.603 (0.549, 0.658)	0.028	0.412 (0.371, 0.454)	0.021
75-79	0.684 (0.630, 0.739)	0.028	0.484 (0.443, 0.525)	0.021
80-84	0.572 (0.516, 0.628)	0.028	0.615 (0.572, 0.659)	0.022
85-89	0.496 (0.435, 0.557)	0.031	0.805 (0.756, 0.854)	0.025
90-max	0.206 (0.134, 0.279)	0.037	1.044 (0.981, 1.106)	0.032
Sex				
Male	Reference			
Female	0.067 (0.048, 0.086)	0.010	0.050 (0.037, 0.063)	0.007
Date of admission	0.171 (0.170, 0.173)	0.001	-0.059 (-0.060, -0.057)	0.001
SIMD quintile				
1	Reference			
2	0.021 (-0.009, 0.050)	0.015	-0.052 (-0.071, -0.033)	0.010
3	-0.023 (-0.054, 0.008)	0.016	-0.081 (-0.101, -0.060)	0.011
4	-0.045 (-0.077, -0.014)	0.016	-0.117 (-0.138, -0.096)	0.011
5	-0.051 (-0.083, -0.020)	0.016	-0.160 (-0.181, -0.139)	0.011
Geography				
Large urban	Reference			
Other urban	-0.140 (-0.169, -0.112)	0.014	-0.030 (-0.049, -0.012)	0.010
Accessible small towns	-0.172 (-0.210, -0.134)	0.019	-0.052 (-0.077, -0.026)	0.013
Accessible rural	-0.217 (-0.253, -0.181)	0.018	-0.061 (-0.086, -0.037)	0.013
Remote small towns	-0.145 (-0.203, -0.087)	0.030	-0.007 (-0.048, 0.035)	0.021
Remote rural	-0.319 (-0.371, -0.268)	0.026	-0.064 (-0.101, -0.027)	0.019
Very remote small towns	-0.404 (-0.491, -0.318)	0.044	-0.098 (-0.161, -0.036)	0.032
Very remote rural	-0.360 (-0.428, -0.293)	0.034	-0.087 (-0.138, -0.035)	0.026

Health boards				
Great Glasgow and Clyde	Reference			
Lothian	-0.055 (-0.090, -0.020)	0.018	-0.051 (-0.072, -0.030)	0.011
Lanarkshire	0.003 (-0.034, 0.040)	0.019	-0.072 (-0.095, -0.048)	0.012
Ayrshire and Arran	-0.396 (-0.436, -0.355)	0.021	-0.064 (-0.093, -0.035)	0.015
Grampian	0.029 (-0.013, 0.070)	0.021	-0.051 (-0.077, -0.026)	0.013
Tayside	-0.453 (-0.491, -0.415)	0.019	-0.094 (-0.120, -0.067)	0.014
Fife	-0.087 (-0.134, -0.040)	0.024	-0.024 (-0.057, 0.008)	0.017
Highland	-0.191 (-0.247, -0.135)	0.029	-0.037 (-0.075, 0.001)	0.020
Forth Valley	-0.520 (-0.566, -0.474)	0.023	-0.108 (-0.141, -0.074)	0.017
Dumfries and Galloway	-0.314 (-0.369, -0.259)	0.028	-0.166 (-0.206, -0.127)	0.020
Borders	-0.547 (-0.605, -0.489)	0.030	-0.099 (-0.144, -0.054)	0.023
Western Isles	-1.164 (-1.264, -1.063)	0.051	0.139 (0.057, 0.221)	0.042
Orkney	-0.394 (-0.535, -0.252)	0.072	0.002 (-0.114, 0.117)	0.059
Shetland	-0.605 (-0.740, -0.470)	0.069	-0.044 (-0.172, 0.085)	0.066
Comorbidity				
no comorbidities	Reference			
1 comorbidity	0.705 (0.602, 0.808)	0.052	0.432 (0.352, 0.513)	0.041
>1 comorbidities	1.165 (0.974, 1.357)	0.098	1.133 (1.041, 1.226)	0.047

Table 4. Regression results: probability of healthcare resources utilisation and cost estimation (dead at the end of the five-year follow-up period)

Covariates	Probability (1st modelling part)		Cost Ratios (2nd modelling part)	
	Coefficient (95% CI)	Std. Err	Coefficient (95% CI)	Std. Err
Age group (years)				
0-49	Reference			
50-54	0.150 (-0.125, 0.426)	0.141	-0.112 (-0.405, 0.180)	0.149
55-59	0.134 (-0.098, 0.366)	0.118	-0.093 (-0.334, 0.147)	0.123
60-64	0.129 (-0.080, 0.338)	0.107	0.000 (-0.208, 0.209)	0.106
65-69	0.129 (-0.067, 0.326)	0.101	-0.011 (-0.212, 0.189)	0.102
70-74	0.107 (-0.084, 0.298)	0.097	0.016 (-0.180, 0.213)	0.100
75-79	0.128 (-0.059, 0.315)	0.095	-0.005 (-0.198, 0.189)	0.099
80-84	0.132 (-0.053, 0.318)	0.095	0.056 (-0.136, 0.247)	0.098
85-89	-0.048 (-0.233, 0.137)	0.094	0.066 (-0.126, 0.257)	0.098
90-max	-0.518 (-0.702, -0.333)	0.094	0.097 (-0.095, 0.290)	0.098
Sex				
Male	Reference			
Female	0.048 (0.033, 0.063)	0.008	0.028 (0.014, 0.043)	0.007
Date of admission	-0.040 (-0.042, -0.039)	0.001	0.004 (0.002, 0.005)	0.001
SIMD quintile				
1	Reference			
2	0.033 (0.011, 0.055)	0.011	0.015 (-0.005, 0.036)	0.011
3	0.058 (0.034, 0.082)	0.012	-0.008 (-0.030, 0.015)	0.012
4	0.065 (0.039, 0.090)	0.013	-0.017 (-0.041, 0.007)	0.012

5	0.113 (0.088, 0.138)	0.013	-0.024 (-0.049, 0.000)	0.012
Geography				
Large urban	Reference			
Other urban	-0.010 (-0.032, 0.012)	0.011	-0.033 (-0.054, -0.012)	0.011
Accessible small towns	-0.006 (-0.036, 0.025)	0.015	-0.049 (-0.077, -0.021)	0.014
Accessible rural	-0.031 (-0.060, -0.001)	0.015	-0.036 (-0.064, -0.008)	0.014
Remote small towns	-0.054 (-0.102, -0.005)	0.025	0.003 (-0.042, 0.049)	0.023
Remote rural	-0.038 (-0.084, 0.009)	0.024	-0.012 (-0.057, 0.034)	0.023
Very remote small towns	-0.065 (-0.147, 0.017)	0.042	0.036 (-0.052, 0.123)	0.045
Very remote rural	0.014 (-0.051, 0.078)	0.033	-0.002 (-0.068, 0.065)	0.034
Health boards				
Great Glasgow and Clyde	Reference			
Lothian	0.029 (0.004, 0.055)	0.013	0.029 (0.006, 0.053)	0.012
Lanarkshire	-0.052 (-0.080, -0.023)	0.014	-0.034 (-0.061, -0.008)	0.013
Ayrshire and Arran	-0.122 (-0.155, -0.089)	0.017	0.011 (-0.020, 0.042)	0.016
Grampian	0.075 (0.044, 0.106)	0.016	-0.057 (-0.086, -0.028)	0.015
Tayside	-0.024 (-0.056, 0.007)	0.016	-0.061 (-0.089, -0.033)	0.014
Fife	-0.028 (-0.064, 0.008)	0.018	0.047 (0.012, 0.082)	0.018
Highland	0.034 (-0.015, 0.084)	0.025	-0.065 (-0.117, -0.013)	0.027
Forth Valley	-0.060 (-0.099, -0.021)	0.020	-0.123 (-0.161, -0.085)	0.019
Dumfries and Galloway	-0.027 (-0.074, 0.020)	0.024	-0.014 (-0.058, 0.029)	0.022
Borders	-0.058 (-0.112, -0.005)	0.027	-0.023 (-0.074, 0.029)	0.026
Western Isles	-0.033 (-1.168, 1.102)	0.579	0.305 (-0.165, 0.775)	0.240
Orkney	0.191 (0.055, 0.327)	0.069	-0.180 (-0.317, -0.042)	0.070
Shetland	-0.031 (-0.170, 0.108)	0.071	-0.187 (-0.323, -0.052)	0.069
Comorbidity				
no comorbidities	Reference			
1 comorbidity	-0.176 (-0.449, 0.097)	0.139	0.147 (-0.127, 0.422)	0.140
>1 comorbidities	-0.256 (-0.491, -0.021)	0.120	0.626 (0.401, 0.851)	0.115

Table 5. Average annual costs per patient hospitalised with AF by sex

Sex	Cost estimates	
	Mean total cost (%)	95% CI
Male		
Inpatient	2935 (79.99)	(2915, 2955)
Outpatient	31 (8.46)	(308, 313)
Care home	165 (4.50)	(154, 177)
PIS	242 (6.60)	(240, 245)
Total	3669	(3872, 3927)
Female		
Inpatient	3022 (77.49)	(3001, 3042)
Outpatient	310 (7.96)	(308, 313)
Care home	268 (6.88)	(255, 281)
PIS	259 (6.64)	(256, 262)
Total	3968	(3872, 3927)

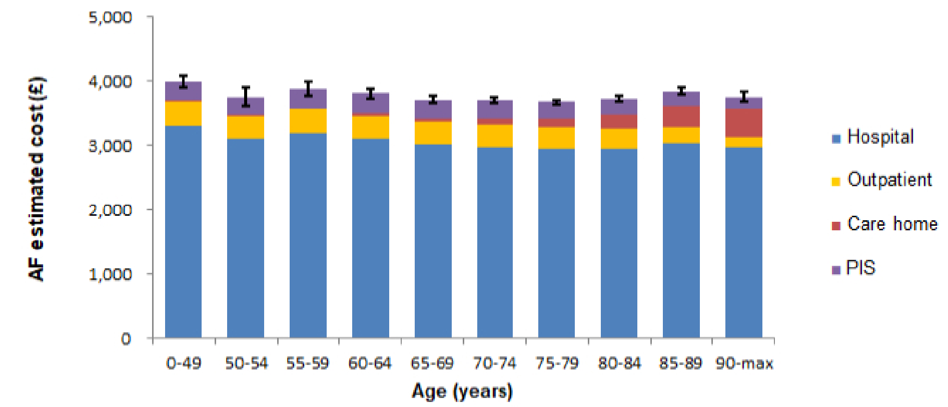

Age groups	Inpatient total cost (£) Mean (95%)	Outpatient total cost (£) Mean (95%)	Care home total cost (£) Mean (95%)	PIS total cost (£) Mean (95%)
(0-49)	3306 (3146, 3466)	382 (366, 399)	19 (12, 51)	285 (259, 311)
(50-54)	3107 (2979, 3236)	367 (354, 379)	19 (9, 47)	270 (253, 287)
(55-59)	3193 (3100, 3287)	370 (361, 379)	22 (2, 43)	290 (278, 302)
(60-64)	3107 (3044, 3170)	364 (357, 371)	47 (28, 67)	293 (285, 302)
(65-69)	3009 (2960, 3058)	363 (358, 368)	53 (37, 69)	286 (280, 291)
(70-74)	2969 (2931, 3007)	355 (351, 359)	95 (78, 111)	282 (278, 287)
(75-79)	2954 (2923, 2985)	340 (336, 343)	118 (104, 132)	267 (264, 271)
(80-84)	2962 (2930, 2994)	299 (296, 303)	225 (207, 242)	247 (244, 250)
(85-89)	3038 (3002, 3074)	249 (245, 253)	340 (318, 363)	224 (221, 228)
(90-max)	2971 (2926, 3017)	161 (157, 165)	435 (404, 466)	191 (186, 195)
Total	2978 (2964, 2993)	310 (309, 312)	226 (217, 235)	250 (248, 252)

Average annual costs per patient hospitalised with AF by sector. Cost components with confidence interval are presented for each age group.

59x47mm (300 x 300 DPI)

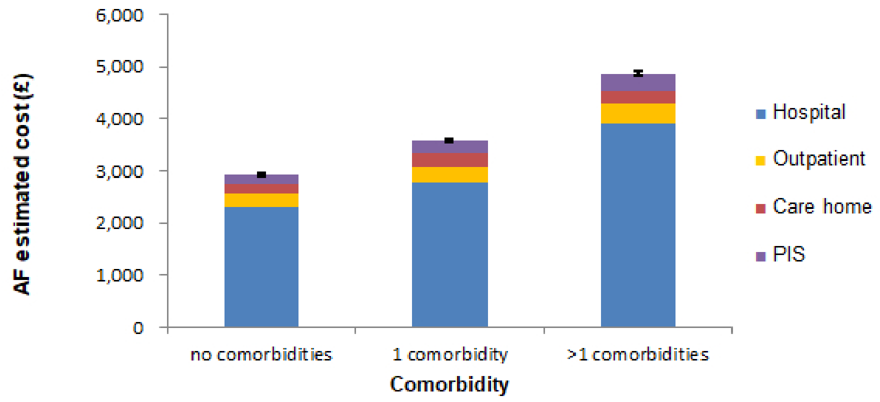

Component	No comorbidity cost (£) Mean (95%)	1 comorbidity cost (£) Mean (95%)	>1 comorbidity cost (£) Mean (95%)
Inpatient	2295 (2274, 2316)	2787 (2761, 2812)	3890 (3857, 3922)
Outpatient	276 (274, 279)	294 (291, 297)	388 (385, 392)
Care home	183 (167, 199)	249 (231, 266)	235 (221, 248)
PIS	175 (173, 177)	247 (244, 250)	348 (343, 353)
Total	2927 (2899, 2955)	3590 (3556, 3624)	4909 (4868, 4949)

Average cost per patient hospitalised with AF by Charlson Comorbidity Index. Cost components with confidence interval are presented for each Comorbidity category.

54x37mm (300 x 300 DPI)

ONLINE SUPPLEMENT

Equation I. Probability of healthcare utilisation

$$\Pr(HCE_{it} > 0) = \alpha + \beta_1 \sum_{s=2}^{10} A_{it} + \beta_2 G_i + \beta_3 Y_i + \beta_4 \sum_{s=2}^{5} S_i + \beta_5 \sum_{u=2}^{8} U_i + \beta_6 \sum_{h=2}^{14} H_i$$

$$+ \beta_7 \sum_{c=2}^{3} C_{it} + \beta_8 D_i + \left(\beta_9 \sum_{s=2}^{5} S_i * D_i \right) + \left(\beta_{10} \sum_{c=2}^{3} C_{it} * \sum_{s=2}^{10} A_{it} \right) + u_i$$

Where: A is age at the time of admission (reference category: 0-49 age group); G is sex (reference category: male); Y year of admission; S is SIMD quintile (reference category: most deprived quintile (1)); U is the urban/rural classification (reference category: large urban area);H is health board of inpatient admission (reference category: Greater Glasgow & Clyde); C is the Charlson comorbidity index (reference category: no comorbidities); D is mortality during five year follow-up; u_i is the error term for patient *i* at time *t*.

Equation II. Cost estimation

$$E[HCE] = g(x\beta)$$

Where xβ is the linear predictor for HCE

Equation III. Multiplying first and second part

$$E[HCE|X] = \Pr(HCE > 0|X) * E[HCE|HCE > 0, X]$$

For peer review only

For peer review only - http://bmjopen.bmj.com/site/about/guidelines.xhtml

Table I. Regression interactions: probability of healthcare resources utilisation and cost estimation

Covariates	Probability (1st modelling part) Coefficient (95%CI)	Std. Err	Probability (2nd modelling part) Coefficient (95%CI)	Std. Err
**Interaction: SIMD - Mortality within 5 years**				
1	Reference			
2	-0.015 (-0.069, 0.038)	0.027	0.056 (0.027, 0.086)	0.015
3	0.046 (-0.007, 0.100)	0.027	0.067 (0.036, 0.097)	0.016
4	0.017 (-0.037, 0.071)	0.028	0.089 (0.059, 0.120)	0.016
5	0.069 (0.013, 0.125)	0.029	0.100 (0.069, 0.132)	0.016
**Interaction: age (year) - Charlson score (1 comorbidity)**				
0-49	Reference			
50-54	-0.195 (-0.342, -0.049)	0.075	-0.016 (-0.124, 0.092)	0.055
55-59	-0.384 (-0.514, -0.255)	0.066	-0.081 (-0.173, 0.012)	0.047
60-64	-0.459 (-0.579, -0.340)	0.061	-0.116 (-0.202, -0.031)	0.044
65-69	-0.500 (-0.613, -0.386)	0.058	-0.161 (-0.244, -0.078)	0.042
70-74	-0.510 (-0.621, -0.399)	0.057	-0.202 (-0.283, -0.121)	0.041
75-79	-0.570 (-0.680, -0.461)	0.056	-0.197 (-0.276, -0.117)	0.041
80-84	-0.594 (-0.704, -0.484)	0.056	-0.209 (-0.290, -0.128)	0.041
85-89	-0.643 (-0.756, -0.531)	0.058	-0.215 (-0.298, -0.132)	0.042
90-max	-0.709 (-0.828, -0.590)	0.061	-0.267 (-0.357, -0.178)	0.045
**Interaction: age (year) - Charlson score (>1 comorbidities)**				
0-49	Reference			
50-54	-0.449 (-0.685, -0.213)	0.121	-0.214 (-0.316, -0.111)	0.052
55-59	-0.539 (-0.751, -0.327)	0.108	-0.209 (-0.305, -0.112)	0.049
60-64	-0.534 (-0.734, -0.334)	0.102	-0.323 (-0.412, -0.234)	0.045
65-69	-0.573 (-0.767, -0.378)	0.099	-0.436 (-0.523, -0.350)	0.044
70-74	-0.650 (-0.842, -0.459)	0.098	-0.520 (-0.604, -0.436)	0.043
75-79	-0.767 (-0.957, -0.577)	0.097	-0.556 (-0.639, -0.473)	0.042
80-84	-0.857 (-1.047, -0.667)	0.097	-0.625 (-0.709, -0.541)	0.043
85-89	-0.967 (-1.159, -0.775)	0.098	-0.661 (-0.747, -0.576)	0.044
90-max	-1.074 (-1.270, -0.878)	0.100	-0.805 (-0.896, -0.714)	0.046

y only

For peer review only - http://bmjopen.bmj.com/site/about/guidelines.xhtml

Equation IV. Probability of healthcare utilisation (alive at the end of the five-year follow-up period)

$$\Pr(HCE_{it} > 0) = \alpha + \beta_1 \sum_{s=2}^{10} A_{it} + \beta_2 G_i + \beta_3 Y_i + \beta_4 \sum_{s=2}^{5} S_i + \beta_5 \sum_{u=2}^{8} U_i + \beta_6 \sum_{h=2}^{14} H_i$$

$$+ \beta_7 \sum_{c=2}^{3} C_{it} + \left(\beta_8 \sum_{c=2}^{3} C_{it} * \sum_{s=2}^{10} A_{it} \right) + u_i$$

Where: A is age at the time of admission (reference category: 0 -49 age group); G is sex (reference category: male); Y year of admission; S is SIMD quintile (reference category: most deprived quintile (1)); U is the urban/rural classification (reference category: large urban area);H is health board of inpatient admission (reference category: Greater Glasgow & Clyde); C is the Charlson comorbidity index (reference category: no comorbidities); u_i is the error term for patient *i* at time *t*.

For peer review only

For peer review only - http://bmjopen.bmj.com/site/about/guidelines.xhtml

Table II. Regression interactions: probability of healthcare resources utilisation and cost estimation (alive at the end of the five-year follow-up period)

Covariates	Probability (1st modelling part) Coefficient (95%CI)	Std. Err	Probability (2nd modelling part) Coefficient (95%CI)	Std. Err
**Interaction: age (year) - Charlson score (1 comorbidity)**				
0-49	Reference			
50-54	-0.190 (-0.341, -0.039)	0.077	-0.015 (-0.126, 0.097)	0.057
55-59	-0.400 (-0.534, -0.266)	0.069	-0.087 (-0.187, 0.014)	0.051
60-64	-0.484 (-0.608, -0.360)	0.063	-0.116 (-0.208, -0.023)	0.047
65-69	-0.531 (-0.649, -0.412)	0.060	-0.143 (-0.233, -0.053)	0.046
70-74	-0.559 (-0.674, -0.443)	0.059	-0.195 (-0.283, -0.107)	0.045
75-79	-0.635 (-0.750, -0.521)	0.058	-0.207 (-0.293, -0.121)	0.044
80-84	-0.680 (-0.796, -0.565)	0.059	-0.215 (-0.304, -0.125)	0.046
85-89	-0.730 (-0.850, -0.610)	0.061	-0.254 (-0.349, -0.159)	0.048
90-max	-0.827 (-0.959, -0.695)	0.067	-0.281 (-0.393, -0.169)	0.057
**Interaction: age (year) - Charlson score (>1 comorbidities)**				
0-49	Reference			
50-54	-0.408 (-0.658, -0.158)	0.127	-0.226 (-0.342, -0.109)	0.059
55-59	-0.504 (-0.726, -0.281)	0.114	-0.235 (-0.348, -0.122)	0.058
60-64	-0.525 (-0.735, -0.316)	0.107	-0.339 (-0.443, -0.236)	0.053
65-69	-0.580 (-0.784, -0.377)	0.104	-0.448 (-0.549, -0.347)	0.051
70-74	-0.713 (-0.913, -0.513)	0.102	-0.565 (-0.663, -0.467)	0.050
75-79	-0.820 (-1.019, -0.621)	0.101	-0.648 (-0.745, -0.552)	0.049
80-84	-0.938 (-1.137, -0.739)	0.102	-0.702 (-0.801, -0.603)	0.051
85-89	-1.074 (-1.276, -0.872)	0.103	-0.769 (-0.873, -0.665)	0.053
90-max	-1.196 (-1.406, -0.986)	0.107	-0.932 (-1.051, -0.814)	0.060

For peer review only - http://bmjopen.bmj.com/site/about/guidelines.xhtml

Figure I. Average annual costs per patient hospitalised with AF by sector. Cost components with confidence interval are presented for each age group (alive at the end of the five-year follow-up period)

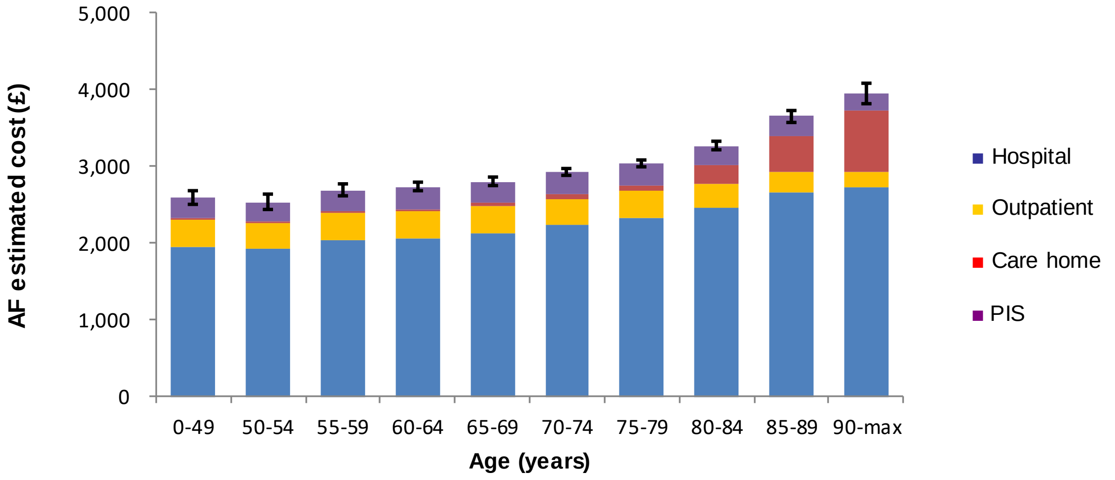

Age groups	Inpatient total cost (£) Mean (95%)	Outpatient total cost (£) Mean (95%)	Care home total cost (£) Mean (95%)	PIS total cost (£) Mean (95%)
(0-49)	1956 (1846, 2066)	360 (344, 375)	17 (16, 49)	267 (243, 291)
(50-54)	1916 (1826, 2007)	344 (332, 356)	14 (8, 37)	259 (242, 275)
(55-59)	2041 (1973, 2110)	351 (342, 360)	17 (1,37)	280 (269, 292)
(60-64)	2071 (2022, 2119)	351 (344, 358)	26 (13, 38)	290 (281, 299)
(65-69)	2135 (2093, 2178)	352 (347, 357)	32 (21, 44)	285 (279, 291)
(70-74)	2230 (2195, 2266)	352 (347, 356)	63 (49, 76)	286 (281, 291)
(75-79)	2329 (2297, 2361)	346 (342, 350)	84 (71, 97)	277 (273, 281)
(80-84)	2457 (2420, 2494)	314 (310, 318)	236 (212, 260)	266 (262, 269)
(85-89)	2651 (2603, 2698)	272 (267, 278)	480 (439, 522)	252 (248, 257)
(90-max)	2734 (2663, 2806)	188 (181, 194)	811 (735, 887)	225 (219, 231)
Total	2253 (2238, 2267)	321 (319, 323)	199 (188, 209)	264 (262, 266)

For peer review only - http://bmjopen.bmj.com/site/about/guidelines.xhtml

Equation V. Probability of healthcare utilisation (dead at the end of the five-year follow-up period)

$$\Pr(HCE_{it} > 0) = \alpha + \beta_1 \sum_{s=2}^{10} A_{it} + \beta_2 G_i + \beta_3 Y_i + \beta_4 \sum_{s=2}^{5} S_i + \beta_5 \sum_{u=2}^{8} U_i + \beta_6 \sum_{h=2}^{14} H_i$$

$$+ \beta_7 \sum_{c=2}^{3} C_{it} + \left(\beta_8 \sum_{c=2}^{3} C_{it} * \sum_{s=2}^{10} A_{it} \right)$$

Where: A is age at the time of admission (reference category: 0 -49 age group); G is sex (reference category: male); Y year of admission; S is SIMD quintile (reference category: most deprived quintile (1)); U is the urban/rural classification (reference category: large urban area);H is health board of inpatient admission (reference category: Greater Glasgow & Clyde); C is the Charlson comorbidity index (reference category: no comorbidities); u_i is the error term for patient *i* at time *t*.

Note: the model for care home does not include the 0 -49 and 50-54 age groups, as none of those patients incurred any cost related to care home.

For peer review only - http://bmjopen.bmj.com/site/about/guidelines.xhtml

Table III. Regression interactions: probability of healthcare resources utilisation and cost estimation (dead at the end of the five-year follow-up period)

Covariates	Probability (1st modelling part)		Probability (2nd modelling part)	
	Coefficient (95%CI)	Std. Err	Coefficient (95%CI)	Std. Err
**Interaction: age (year) - Charlson score (1 comorbidity)**				
0-49	Reference			
50-54	-0.057 (-0.456, 0.343)	0.204	0.124 (-0.268, 0.515)	0.200
55-59	0.130 (-0.207, 0.468)	0.172	0.054 (-0.282, 0.389)	0.171
60-64	0.083 (-0.223, 0.389)	0.156	-0.009 (-0.309, 0.291)	0.153
65-69	0.028 (-0.263, 0.320)	0.149	-0.054 (-0.342, 0.234)	0.147
70-74	-0.006 (-0.290, 0.278)	0.145	-0.051 (-0.334, 0.232)	0.144
75-79	-0.078 (-0.358, 0.201)	0.143	0.002 (-0.278, 0.282)	0.143
80-84	-0.200 (-0.478, 0.078)	0.142	-0.048 (-0.326, 0.230)	0.142
85-89	-0.160 (-0.437, 0.117)	0.141	-0.039 (-0.316, 0.239)	0.142
90-max	-0.154 (-0.431, 0.123)	0.141	-0.079 (-0.358, 0.200)	0.142
**Interaction: age (year) - Charlson score (>1 comorbidities)**				
0-49	Reference			
50-54	0.000 (-0.340, 0.339)	0.173	0.066 (-0.268, 0.400)	0.170
55-59	-0.147 (-0.436, 0.142)	0.147	0.041 (-0.240, 0.322)	0.143
60-64	-0.135 (-0.398, 0.128)	0.134	-0.114 (-0.360, 0.132)	0.126
65-69	-0.215 (-0.466, 0.036)	0.128	-0.164 (-0.401, 0.074)	0.121
70-74	-0.192 (-0.436, 0.053)	0.125	-0.237 (-0.470, -0.005)	0.119
75-79	-0.311 (-0.553, -0.070)	0.123	-0.248 (-0.478, -0.018)	0.117
80-84	-0.443 (-0.683, -0.204)	0.122	-0.367 (-0.595, -0.139)	0.116
85-89	-0.394 (-0.633, -0.154)	0.122	-0.394 (-0.623, -0.166)	0.116
90-max	-0.312 (-0.551, -0.073)	0.122	-0.483 (-0.713, -0.254)	0.117

For peer review only - http://bmjopen.bmj.com/site/about/guidelines.xhtml

Figure II. Average annual costs per patient hospitalised with AF by sector. Cost components with confidence interval are presented for each age group (dead at the end of the five-year follow-up period)

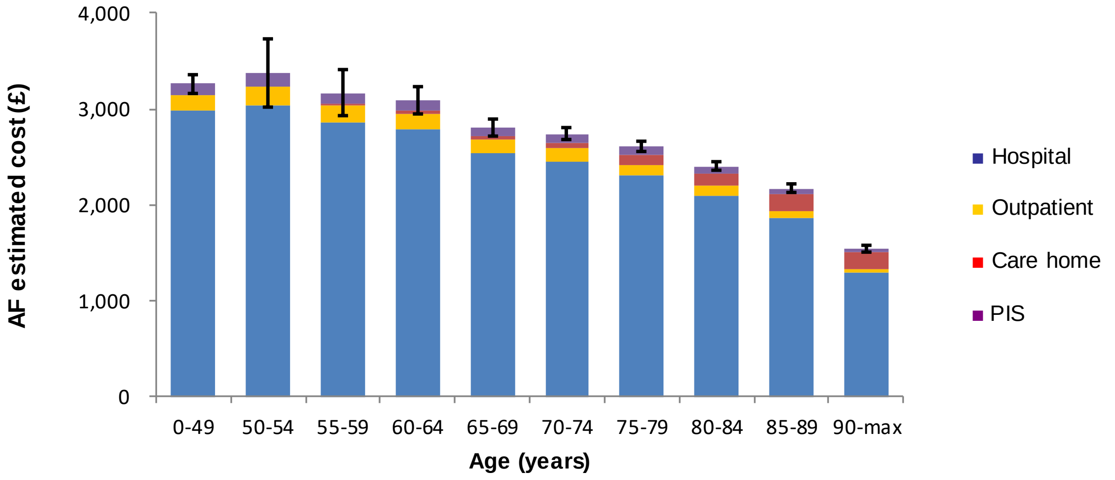

Age groups	Inpatient total cost (£) Mean (95%)	Outpatient total cost (£) Mean (95%)	Care home total cost (£) Mean (95%)	PIS total cost (£) Mean (95%)
(0-49)	2976 (2603, 3349)	161 (136, 186)	n/a	125 (65, 185)
(50-54)	3037 (2712, 3362)	203 (177, 228)	n/a	130 (97, 164)
(55-59)	2865 (2649, 3080)	181 (165, 197)	9 (5, 23)	115 (95, 135)
(60-64)	2795 (2661, 2929)	162 (153, 170)	28 (3, 53)	111 (99, 122)
(65-69)	2536 (2450, 2621)	154 (147, 160)	26 (12, 39)	95 (88, 102)
(70-74)	2448 (2386, 2510)	138 (134, 142)	60 (42, 78)	97 (91, 102)
(75-79)	2302 (2257, 2347)	118 (115, 121)	100 (80, 120)	83 (80, 86)
(80-84)	2099 (2062, 2136)	93 (91, 95)	139 (121, 157)	70 (67, 72)
(85-89)	1859 (1826, 1892)	69 (67, 71)	187 (167, 206)	58 (56, 60)
(90-max)	1289 (1263, 1316)	33 (31, 34)	180 (162, 199)	35 (34, 36)
Total	2018 (2001, 2034)	91 (90, 93)	142 (132, 152)	66 (65, 67)

Note: the care home total cost estimation does not include the 0 -49 and 50-54 age groups, as none of those patients incurred any cost related to care home.

For peer review only - http://bmjopen.bmj.com/site/about/guidelines.xhtml

STROBE 2007 (v4) Statement—Checklist of items that should be included in reports of *cohort studies*

Section/Topic	Item #	Recommendation	Reported on page #
**Title and abstract**	1	(*a*) Indicate the study's design with a commonly used term in the title or the abstract	1
		(*b*) Provide in the abstract an informative and balanced summary of what was done and what was found	1
**Introduction**			
Background/rationale	2	Explain the scientific background and rationale for the investigation being reported	3
Objectives	3	State specific objectives, including any prespecified hypotheses	3
**Methods**			
Study design	4	Present key elements of study design early in the paper	4
Setting	5	Describe the setting, locations, and relevant dates, including periods of recruitment, exposure, follow-up, and data collection	4
Participants	6	(*a*) Give the eligibility criteria, and the sources and methods of selection of participants. Describe methods of follow-up	4
		(*b*) For matched studies, give matching criteria and number of exposed and unexposed	n/a
Variables	7	Clearly define all outcomes, exposures, predictors, potential confounders, and effect modifiers. Give diagnostic criteria, if applicable	7-8
Data sources/ measurement	8*	For each variable of interest, give sources of data and details of methods of assessment (measurement). Describe comparability of assessment methods if there is more than one group	n/a
Bias	9	Describe any efforts to address potential sources of bias	7
Study size	10	Explain how the study size was arrived at	4
Quantitative variables	11	Explain how quantitative variables were handled in the analyses. If applicable, describe which groupings were chosen and why	7-8
Statistical methods	12	(*a*) Describe all statistical methods, including those used to control for confounding	6-8
		(*b*) Describe any methods used to examine subgroups and interactions	6-7
		(*c*) Explain how missing data were addressed	n/a
		(*d*) If applicable, explain how loss to follow-up was addressed	n/a
		(*e*) Describe any sensitivity analyses	n/a
**Results**			

For peer review only

Participants	13*	(a) Report numbers of individuals at each stage of study—eg numbers potentially eligible, examined for eligibility, confirmed eligible, included in the study, completing follow-up, and analysed	n/a
		(b) Give reasons for non-participation at each stage	n/a
		(c) Consider use of a flow diagram	n/a
Descriptive data	14*	(a) Give characteristics of study participants (eg demographic, clinical, social) and information on exposures and potential confounders	n/a
		(b) Indicate number of participants with missing data for each variable of interest	n/a
		(c) Summarise follow-up time (eg, average and total amount)	n/a
Outcome data	15*	Report numbers of outcome events or summary measures over time	n/a
Main results	16	(*a*) Give unadjusted estimates and, if applicable, confounder-adjusted estimates and their precision (eg, 95% confidence interval). Make clear which confounders were adjusted for and why they were included	9-11
		(*b*) Report category boundaries when continuous variables were categorized	n/a
		(*c*) If relevant, consider translating estimates of relative risk into absolute risk for a meaningful time period	n/a
Other analyses	17	Report other analyses done—eg analyses of subgroups and interactions, and sensitivity analyses	9
**Discussion**			
Key results	18	Summarise key results with reference to study objectives	11
**Limitations**			
Interpretation	20	Give a cautious overall interpretation of results considering objectives, limitations, multiplicity of analyses, results from similar studies, and other relevant evidence	12-14
Generalisability	21	Discuss the generalisability (external validity) of the study results	14
**Other information**			
Funding	22	Give the source of funding and the role of the funders for the present study and, if applicable, for the original study on which the present article is based	15

*Give information separately for cases and controls in case-control studies and, if applicable, for exposed and unexposed groups in cohort and cross-sectional studies.

Note: An Explanation and Elaboration article discusses each checklist item and gives methodological background and published examples of transparent reporting. The STROBE checklist is best used in conjunction with this article (freely available on the Web sites of PLoS Medicine at http://www.plosmedicine.org/, Annals of Internal Medicine at http://www.annals.org/, and Epidemiology at http://www.epidem.com/). Information on the STROBE Initiative is available at www.strobe-statement.org.

For peer review only

BMJ Open

A Two-Part Model to estimate Inpatient, Outpatient, Prescribing and Care Home Costs associated with Atrial Fibrillation in Scotland

Journal:	*BMJ Open*
Manuscript ID	bmjopen-2018-028575.R3
Article Type:	Original research
Date Submitted by the Author:	16-Dec-2019

Complete List of Authors:	Ciminata, Giorgio; University of Glasgow Institute of Health and Wellbeing, Health Economics and Health Technology Assessment (HEHTA)<br>Geue, Claudia; University of Glasgow, Health Economics and Health Technology Assessment (HEHTA)<br>Langhorne, Peter; University of Glasgow, Institute of Cardiovascular and Medical Sciences<br>Wu, O; University of Glasgow, Health Economics and Health Technology Assessment (HEHTA)

**Primary Subject Heading**:	Health economics
Secondary Subject Heading:	Cardiovascular medicine
Keywords:	HEALTH ECONOMICS, Atrial Fibrillation, Stroke < NEUROLOGY, Cost Analysis

SCHOLARONE™
Manuscripts

I, the Submitting Author has the right to grant and does grant on behalf of all authors of the Work (as defined in the below author licence), an exclusive licence and/or a non-exclusive licence for contributions from authors who are: i) UK Crown employees; ii) where BMJ has agreed a CC-BY licence shall apply, and/or iii) in accordance with the terms applicable for US Federal Government officers or employees acting as part of their official duties; on a worldwide, perpetual, irrevocable, royalty-free basis to BMJ Publishing Group Ltd ("BMJ") its licensees and where the relevant Journal is co-owned by BMJ to the co-owners of the Journal, to publish the Work in this journal and any other BMJ products and to exploit all rights, as set out in our licence.

The Submitting Author accepts and understands that any supply made under these terms is made by BMJ to the Submitting Author unless you are acting as an employee on behalf of your employer or a postgraduate student of an affiliated institution which is paying any applicable article publishing charge ("APC") for Open Access articles. Where the Submitting Author wishes to make the Work available on an Open Access basis (and intends to pay the relevant APC), the terms of reuse of such Open Access shall be governed by a Creative Commons licence – details of these licences and which Creative Commons licence will apply to this Work are set out in our licence referred to above.

Other than as permitted in any relevant BMJ Author's Self Archiving Policies, I confirm this Work has not been accepted for publication elsewhere, is not being considered for publication elsewhere and does not duplicate material already published. I confirm all authors consent to publication of this Work and authorise the granting of this licence.

For peer review only

A Two-Part Model to estimate Inpatient, Outpatient, Prescribing and Care Home Costs associated with Atrial Fibrillation in Scotland.

Authors: Ciminata G (Research Assistant)[1], Geue C (Lecturer in Health Economics)[1], Langhorne P (Professor of Stroke Care)[2], Wu O (Professor of Health Technology Assessment)[1].

1. Health Economics and Health Technology Assessment (HEHTA), Institute of Health & Wellbeing, University of Glasgow, Glasgow G12 8RZ, UK
2. Institute of Cardiovascular & Medical Sciences, University of Glasgow, Glasgow G31 2ER, UK

Corresponding author:
Mr Giorgio Ciminata (MSc), g.ciminata.1@research.gla.ac.uk

Cover title: Inpatient, Outpatient, Prescribing and Care Home Costs associated with Atrial Fibrillation

Total number of tables and figures: Figures: 2, Tables: 5

Subject terms: Atrial Fibrillation

Key words: Atrial Fibrillation, Stroke, cost analysis

Word count: 3753

For peer review only

Abstract

Objective: To estimate global inpatient, outpatient, prescribing and care-home costs for atrial fibrillation (AF) patients, using population-based, individual-level linked data.

Design: A two-part model was employed to estimate the probability of resource utilisation and costs conditional on positive utilisation using individual-level linked data.

Settings: Scotland, five years following first hospitalisation for AF between 1997 and 2015.

Participants: Patients hospitalised with a known diagnosis of AF or atrial flutter.

Primary and secondary outcome measures: Inpatient, outpatient, prescribing and care home costs.

Results: The mean annual cost for an AF patient was estimated at £3785 (95% CI £3767-£3804). Inpatient admissions and outpatient visits accounted for 79% and 8% of total costs, respectively; prescriptions and care home stay accounted for 7% and 6% of total costs. Inpatient cost was the main driver across all age groups. While inpatient cost contributions (~80%) were constant between 0 and 84 years, they decreased for patients over 85 years. This is offset by increasing care-home cost contributions. Mean annual costs associated with AF increased significantly with increasing number of comorbidities.

Conclusion: This study used a contemporary and representative cohort, and a comprehensive approach to estimate global costs associated with AF, taking into account resource utilisation beyond hospital care. While overall costs, considerably affected by comorbidity, did not increase with increasing age, care-home costs increased proportionally with age. Inpatient admission was the main contributor to the overall financial burden of AF, highlighting the need for improved mechanisms of early diagnosis to prevent hospitalisations.

For peer review only

Article summary

Strengths and limitations of this study

- Costs are estimated through an incidence-based approach using patient-level morbidity records.
- Sufficient follow-up time is used to capture all relevant global costs to generate a contemporary estimate of health and care home costs related to AF.
- Scotland offers a robust record linkage system, where administrative patient-level health data are routinely collected.
- Data on primary care consultations were not available for linkage at a national level, however the impact this might have on overall costs is expected to be small.
- The potential risk of AF going undiagnosed and clinical miscoding of morbidity records may lead to an underestimation of the AF cohort and associated costs.

For peer review only

Introduction

Atrial Fibrillation (AF) is the most common form of arrhythmia. In Scotland AF affects 1.8% of the adult population, and rises to 6% among those aged 65 years or over [1]. In an ageing population, AF has a substantial impact on the economic burden of the healthcare system.

A number of cost analyses on estimating the economic burden of AF exist. The majority of these studies used various definition of the AF study population , based on data sourced from administrative database [2-4], health insurance databases [2, 5-7], hospital records [8, 9] and surveys [10]. Direct medical costs related to inpatient admissions, outpatient visits, as well as prescriptions have been included in these estimates; [2-10] indirect costs related to loss of productivity have been estimated among patients who were at working ages [6, 7].

There is a lack of generalisable studies based on large national population datasets that examine the total and the distribution of costs associated with AF [11]. The aim of this study was to quantify the inpatient, outpatient, prescribing and care home costs associated with AF over a five-year period. Using record-linkage of national datasets from Scotland, we also examined the distribution of costs that are attributable to AF.

Methods

Cost analyses or cost of illness studies typically adopt either the prevalence or incidence based approaches [12]. In the context of AF, the prevalence-based approach determines costs attributable to all cases of AF in a given year, while the incidence-based approach determines costs of new cases of AF in a given time period. In the present study, costs were estimated with an incidence-based approach. A further distinction between costing analyses is between the medicalized and the global comprehensive approaches.

For peer review only

In the first case, only expenditures directly attributable to a particular disease are used for estimating the overall costs. While the medicalized approach can be used to identify highly specific expenditures, it may also lead to underestimation or overestimation of the economic burden of a given disease; this may happen when cost estimation is not adequately adjusted for confounders highly correlated with the disease of interest. Conversely, the global comprehensive approach, used in this analysis, includes all the expenditures incurred by a population with a particular disease [13]. These expenditures are not necessarily related to the disease of interest; for instance, expenditures related to orthopaedics surgery or cancer treatment incurred by a patient with AF, will count towards the global comprehensive cost of AF.

Data

Data were obtained from the Information Services Division (ISD) of NHS Scotland as part of a wider project that used routinely collected data to evaluate clinical effectiveness and cost-effectiveness of Direct Oral Anticoagulants (DOACs) in the prevention of stroke in the AF population. Inpatient records for patients with a diagnosis of AF or atrial flutter between 1997 and 2015 were extracted from the General Acute Inpatient and Day Case Scottish Morbidity Records 01 (SMR01). These records contain all general acute admissions, categorized as inpatients or day cases, discharged from non-obstetric and non-psychiatric specialties [14]. Incident AF events (ICD10 code I48) were identified using all six diagnostic positions in SMR01, with a look back period of five years to minimise double counting. After checking for data entry errors and removal of duplicate records, the final AF cohort consisting of 278,286 individuals hospitalised with a diagnosis of AF or atrial flutter was identified.

For peer review only

Individual-level data linkage was then carried out with outpatient clinic attendance (Outpatient Attendance Scottish Morbidity Records 00; SMR00), the prescribing information system (PIS), care home census and mortality records (National Records for Scotland, NRS). Records from SMR00 include information on new and follow up outpatient appointments for any clinical specialty [15]. The PIS database includes prescribing records for all medicines and their associated costs, which are prescribed and dispensed by community pharmacies, dispensing doctors and a small number of specialist appliance suppliers [16]. The quality of PIS data is guaranteed by an electronic data capture, and it passes several stages of quality control before and after data are submitted [17]. The care home census combines the former Residential Care Home Census (run by the Scottish Government) and the Private Nursing Homes Census (run by ISD Scotland). Items reported in the care home census include discharge dates to care home residency such as NHS and private nursing homes, as well as an indication on whether nursing care is required [16].

Patients were followed up for five years following incident AF event in terms of their healthcare resource use, care home admissions and mortality. Since AF is often a precursor of stroke and cardiovascular conditions, an estimation of costs for a period of five years post AF event would allow us to fully capture costs associated with an AF patient.

For peer review only

Costing

Inpatient care costs were obtained from the latest (2013/2014) Scottish National Tariff (SNT), a list of standard average prices based on Healthcare Resource Groups (HRGs) [17, 18]. The SNT uses HRG4 for grouping clinically similar treatments that use similar levels of healthcare resources. After defining a total cost per episode, the total cost for a continuous inpatient stay (CIS) was calculated.

A CIS describes the entire duration of an inpatient stay from the date of admission to the date of discharge and can consist of several episodes in different specialties. Since the SNT is based on spells of care (inpatient stay within the same specialty) rather than individual inpatient episodes or a CIS, a CIS was partitioned into spells when a change in specialty occurred [17]. If within a CIS, two or more episodes were in the same specialty, only the highest incurred cost was taken into account, and the remaining episodes were replaced with a zero cost. Outpatient costs were obtained by assigning outpatient specialty costs, to outpatient attendances [17]. Unit costs were specific to whether the outpatient attendance took place at a consultant or nurse led clinic [15].

The cost of each prescription dispensed per patient was obtained from PIS [19]. Firstly, the price per unit was obtained by dividing the item price by the pack size. Secondly, the total number of items dispensed was obtained by multiplying the number of items dispensed by the number of instalments. Care home costs, obtained from the care home census, were based on length of stay or residency. Care home residency was established from care home census records, reporting admission to a care home like structure [16]. An average of care home charges for long stay residents was calculated using information on whether nursing care was provided or not. The average weekly care home charge was expressed per day, so that only the effective days spent in a care home were costed. The tariffs used for costing account for inflation, therefore further cost adjustment was not needed.

Econometric model

Healthcare expenditure data are typically characterised by: i) a significant proportion of zero-cost observations for individuals who have not utilised any healthcare resources in a given time period, and ii) a skewed distribution for positive costs. A two-part model was used [20, 21].

For peer review only

In the first part of the model, the probability of using a healthcare service in a given time period was estimated using a probit model (Equation I, please see online supplement). The same explanatory variables were used in the second part of the model, with a gamma distribution and log link, estimating costs conditional on having incurred positive costs (Equation II, please see online supplement). Mean costs per patient per year following their incident AF event were calculated by multiplying first and second modelling part (Equation III, please see online supplement).

In order to account for the skewed nature of cost data, generalised linear models (GLMs) were used. These were compared against ordinary least squares regression (OLS) and log transformed OLS by means of the Akaike Information Criterion (AIC), which measures goodness of fit. When comparing the different models, GLM reported the lowest AIC indicating the best fit for the given set of data. A user-written STATA programme "glmdiagnostic.do" [20], performing four different tests simultaneously, was used to identify the most appropriate distributional family and link function.

Econometric model covariates

The two-part model adjusted for age, sex, year of inpatient admission, socio-economic status, urban-rural classification, health board, comorbidities and mortality. These covariates are considered to be the main confounders that have an effect on costs incurred by an AF population. We controlled for age because AF and associated comorbidities are age-related conditions, and may have an impact on the overall costs. We also assumed costs to vary between males and females, in particular those for care home residency. Variation in healthcare utilisation and associated costs and care home residency by socio-economic status is controlled for using the Scottish Index of Multiple Deprivation (SIMD).

For peer review only

The SIMD reflects areas of multiple deprivation ranked from the most to the least deprived and expressed as quintiles where the most and the least deprived areas are represented by 1 and 5 respectively [22]. In Scotland, there are 14 regional health boards responsible for the provision of healthcare [23]. Hence, potential differences in healthcare utilisation and prescribing costs, may reflect variation in clinical practice and prescribing behaviour rather than the ability of patients to access care. Patients living in urban areas may have easier access to care compared to patients living in more remote areas, which is controlled for including the 8-fold classification measuring rurality [24].

Patients with one or more comorbidities are expected to incur significantly higher costs than those with none. We accounted for this by including the Charlson comorbidity index, where 1 indicates the absence of comorbidities, 2 the presence of only a single comorbidity and 3 the presence of more than one comorbidity [25]. Two interaction terms between age and comorbidities, and mortality and SIMD were included in the econometric model. Intuitively, a relationship of direct proportionality between age and comorbidities suggests that the level of comorbidities increases, as patients get older. Similarly, the socio-economic status may significantly influence the rate of socio-economic inequalities in mortality [26].

Sensitivity analyses

In order to ascertain whether mortality had an impact on overall AF related healthcare costs, average annual cost per patient by age and for each health or care home sector, was estimated for patients who were alive and those who were dead at the end of the five-year follow-up period. The two econometric models (Equation IV and V, please see online TABLEent) followed the same structure of the model described in the previous section and used for the main analysis; however, those models were not adjusted for mortality.

For peer review only

Ethics statement

No ethics approval was sought as this study does not require consenting/contacting patients directly. All data used by researchers is pseudonymised, data reported is aggregated to minimise risk of identification and output clearance is required.

Patients and public involvement

There was no patients or public involvement

Results

Cohort characteristics

Of the 278,286 AF patients with a mean age of 74 years (SD 12.5), the majority were identified in the two largest urban health board areas (Greater Glasgow & Clyde and Lothian), accounting for 22.2% and 14.8% respectively. This is also reflected in our categorisation of geographical areas, where large urban represented 38.4% and other urban areas represented 29.7% of the total AF cohort. Greater proportion of patients live in areas belonging to the most deprived quintile compared with those living in the least deprived areas – SIMD quintile 1 and quintile 5 representing 22.5% and 16.6% of the AF cohort respectively (Table1).

Econometric modelling results

Regression results for both modelling parts are presented in Table 2. Overall, an inversely U-shaped association between age and the likelihood of utilising any health or social care services was observed – a gradual increment in the likelihood in resource use with advancing age up to 80 years, when compared with the reference group (0-49 years), while patients 80 years or older showing a decreased probability of utilising healthcare services. However, this association was not observed in the second modelling part model, estimating costs conditional

For peer review only

on having incurred positive costs, where a statistically significant gradient between age and costs indicated increasing costs as the cohort ages.

The use of health or social care services and associated costs also increased significantly for patients living in the most deprived areas, when compared with patients living in areas with the lowest level of deprivation. The effect of socio-economic status on healthcare utilisation was also measured for those who are alive at the end of the five-year follow-up period through an interaction term between SIMD and mortality, but no statistically significant effect was found. Full details of regression results for interaction terms are presented in the supplementary online material (Table I, please see online supplement).

For patients with comorbidities, the probabilities of utilising healthcare services were greater than the probability for those with no comorbidities. Although healthcare utilisation increased with the number of comorbidities, the interaction term between age and comorbidities indicated that as patients get older the use of healthcare services on average is lower for patients with one or more comorbidities than those with none. The decrease in healthcare utilisation by age is more pronounced in patients with more comorbidities than in those with only one comorbidity. The difference in healthcare costs between comorbidity categories indicated that in the presence of one or more comorbidities, on average healthcare costs decrease as patients get older. Full details of regression results for patients who were alive and those who were dead at the end of the five-year follow-up period are presented in Table 3 and Table 4 respectively, while regression results for interaction terms are presented in the supplementary online material (Table II and Table III, please see online supplement).

Cost estimates

For peer review only

The estimated mean annual cost per AF patient was £3785 (95% CI: £3767-£3804). The estimated total costs and distribution of costs according to sex are shown in Table 5.

While there is little difference between the total costs and the distribution of costs for inpatient, outpatient and prescription costs, the difference seems more pronounced when comparing the care home component of costs (5% of total costs among males vs 7% of total costs among females).

The average annual cost per AF patient by age and for each health or care home sector is shown in Figure 1. Considering the individual contribution of each cost component to the overall costs, inpatient costs was the main driver across all age groups. While inpatient cost contribution remained constant with an average contribution of about 80% to the overall costs for patients aged between 0 and 84 years, it decreased for patients over 85 years of age. Similar patterns were observed for outpatient and prescribing costs. On the contrary, the contribution of care home costs to the overall costs increased with age (0.5% for patients aged 0-49 years and approximately 11% for patients who are 90 years or older). The contribution of each setting to the total health and care home costs by the number of existing comorbidities is illustrated in Figure 2. While inpatient and total costs vary considerably with the number of comorbidities, outpatient and care home contributions remain fairly constant.

The estimated mean annual cost per AF patient alive at the end of the five-year follow-up period was £3047 (95% CI: £3027-£3067). The average annual cost per AF patient by age and for each health or care home sector is presented in the supplementary online material (Figure I, please see online supplement). For these patients, inpatient cost was the main driver across all age groups; a gradient between age and costs indicated increasing costs as the cohort ages.

For peer review only

Similar patterns were observed for care home costs. On the contrary, outpatient and prescribing costs remained constant up to 74 years, but decreased slightly for older patients.

The estimated mean annual cost per AF patient who died during the five-year follow-up period, was £2304 (95% CI: £2284-£2324) (Figure II, please see online supplement). For these patients, inpatient cost was the main driver across all age groups; a gradient between age and costs indicated decreasing costs as the cohort ages. This was also observed for outpatient and prescribing costs; but care home costs on average increased across age groups.

Discussion

A greater proportion of AF patients were found in areas with the highest index of deprivation. This, combined with the likelihood for people living in the most deprived quintile having longer inpatient stays due to a lack of support at home, may explain the difference in inpatient care utilisation between patients from the most and the least deprived areas, with associated costs being higher for the former group. As AF is more likely to affect the elderly, so that costs were expected to increase with age. As health deteriorates with age, older age groups are assumed to make greater use of healthcare services, and therefore incur higher costs than younger age groups. However, age was found to have a modest impact on overall healthcare costs, being fairly consistent across age groups. This finding is in line with existing evidence indicating that healthcare expenditure depends not only on patients' calendar age, but is also significantly associated with remaining lifetime [27].

Any observed correlation between healthcare expenditure and age may therefore be attributable to the fact that the proportion of patients who are at the end of their lives is substantially greater in older rather than younger age groups [27]. On the other hand, comorbidity had a considerable

For peer review only

effect on the overall costs, increasing significantly in patients with more than one comorbidity. However, the decrease in healthcare utilisation by age is more pronounced in patients with more comorbidities than in those with only one comorbidity.

Decreasing inpatient and outpatient costs for the oldest patients were offset by increasing care home costs, in particular for women. Indeed, the main cause for higher overall costs incurred by women is attributable to the higher likelihood for elderly women to reside in care homes. Interestingly, care home contribution to the overall costs was noticeably lower for patients with multiple comorbidities than for those with none or one comorbidity. This may suggest that sicker patients are more likely to be in hospital than in a care home.

To date, only one single study published in 2004 has estimated the cost of AF in Scotland; the authors estimated the cost of AF in 1995/1996 with the medicalised approach , and projected these to the year 2000 [28]. Previous work has focussed on a 12-months follow-up, which seems limited in order to capture all healthcare resource utilisation for AF patients. Our study offers a longer follow-up and a contemporary estimate of healthcare costs related to AF including all relevant care settings. Our study offers a distinct advantage over previous work as costs, rather than being based on extrapolated rates using a prevalence-based approach [28], are estimated with an incidence-based method using patient-level morbidity records. Using an incidence based approach to costing and a broad perspective to capture the majority of costs associated with AF, several routinely collected administrative datasets from Scotland were combined, including care home utilisation.

Existing studies, including ours, regardless of econometric model choice and covariates used, show that costs due to inpatient admission are the main contributor to overall AF related healthcare cost. This is a pertinent finding that may well support future policies on

For peer review only

opportunistic screening in the population at risk of AF, and in particular in Scotland where 1 in 3 patients with AF are currently undiagnosed [29].

The European AF management guidelines and the Scottish Cross-Party Group ‘Heart Disease and Stroke’, recently recommended that people who are 65 years or older and at risk of AF and associated comorbidities such as cardiovascular disease, diabetes or respiratory disease should be screened opportunistically in primary care, pharmacies or community settings [29, 30]. With rigorous screening and appropriate treatment, hospitalisations could be avoided and costs reduced.

Although we have captured most healthcare sectors and related costs, we were not able to obtain national data on primary care consultations, as these data are currently not routinely available for linkage in Scotland. Not capturing these data, may lead to an underestimation of the size of the AF cohort and associated costs. However, the costs associated with primary care consultations is expected to have a limited impact on the overall total AF related costs. Such underestimation could also result from AF going undiagnosed and clinical miscoding of morbidity records. Nevertheless, by using a cohort of patients hospitalised with AF we were able to capture more severe cases of AF. Prescribing and care home data were only available respectively from 2009 to 2012, their contribution to overall AF related costs might also be underestimated. Other limitations are inherent to the nature of administrative data, such as missing records or incomplete data.

Further, we acknowledge the issue concerning attributing AF related costs to patients with a structural heart disease, as AF may manifest subsequently because of this. In our analysis, we identified about 14% of AF patients with a structural heart disease; these were patients with

For peer review only

systolic dysfunction, valvular heart disease or heart valve replacement. However, from the hospital data it was not possible to establish causation between structural heart disease and AF.

In addition, this is likely to have a marginal impact on our conclusions, as the global comprehensive approach used in this study include expenditures that are not necessarily related to AF.

We also acknowledge that specifying whether patients had received cardiovascular procedures (e.g. cardioversion, echocardiograms and angiograms) would improve the accuracy of our cost estimation, as it would indicate whether costs should be attributable to AF or other forms of structural heart disease. However, this information is not currently available in our routinely collected data of hospital admissions.

Recognising these limitations, we were nevertheless able to harness high quality patient-level linked data to identify a cohort of AF patients and to estimate their associated healthcare utilisation and costs in Scotland.

The inclusion of all available cost components is crucial for establishing overall costs, as these often extend beyond hospitalisation. The study identifies hospitalisation as the main cost driver and suggests that the implementation of AF screening policies could substantially reduce AF related health care costs. Most importantly, the study concludes that patient's age has a limited impact on the overall AF related cost, and therefore may contribute much less to future growth of AF related cost in an ever-ageing Scottish population.

For peer review only

Future work will be able to utilise Scottish Stroke Care Audit (SSCA) records, allowing for the identification of additional AF patients; these are patients hospitalised with a stroke, where AF has been recorded in audit data as an underlying comorbidity.

Being able to complement inpatient records with SSCA records will allow us to capture more AF patients in Scotland. Moreover, future research may be able to include indirect costs associated with productivity-loss by linking morbidity and prescribing data to national data from the Department for Work and Pensions, for instance.

For peer review only

Acknowledgements: We thank the members of the Information Services Division, National Services Scotland, for their support. We acknowledge the support from The Farr Institute @ Scotland. The Farr Institute @ Scotland is supported by a 10-funder consortium: Arthritis Research UK, the British Heart Foundation, Cancer Research UK, the Economic and Social Research Council, the Engineering and Physical Sciences Research Council, the Medical Research Council, the National Institute of Health Research, the National Institute for Social Care and Health Research (Welsh Assembly Government), the Chief Scientist Office (Scottish Government Health Directorates), the Wellcome Trust (MRC Grant No: MR/K007017/1).

Author contributions: GC, CG, PL and OW conceived the article. GC carried out the statistical analysis and prepared the first draft of the manuscript. GC, CG, PL and OW contributed to editing the manuscript and approved the final version submitted for publication.

Funding sources: This work was supported by Farr Institute for Health Informatics Research, Scotland, MRC, grant number MR/K007017/1

Competing interest: The authors declare no conflict of interest.

Data Statement: All data underlying the analyses are confidential and subject to disclosure control. Data can only be obtained through application to Information Services Division (ISD) via the Public Benefit and Privacy Panel (PBPP).

For peer review only

References

1. Heath Improvement Scotland. *Prevention of stroke in patients with atrial fibrillation*. 2013 [cited 2016 January 2016]; Available from: http://www.sign.ac.uk/assets/af_publication.pdf.
2. Bennell, M.C., et al., *Identifying predictors of cumulative healthcare costs in incident atrial fibrillation: a population-based study.* J Am Heart Assoc, 2015. **4**(4).
3. Hallinen, T., et al., *Direct costs of warfarin treatment among patients with atrial fibrillation in a Finnish health care setting.* Curr Med Res Opin, 2006. **22**(4): p. 683-92.
4. Nelson, W.W., et al., *Out-of-range international normalized ratio values and healthcare cost among new warfarin patients with non-valvular atrial fibrillation.* J Med Econ, 2015. **18**(5): p. 333-40.
5. Ghate, S.R., et al., *All-cause and bleeding-related health care costs in warfarin-treated patients with atrial fibrillation.* J Manag Care Pharm, 2011. **17**(9): p. 672-84.
6. Rohrbacker, N.J., et al., *The burden of atrial fibrillation and other cardiac arrhythmias in an employed population: associated costs, absences, and objective productivity loss.* J Occup Environ Med, 2010. **52**(4): p. 383-91.
7. Wu, E.Q., et al., *Economic burden and co-morbidities of atrial fibrillation in a privately insured population.* Curr Med Res Opin, 2005. **21**(10): p. 1693-9.
8. Holstenson, E., et al., *Predictors of costs related to cardiovascular disease among patients with atrial fibrillation in five European countries.* Europace, 2011. **13**(1): p. 23-30.
9. Reynolds, M.R., et al., *Healthcare resource utilization and costs associated with recurrent episodes of atrial fibrillation: the FRACTAL registry.* J Cardiovasc Electrophysiol, 2007. **18**(6): p. 628-33.
10. Jonsson, L., et al., *Cost of illness and drivers of cost in atrial fibrillation in Sweden and Germany.* Appl Health Econ Health Policy, 2010. **8**(5): p. 317-25.
11. Johnsen, S.P., et al., *Cost of illness of atrial fibrillation: a nationwide study of societal impact.* BMC Health Services Research, 2017. **17**(1): p. 714.
12. Tarricone, R., *Cost-of-illness analysis. What room in health economics?* Health Policy, 2006. **77**(1): p. 51-63.
13. de Lagasnerie, G., et al., *The economic burden of diabetes to French national health insurance: a new cost-of-illness method based on a combined medicalized and incremental approach.* Eur J Health Econ, 2018. **19**(2): p. 189-201.
14. Information Services Division Scotland. *General Acute Inpatient and Day Case - Scottish Morbidity Record (SMR01)*. 2015 [cited 2017 November 2017]; Available from: http://www.ndc.scot.nhs.uk/National-Datasets/data.asp?SubID=2.
15. Information Services Division Scotland. *Outpatient Attendance (SMR00)*. 2015 [cited 2015 January 2015]; Available from: http://www.ndc.scot.nhs.uk/National-Datasets/data.asp?SubID=2.
16. Information Service Division Scotland. *Care Home Census 2015*. 2015 [cited 2015 November 2015]; Available from: http://www.isdscotland.org/Health-Topics/Health-and-Social-Community-Care/Care-Homes/Previous-Publications/.
17. Information Service Division Scotland. *Scottish National Tariff*. 2015 [cited 2015 December 2015]; Available from: http://www.isdscotland.org/Health-Topics/Finance/Scottish-National-Tariff/.

18. Healthcare Resource Groups. *Healthcare Resource Groups*. 2015 [cited 2015 November 2015]; Available from: http://www.hscic.gov.uk/hrg.
19. Alvarez-Madrazo, S., et al., *Data Resource Profile: The Scottish National Prescribing Information System (PIS).* International Journal of Epidemiology, 2016.
20. Henry, A.G., et al., *Economic Evaluation in Clinical Trials*. 'Oxford University Press': Oxford, UK.
21. Manning, W.G., A. Basu, and J. Mullahy, *Generalized modeling approaches to risk adjustment of skewed outcomes data.* Journal of Health Economics, 2005. **24**(3): p. 465-488.
22. The Scottish Government. *Scottish Index of Multiple Deprivation*. 2015 [cited 2015 November 2015]; Available from: http://www.gov.scot/Topics/Statistics/SIMD.
23. The Scottish Government. *NHS Boards*. 2015 [cited 2015 January 2015]; Available from: http://www.gov.scot/Topics/Health/NHS-Workforce/NHS-Boards.
24. The Scottish Government. *Scottish Government Urban Rural Classification*. 2015 [cited 2015 January 2015]; Available from: http://www.gov.scot/Topics/Statistics/About/Methodology/UrbanRuralClassification.
25. Charlson, M.E., et al., *A new method of classifying prognostic comorbidity in longitudinal studies: Development and validation.* Journal of Chronic Diseases, 1987. **40**(5): p. 373-383.
26. Leyland, A.H., et al., *Cause-specific inequalities in mortality in Scotland: two decades of change. A population-based study.* BMC Public Health, 2007. **7**: p. 172.
27. Zweifel, P., S. Felder, and M. Meiers, *Ageing of population and health care expenditure: a red herring?* Health Econ, 1999. **8**(6): p. 485-96.
28. Stewart, S., et al., *Cost of an emerging epidemic: an economic analysis of atrial fibrillation in the UK.* Heart, 2004. **90**(3): p. 286-292.
29. Stroke, C.-P.G.o.H.D.a. *A Focus on Atrial Fibrillation in Scotland 2017*. 2017 March 2018]; Available from: https://www.chss.org.uk/documents/2018/01/a-focus-on-atrial-fibrillation-in-scotland-pdf.pdf.
30. Camm, A.J., et al., *2012 focused update of the ESC Guidelines for the management of atrial fibrillation: an update of the 2010 ESC Guidelines for the management of atrial fibrillation. Developed with the special contribution of the European Heart Rhythm Association.* Eur Heart J, 2012. **33**(21): p. 2719-47.

For peer review only

Figure legends

Figure 1. Average annual costs per patient hospitalised with AF by sector. Cost components with confidence interval are presented for each age group.

Figure 2. Average cost per patient hospitalised with AF by Charlson Comorbidity Index. Cost components with confidence interval are presented for each Comorbidity category.

For peer review only

Tables

Table 1. Baseline characteristics of AF patients

Characteristics	N (%)
Number of patients	278,286
Mean age at first admission*(SD)**(range)	74 *(12.5) **(0 -108)
Sex	
Male	139,928 (50.3)
Female	138,358 (49.7)
Health Boards	
Greater Glasgow & Clyde	61,822 (22.2)
Lothian	41,169 (14.8)
Lanarkshire	31,049 (11.2)
Grampian	25,728 (9.3)
Ayrshire & Arran	22,003 (7.9)
Tayside	25,003 (9.0)
Fife	17,954 (6.5)
Highland	18,929 (6.9)
Forth valley	13,664 (4.9)
Dumfries & Galloway	9,798 (3.5)
Borders	7,222 (2.6)
Western isles	1,868 (0.7)
Shetland	1,036 (0.4)
Orkney	1,041 (0.4)
Geography	
Large/urban	106,868 (38.4)
Other/urban	82,601 (29.7)
Accessible small towns	24,938 (9.0)
Remote small towns	8,272 (3.0)
Very remote small towns	3,828 (1.4)
Accessible rural	30,826 (11.1)
Remote rural	10,371 (3.7)
Very remote rural	10,087 (3.6)
SIMD quintile	
1	62,730 (22.5)
2	62,632 (22.5)
3	55,943 (20.1)
4	50,691 (18.2)
5	46,279 (16.6)
Comorbidity	
no comorbidity	40,502 (14.6)
1 comorbidity	53,651 (19.3)
>1 comorbidities	184,133 (66.2)
Re-hospitalised (any condition)	179,494 (64.5)
Admitted to care-home	7,235 (2.6)
Mortality	
Alive	204,690 (73.6)
Dead	73,596 (26.4)

Table 2. Regression results: probability of healthcare resources utilisation and cost estimation

Covariates	Probability (1st modelling part)		Cost Ratios (2nd modelling part)	
	Coefficient (95% CI)	Std. Err	Coefficient (95% CI)	Std. Err
Age group (years)				
0-49	Reference			
50-54	0.329 (0.260, 0.398)	0.035	0.036 (-0.016, 0.087)	0.026
55-59	0.388 (0.326, 0.450)	0.031	0.081 (0.036, 0.127)	0.023
60-64	0.464 (0.407, 0.521)	0.029	0.124 (0.082, 0.166)	0.021
65-69	0.486 (0.432, 0.540)	0.028	0.157 (0.116, 0.198)	0.021
70-74	0.479 (0.426, 0.533)	0.027	0.213 (0.174, 0.252)	0.020
75-79	0.536 (0.482, 0.590)	0.027	0.222 (0.183, 0.260)	0.020
80-84	0.431 (0.375, 0.486)	0.028	0.286 (0.246, 0.326)	0.020
85-89	0.378 (0.318, 0.437)	0.030	0.375 (0.332, 0.417)	0.021
90-max	0.150 (0.083, 0.217)	0.034	0.516 (0.468, 0.564)	0.025
Sex				
Male	Reference			
Female	0.045 (0.028, 0.062)	0.009	0.054 (0.044, 0.064)	0.005
Date of admission	0.169 (0.167, 0.171)	0.001	-0.024 (-0.025, -0.023)	0.001
SIMD quintile				
1	Reference			
2	0.027 (-0.018, 0.071)	0.023	-0.055 (-0.080, -0.031)	0.012
3	-0.041 (-0.086, 0.003)	0.023	-0.080 (-0.106, -0.054)	0.013
4	-0.046 (-0.091, -0.002)	0.023	-0.116 (-0.141, -0.090)	0.013
5	-0.072 (-0.117, -0.027)	0.023	-0.147 (-0.172, -0.122)	0.013
Geography				
Large urban	Reference			
Other urban	-0.130 (-0.156, -0.105)	0.013	-0.023 (-0.037, -0.009)	0.007
Accessible small towns	-0.153 (-0.187, -0.119)	0.017	-0.041 (-0.060, -0.022)	0.010
Accessible rural	-0.197 (-0.230, -0.165)	0.016	-0.043 (-0.062, -0.024)	0.010
Remote small towns	-0.145 (-0.197, -0.093)	0.027	0.009 (-0.023, 0.041)	0.016
Remote rural	-0.288 (-0.335, -0.241)	0.024	-0.036 (-0.065, -0.007)	0.015
Very remote small towns	-0.380 (-0.459, -0.300)	0.041	-0.057 (-0.107, -0.006)	0.026
Very remote rural	-0.346 (-0.407, -0.284)	0.031	-0.061 (-0.102, -0.020)	0.021
Health boards				
Great Glasgow and Clyde	Reference			
Lothian	-0.044 (-0.075, -0.014)	0.016	-0.033 (-0.049, -0.017)	0.008
Lanarkshire	-0.005 (-0.038, 0.029)	0.017	-0.063 (-0.081, -0.045)	0.009
Ayrshire and Arran	-0.358 (-0.394, -0.321)	0.019	-0.046 (-0.068, -0.024)	0.011
Grampian	0.017 (-0.019, 0.054)	0.019	-0.059 (-0.078, -0.039)	0.010
Tayside	-0.402 (-0.436, -0.368)	0.018	-0.083 (-0.103, -0.062)	0.010
Fife	-0.059 (-0.101, -0.017)	0.022	-0.009 (-0.033, 0.016)	0.012
Highland	-0.175 (-0.225, -0.124)	0.026	-0.046 (-0.077, -0.015)	0.016
Forth Valley	-0.477 (-0.518, -0.436)	0.021	-0.109 (-0.135, -0.082)	0.013
Dumfries and Galloway	-0.303 (-0.352, -0.253)	0.025	-0.134 (-0.164, -0.104)	0.015
Borders	-0.501 (-0.554, -0.449)	0.027	-0.086 (-0.120, -0.052)	0.017
Western Isles	-1.072 (-1.171, -0.974)	0.050	0.457 (0.381, 0.533)	0.039

Orkney	-0.362 (-0.492, -0.232)	0.066	-0.029 (-0.117, 0.059)	0.045
Shetland	-0.495 (-0.622, -0.368)	0.065	-0.076 (-0.171, 0.018)	0.048
Mortality within 5 years				
Alive	Reference			
Dead	0.418 (0.376, 0.461)	0.022	0.652 (0.630, 0.674)	0.011
Comorbidity				
no comorbidities	Reference			
1 comorbidity	0.666 (0.567, 0.766)	0.051	0.374 (0.299, 0.450)	0.038
>1 comorbidities	1.205 (1.021, 1.390)	0.094	0.990 (0.910, 1.070)	0.041

Table 3. Regression results: probability of healthcare resources utilisation and cost estimation (alive at the end of the five-year follow-up period)

Covariates	Probability (1st modelling part)		Cost Ratios (2nd modelling part)	
	Coefficient (95% CI)	Std. Err	Coefficient (95% CI)	Std. Err
Age group (years)				
0-49	Reference			
50-54	0.352 (0.282, 0.422)	0.036	0.067 (0.013, 0.120)	0.027
55-59	0.424 (0.361, 0.488)	0.032	0.148 (0.098, 0.199)	0.026
60-64	0.528 (0.470, 0.586)	0.030	0.218 (0.174, 0.263)	0.023
65-69	0.571 (0.516, 0.627)	0.028	0.292 (0.248, 0.336)	0.022
70-74	0.603 (0.549, 0.658)	0.028	0.412 (0.371, 0.454)	0.021
75-79	0.684 (0.630, 0.739)	0.028	0.484 (0.443, 0.525)	0.021
80-84	0.572 (0.516, 0.628)	0.028	0.615 (0.572, 0.659)	0.022
85-89	0.496 (0.435, 0.557)	0.031	0.805 (0.756, 0.854)	0.025
90-max	0.206 (0.134, 0.279)	0.037	1.044 (0.981, 1.106)	0.032
Sex				
Male	Reference			
Female	0.067 (0.048, 0.086)	0.010	0.050 (0.037, 0.063)	0.007
Date of admission	0.171 (0.170, 0.173)	0.001	-0.059 (-0.060, -0.057)	0.001
SIMD quintile				
1	Reference			
2	0.021 (-0.009, 0.050)	0.015	-0.052 (-0.071, -0.033)	0.010
3	-0.023 (-0.054, 0.008)	0.016	-0.081 (-0.101, -0.060)	0.011
4	-0.045 (-0.077, -0.014)	0.016	-0.117 (-0.138, -0.096)	0.011
5	-0.051 (-0.083, -0.020)	0.016	-0.160 (-0.181, -0.139)	0.011
Geography				
Large urban	Reference			
Other urban	-0.140 (-0.169, -0.112)	0.014	-0.030 (-0.049, -0.012)	0.010
Accessible small towns	-0.172 (-0.210, -0.134)	0.019	-0.052 (-0.077, -0.026)	0.013
Accessible rural	-0.217 (-0.253, -0.181)	0.018	-0.061 (-0.086, -0.037)	0.013
Remote small towns	-0.145 (-0.203, -0.087)	0.030	-0.007 (-0.048, 0.035)	0.021
Remote rural	-0.319 (-0.371, -0.268)	0.026	-0.064 (-0.101, -0.027)	0.019
Very remote small towns	-0.404 (-0.491, -0.318)	0.044	-0.098 (-0.161, -0.036)	0.032
Very remote rural	-0.360 (-0.428, -0.293)	0.034	-0.087 (-0.138, -0.035)	0.026

Health boards				
Great Glasgow and Clyde	Reference			
Lothian	-0.055 (-0.090, -0.020)	0.018	-0.051 (-0.072, -0.030)	0.011
Lanarkshire	0.003 (-0.034, 0.040)	0.019	-0.072 (-0.095, -0.048)	0.012
Ayrshire and Arran	-0.396 (-0.436, -0.355)	0.021	-0.064 (-0.093, -0.035)	0.015
Grampian	0.029 (-0.013, 0.070)	0.021	-0.051 (-0.077, -0.026)	0.013
Tayside	-0.453 (-0.491, -0.415)	0.019	-0.094 (-0.120, -0.067)	0.014
Fife	-0.087 (-0.134, -0.040)	0.024	-0.024 (-0.057, 0.008)	0.017
Highland	-0.191 (-0.247, -0.135)	0.029	-0.037 (-0.075, 0.001)	0.020
Forth Valley	-0.520 (-0.566, -0.474)	0.023	-0.108 (-0.141, -0.074)	0.017
Dumfries and Galloway	-0.314 (-0.369, -0.259)	0.028	-0.166 (-0.206, -0.127)	0.020
Borders	-0.547 (-0.605, -0.489)	0.030	-0.099 (-0.144, -0.054)	0.023
Western Isles	-1.164 (-1.264, -1.063)	0.051	0.139 (0.057, 0.221)	0.042
Orkney	-0.394 (-0.535, -0.252)	0.072	0.002 (-0.114, 0.117)	0.059
Shetland	-0.605 (-0.740, -0.470)	0.069	-0.044 (-0.172, 0.085)	0.066
Comorbidity				
no comorbidities	Reference			
1 comorbidity	0.705 (0.602, 0.808)	0.052	0.432 (0.352, 0.513)	0.041
>1 comorbidities	1.165 (0.974, 1.357)	0.098	1.133 (1.041, 1.226)	0.047

Table 4. Regression results: probability of healthcare resources utilisation and cost estimation (dead at the end of the five-year follow-up period)

Covariates	Probability (1st modelling part)		Cost Ratios (2nd modelling part)	
	Coefficient (95% CI)	Std. Err	Coefficient (95% CI)	Std. Err
Age group (years)				
0-49	Reference			
50-54	0.150 (-0.125, 0.426)	0.141	-0.112 (-0.405, 0.180)	0.149
55-59	0.134 (-0.098, 0.366)	0.118	-0.093 (-0.334, 0.147)	0.123
60-64	0.129 (-0.080, 0.338)	0.107	0.000 (-0.208, 0.209)	0.106
65-69	0.129 (-0.067, 0.326)	0.101	-0.011 (-0.212, 0.189)	0.102
70-74	0.107 (-0.084, 0.298)	0.097	0.016 (-0.180, 0.213)	0.100
75-79	0.128 (-0.059, 0.315)	0.095	-0.005 (-0.198, 0.189)	0.099
80-84	0.132 (-0.053, 0.318)	0.095	0.056 (-0.136, 0.247)	0.098
85-89	-0.048 (-0.233, 0.137)	0.094	0.066 (-0.126, 0.257)	0.098
90-max	-0.518 (-0.702, -0.333)	0.094	0.097 (-0.095, 0.290)	0.098
Sex				
Male	Reference			
Female	0.048 (0.033, 0.063)	0.008	0.028 (0.014, 0.043)	0.007
Date of admission	-0.040 (-0.042, -0.039)	0.001	0.004 (0.002, 0.005)	0.001
SIMD quintile				
1	Reference			
2	0.033 (0.011, 0.055)	0.011	0.015 (-0.005, 0.036)	0.011
3	0.058 (0.034, 0.082)	0.012	-0.008 (-0.030, 0.015)	0.012
4	0.065 (0.039, 0.090)	0.013	-0.017 (-0.041, 0.007)	0.012

For peer review only - http://bmjopen.bmj.com/site/about/guidelines.xhtml

5	0.113 (0.088, 0.138)	0.013	-0.024 (-0.049, 0.000)	0.012
Geography				
Large urban	Reference			
Other urban	-0.010 (-0.032, 0.012)	0.011	-0.033 (-0.054, -0.012)	0.011
Accessible small towns	-0.006 (-0.036, 0.025)	0.015	-0.049 (-0.077, -0.021)	0.014
Accessible rural	-0.031 (-0.060, -0.001)	0.015	-0.036 (-0.064, -0.008)	0.014
Remote small towns	-0.054 (-0.102, -0.005)	0.025	0.003 (-0.042, 0.049)	0.023
Remote rural	-0.038 (-0.084, 0.009)	0.024	-0.012 (-0.057, 0.034)	0.023
Very remote small towns	-0.065 (-0.147, 0.017)	0.042	0.036 (-0.052, 0.123)	0.045
Very remote rural	0.014 (-0.051, 0.078)	0.033	-0.002 (-0.068, 0.065)	0.034
Health boards				
Great Glasgow and Clyde	Reference			
Lothian	0.029 (0.004, 0.055)	0.013	0.029 (0.006, 0.053)	0.012
Lanarkshire	-0.052 (-0.080, -0.023)	0.014	-0.034 (-0.061, -0.008)	0.013
Ayrshire and Arran	-0.122 (-0.155, -0.089)	0.017	0.011 (-0.020, 0.042)	0.016
Grampian	0.075 (0.044, 0.106)	0.016	-0.057 (-0.086, -0.028)	0.015
Tayside	-0.024 (-0.056, 0.007)	0.016	-0.061 (-0.089, -0.033)	0.014
Fife	-0.028 (-0.064, 0.008)	0.018	0.047 (0.012, 0.082)	0.018
Highland	0.034 (-0.015, 0.084)	0.025	-0.065 (-0.117, -0.013)	0.027
Forth Valley	-0.060 (-0.099, -0.021)	0.020	-0.123 (-0.161, -0.085)	0.019
Dumfries and Galloway	-0.027 (-0.074, 0.020)	0.024	-0.014 (-0.058, 0.029)	0.022
Borders	-0.058 (-0.112, -0.005)	0.027	-0.023 (-0.074, 0.029)	0.026
Western Isles	-0.033 (-1.168, 1.102)	0.579	0.305 (-0.165, 0.775)	0.240
Orkney	0.191 (0.055, 0.327)	0.069	-0.180 (-0.317, -0.042)	0.070
Shetland	-0.031 (-0.170, 0.108)	0.071	-0.187 (-0.323, -0.052)	0.069
Comorbidity				
no comorbidities	Reference			
1 comorbidity	-0.176 (-0.449, 0.097)	0.139	0.147 (-0.127, 0.422)	0.140
>1 comorbidities	-0.256 (-0.491, -0.021)	0.120	0.626 (0.401, 0.851)	0.115

Table 5. Average annual costs per patient hospitalised with AF by sex

Sex	Cost estimates	
	Mean total cost (%)	95% CI
Male		
Inpatient	2935 (79.99)	(2915, 2955)
Outpatient	31 (8.46)	(308, 313)
Care home	165 (4.50)	(154, 177)
PIS	242 (6.60)	(240, 245)
Total	3669	(3872, 3927)
Female		
Inpatient	3022 (77.49)	(3001, 3042)
Outpatient	310 (7.96)	(308, 313)
Care home	268 (6.88)	(255, 281)
PIS	259 (6.64)	(256, 262)
Total	3968	(3872, 3927)

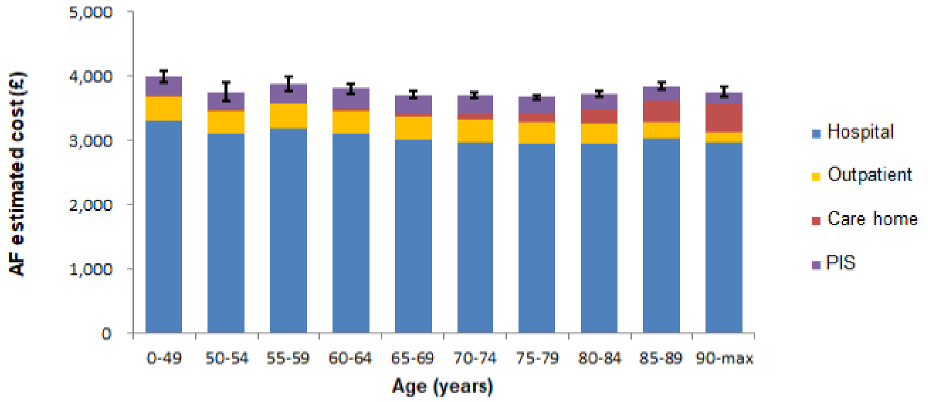

Age groups	Inpatient total cost (£) Mean (95%)	Outpatient total cost (£) Mean (95%)	Care home total cost (£) Mean (95%)	PIS total cost (£) Mean (95%)
(0-49)	3306 (3146, 3466)	382 (366, 399)	19 (12, 51)	285 (259, 311)
(50-54)	3107 (2979, 3236)	367 (354, 379)	19 (9, 47)	270 (253, 287)
(55-59)	3193 (3100, 3287)	370 (361, 379)	22 (2, 43)	290 (278, 302)
(60-64)	3107 (3044, 3170)	364 (357, 371)	47 (28, 67)	293 (285, 302)
(65-69)	3009 (2960, 3058)	363 (358, 368)	53 (37, 69)	286 (280, 291)
(70-74)	2969 (2931, 3007)	355 (351, 359)	95 (78, 111)	282 (278, 287)
(75-79)	2954 (2923, 2985)	340 (336, 343)	118 (104, 132)	267 (264, 271)
(80-84)	2962 (2930, 2994)	299 (296, 303)	225 (207, 242)	247 (244, 250)
(85-89)	3038 (3002, 3074)	249 (245, 253)	340 (318, 363)	224 (221, 228)
(90-max)	2971 (2926, 3017)	161 (157, 165)	435 (404, 466)	191 (186, 195)
Total	2978 (2964, 2993)	310 (309, 312)	226 (217, 235)	250 (248, 252)

Average annual costs per patient hospitalised with AF by sector. Cost components with confidence interval are presented for each age group.

59x47mm (300 x 300 DPI)

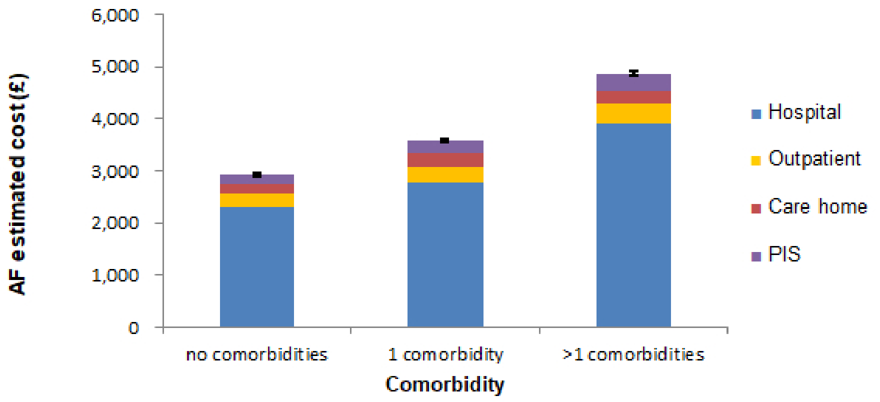

Component	No comorbidity cost (£) Mean (95%)	1 comorbidity cost (£) Mean (95%)	>1 comorbidity cost (£) Mean (95%)
Inpatient	2295 (2274, 2316)	2787 (2761, 2812)	3890 (3857, 3922)
Outpatient	276 (274, 279)	294 (291, 297)	388 (385, 392)
Care home	183 (167, 199)	249 (231, 266)	235 (221, 248)
PIS	175 (173, 177)	247 (244, 250)	348 (343, 353)
Total	2927 (2899, 2955)	3590 (3556, 3624)	4909 (4868, 4949)

Average cost per patient hospitalised with AF by Charlson Comorbidity Index. Cost components with confidence interval are presented for each Comorbidity category.

54x37mm (300 x 300 DPI)

ONLINE SUPPLEMENT

Equation I. Probability of healthcare utilisation

$$\Pr(HCE_{it} > 0) = \alpha + \beta_1 \sum_{s=2}^{10} A_{it} + \beta_2 G_i + \beta_3 Y_i + \beta_4 \sum_{s=2}^{5} S_i + \beta_5 \sum_{u=2}^{8} U_i + \beta_6 \sum_{h=2}^{14} H_i$$

$$+ \beta_7 \sum_{c=2}^{3} C_{it} + \beta_8 D_i + \left(\beta_9 \sum_{s=2}^{5} S_i * D_i\right) + \left(\beta_{10} \sum_{c=2}^{3} C_{it} * \sum_{s=2}^{10} A_{it}\right) + u_i$$

Where: A is age at the time of admission (reference category: 0-49 age group); G is sex (reference category: male); Y year of admission; S is SIMD quintile (reference category: most deprived quintile (1)); U is the urban/rural classification (reference category: large urban area);H is health board of inpatient admission (reference category: Greater Glasgow & Clyde); C is the Charlson comorbidity index (reference category: no comorbidities); D is mortality during five year follow-up; u_i is the error term for patient *i* at time *t*.

Equation II. Cost estimation

$$E[HCE] = g(x\beta)$$

Where xβ is the linear predictor for HCE

Equation III. Multiplying first and second part

$$E[HCE|X] = \Pr(HCE > 0|X) * E[HCE|HCE > 0, X]$$

For peer review only
For peer review only - http://bmjopen.bmj.com/site/about/guidelines.xhtml

Table I. Regression interactions: probability of healthcare resources utilisation and cost estimation

Covariates	Probability (1st modelling part) Coefficient (95%CI)	Std. Err	Probability (2nd modelling part) Coefficient (95%CI)	Std. Err
**Interaction: SIMD - Mortality within 5 years**				
1	Reference			
2	-0.015 (-0.069, 0.038)	0.027	0.056 (0.027, 0.086)	0.015
3	0.046 (-0.007, 0.100)	0.027	0.067 (0.036, 0.097)	0.016
4	0.017 (-0.037, 0.071)	0.028	0.089 (0.059, 0.120)	0.016
5	0.069 (0.013, 0.125)	0.029	0.100 (0.069, 0.132)	0.016
**Interaction: age (year) - Charlson score (1 comorbidity)**				
0-49	Reference			
50-54	-0.195 (-0.342, -0.049)	0.075	-0.016 (-0.124, 0.092)	0.055
55-59	-0.384 (-0.514, -0.255)	0.066	-0.081 (-0.173, 0.012)	0.047
60-64	-0.459 (-0.579, -0.340)	0.061	-0.116 (-0.202, -0.031)	0.044
65-69	-0.500 (-0.613, -0.386)	0.058	-0.161 (-0.244, -0.078)	0.042
70-74	-0.510 (-0.621, -0.399)	0.057	-0.202 (-0.283, -0.121)	0.041
75-79	-0.570 (-0.680, -0.461)	0.056	-0.197 (-0.276, -0.117)	0.041
80-84	-0.594 (-0.704, -0.484)	0.056	-0.209 (-0.290, -0.128)	0.041
85-89	-0.643 (-0.756, -0.531)	0.058	-0.215 (-0.298, -0.132)	0.042
90-max	-0.709 (-0.828, -0.590)	0.061	-0.267 (-0.357, -0.178)	0.045
**Interaction: age (year) - Charlson score (>1 comorbidities)**				
0-49	Reference			
50-54	-0.449 (-0.685, -0.213)	0.121	-0.214 (-0.316, -0.111)	0.052
55-59	-0.539 (-0.751, -0.327)	0.108	-0.209 (-0.305, -0.112)	0.049
60-64	-0.534 (-0.734, -0.334)	0.102	-0.323 (-0.412, -0.234)	0.045
65-69	-0.573 (-0.767, -0.378)	0.099	-0.436 (-0.523, -0.350)	0.044
70-74	-0.650 (-0.842, -0.459)	0.098	-0.520 (-0.604, -0.436)	0.043
75-79	-0.767 (-0.957, -0.577)	0.097	-0.556 (-0.639, -0.473)	0.042
80-84	-0.857 (-1.047, -0.667)	0.097	-0.625 (-0.709, -0.541)	0.043
85-89	-0.967 (-1.159, -0.775)	0.098	-0.661 (-0.747, -0.576)	0.044
90-max	-1.074 (-1.270, -0.878)	0.100	-0.805 (-0.896, -0.714)	0.046

For peer review only - http://bmjopen.bmj.com/site/about/guidelines.xhtml

Equation IV. Probability of healthcare utilisation (alive at the end of the five-year follow-up period)

$$\Pr(HCE_{it} > 0) = \alpha + \beta_1 \sum_{s=2}^{10} A_{it} + \beta_2 G_i + \beta_3 Y_i + \beta_4 \sum_{s=2}^{5} S_i + \beta_5 \sum_{u=2}^{8} U_i + \beta_6 \sum_{h=2}^{14} H_i$$

$$+ \beta_7 \sum_{c=2}^{3} C_{it} + \left(\beta_8 \sum_{c=2}^{3} C_{it} * \sum_{s=2}^{10} A_{it} \right) + u_i$$

Where: A is age at the time of admission (reference category: 0 -49 age group); G is sex (reference category: male); Y year of admission; S is SIMD quintile (reference category: most deprived quintile (1)); U is the urban/rural classification (reference category: large urban area);H is health board of inpatient admission (reference category: Greater Glasgow & Clyde); C is the Charlson comorbidity index (reference category: no comorbidities); u_i is the error term for patient *i* at time *t*.

For peer review only

For peer review only - http://bmjopen.bmj.com/site/about/guidelines.xhtml

Table II. Regression interactions: probability of healthcare resources utilisation and cost estimation (alive at the end of the five-year follow-up period)

Covariates	Probability (1st modelling part) Coefficient (95%CI)	Std. Err	Probability (2nd modelling part) Coefficient (95%CI)	Std. Err
**Interaction: age (year) - Charlson score (1 comorbidity)**				
0-49	Reference			
50-54	-0.190 (-0.341, -0.039)	0.077	-0.015 (-0.126, 0.097)	0.057
55-59	-0.400 (-0.534, -0.266)	0.069	-0.087 (-0.187, 0.014)	0.051
60-64	-0.484 (-0.608, -0.360)	0.063	-0.116 (-0.208, -0.023)	0.047
65-69	-0.531 (-0.649, -0.412)	0.060	-0.143 (-0.233, -0.053)	0.046
70-74	-0.559 (-0.674, -0.443)	0.059	-0.195 (-0.283, -0.107)	0.045
75-79	-0.635 (-0.750, -0.521)	0.058	-0.207 (-0.293, -0.121)	0.044
80-84	-0.680 (-0.796, -0.565)	0.059	-0.215 (-0.304, -0.125)	0.046
85-89	-0.730 (-0.850, -0.610)	0.061	-0.254 (-0.349, -0.159)	0.048
90-max	-0.827 (-0.959, -0.695)	0.067	-0.281 (-0.393, -0.169)	0.057
**Interaction: age (year) - Charlson score (>1 comorbidities)**				
0-49	Reference			
50-54	-0.408 (-0.658, -0.158)	0.127	-0.226 (-0.342, -0.109)	0.059
55-59	-0.504 (-0.726, -0.281)	0.114	-0.235 (-0.348, -0.122)	0.058
60-64	-0.525 (-0.735, -0.316)	0.107	-0.339 (-0.443, -0.236)	0.053
65-69	-0.580 (-0.784, -0.377)	0.104	-0.448 (-0.549, -0.347)	0.051
70-74	-0.713 (-0.913, -0.513)	0.102	-0.565 (-0.663, -0.467)	0.050
75-79	-0.820 (-1.019, -0.621)	0.101	-0.648 (-0.745, -0.552)	0.049
80-84	-0.938 (-1.137, -0.739)	0.102	-0.702 (-0.801, -0.603)	0.051
85-89	-1.074 (-1.276, -0.872)	0.103	-0.769 (-0.873, -0.665)	0.053
90-max	-1.196 (-1.406, -0.986)	0.107	-0.932 (-1.051, -0.814)	0.060

For peer review only - http://bmjopen.bmj.com/site/about/guidelines.xhtml

Figure I. Average annual costs per patient hospitalised with AF by sector. Cost components with confidence interval are presented for each age group (alive at the end of the five-year follow-up period)

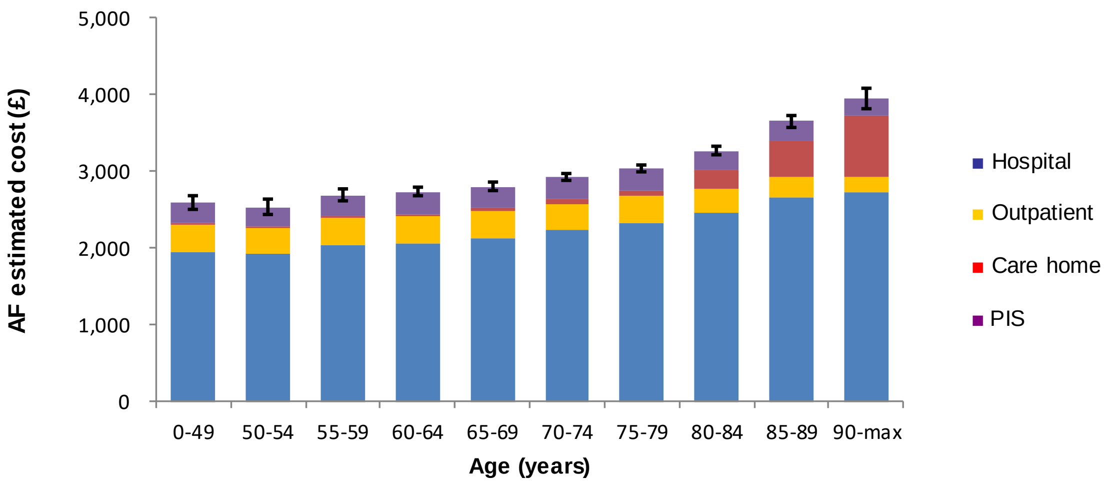

Age groups	Inpatient total cost (£) Mean (95%)	Outpatient total cost (£) Mean (95%)	Care home total cost (£) Mean (95%)	PIS total cost (£) Mean (95%)
(0-49)	1956 (1846, 2066)	360 (344, 375)	17 (16, 49)	267 (243, 291)
(50-54)	1916 (1826, 2007)	344 (332, 356)	14 (8, 37)	259 (242, 275)
(55-59)	2041 (1973, 2110)	351 (342, 360)	17 (1,37)	280 (269, 292)
(60-64)	2071 (2022, 2119)	351 (344, 358)	26 (13, 38)	290 (281, 299)
(65-69)	2135 (2093, 2178)	352 (347, 357)	32 (21, 44)	285 (279, 291)
(70-74)	2230 (2195, 2266)	352 (347, 356)	63 (49, 76)	286 (281, 291)
(75-79)	2329 (2297, 2361)	346 (342, 350)	84 (71, 97)	277 (273, 281)
(80-84)	2457 (2420, 2494)	314 (310, 318)	236 (212, 260)	266 (262, 269)
(85-89)	2651 (2603, 2698)	272 (267, 278)	480 (439, 522)	252 (248, 257)
(90-max)	2734 (2663, 2806)	188 (181, 194)	811 (735, 887)	225 (219, 231)
Total	2253 (2238, 2267)	321 (319, 323)	199 (188, 209)	264 (262, 266)

For peer review only - http://bmjopen.bmj.com/site/about/guidelines.xhtml

Equation V. Probability of healthcare utilisation (dead at the end of the five-year follow-up period)

$$\Pr(HCE_{it} > 0) = \alpha + \beta_1 \sum_{s=2}^{10} A_{it} + \beta_2 G_i + \beta_3 Y_i + \beta_4 \sum_{s=2}^{5} S_i + \beta_5 \sum_{u=2}^{8} U_i + \beta_6 \sum_{h=2}^{14} H_i$$

$$+ \beta_7 \sum_{c=2}^{3} C_{it} + \left(\beta_8 \sum_{c=2}^{3} C_{it} * \sum_{s=2}^{10} A_{it} \right)$$

Where: A is age at the time of admission (reference category: 0 -49 age group); G is sex (reference category: male); Y year of admission; S is SIMD quintile (reference category: most deprived quintile (1)); U is the urban/rural classification (reference category: large urban area);H is health board of inpatient admission (reference category: Greater Glasgow & Clyde); C is the Charlson comorbidity index (reference category: no comorbidities); u_i is the error term for patient *i* at time *t*.

Note: the model for care home does not include the 0 -49 and 50-54 age groups, as none of those patients incurred any cost related to care home.

For peer review only - http://bmjopen.bmj.com/site/about/guidelines.xhtml

Table III. Regression interactions: probability of healthcare resources utilisation and cost estimation (dead at the end of the five-year follow-up period)

Covariates	Probability (1st modelling part)		Probability (2nd modelling part)	
	Coefficient (95%CI)	Std. Err	Coefficient (95%CI)	Std. Err
**Interaction: age (year) - Charlson score (1 comorbidity)**				
0-49	Reference			
50-54	-0.057 (-0.456, 0.343)	0.204	0.124 (-0.268, 0.515)	0.200
55-59	0.130 (-0.207, 0.468)	0.172	0.054 (-0.282, 0.389)	0.171
60-64	0.083 (-0.223, 0.389)	0.156	-0.009 (-0.309, 0.291)	0.153
65-69	0.028 (-0.263, 0.320)	0.149	-0.054 (-0.342, 0.234)	0.147
70-74	-0.006 (-0.290, 0.278)	0.145	-0.051 (-0.334, 0.232)	0.144
75-79	-0.078 (-0.358, 0.201)	0.143	0.002 (-0.278, 0.282)	0.143
80-84	-0.200 (-0.478, 0.078)	0.142	-0.048 (-0.326, 0.230)	0.142
85-89	-0.160 (-0.437, 0.117)	0.141	-0.039 (-0.316, 0.239)	0.142
90-max	-0.154 (-0.431, 0.123)	0.141	-0.079 (-0.358, 0.200)	0.142
**Interaction: age (year) - Charlson score (>1 comorbidities)**				
0-49	Reference			
50-54	0.000 (-0.340, 0.339)	0.173	0.066 (-0.268, 0.400)	0.170
55-59	-0.147 (-0.436, 0.142)	0.147	0.041 (-0.240, 0.322)	0.143
60-64	-0.135 (-0.398, 0.128)	0.134	-0.114 (-0.360, 0.132)	0.126
65-69	-0.215 (-0.466, 0.036)	0.128	-0.164 (-0.401, 0.074)	0.121
70-74	-0.192 (-0.436, 0.053)	0.125	-0.237 (-0.470, -0.005)	0.119
75-79	-0.311 (-0.553, -0.070)	0.123	-0.248 (-0.478, -0.018)	0.117
80-84	-0.443 (-0.683, -0.204)	0.122	-0.367 (-0.595, -0.139)	0.116
85-89	-0.394 (-0.633, -0.154)	0.122	-0.394 (-0.623, -0.166)	0.116
90-max	-0.312 (-0.551, -0.073)	0.122	-0.483 (-0.713, -0.254)	0.117

For peer review only - http://bmjopen.bmj.com/site/about/guidelines.xhtml

Figure II. Average annual costs per patient hospitalised with AF by sector. Cost components with confidence interval are presented for each age group (dead at the end of the five-year follow-up period)

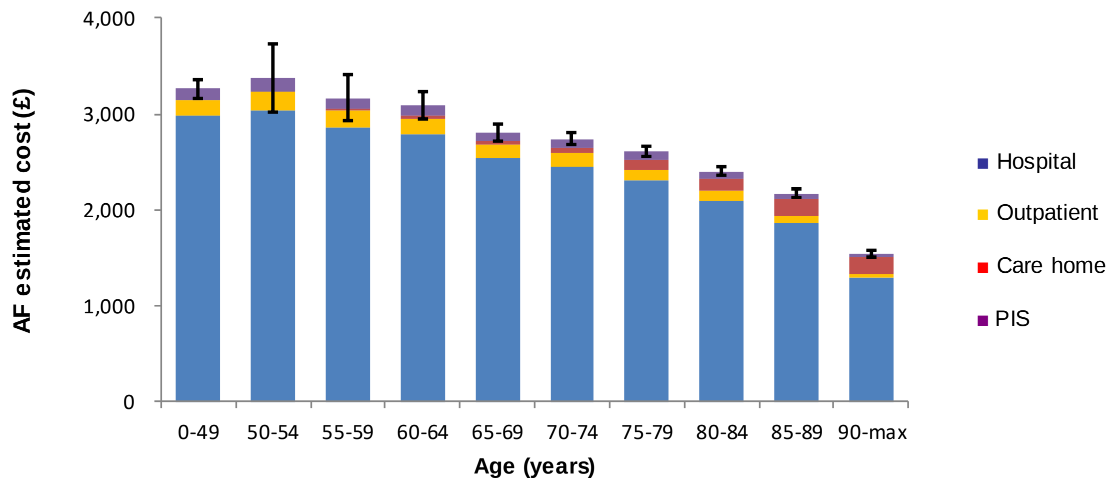

Age groups	Inpatient total cost (£) Mean (95%)	Outpatient total cost (£) Mean (95%)	Care home total cost (£) Mean (95%)	PIS total cost (£) Mean (95%)
(0-49)	2976 (2603, 3349)	161 (136, 186)	n/a	125 (65, 185)
(50-54)	3037 (2712, 3362)	203 (177, 228)	n/a	130 (97, 164)
(55-59)	2865 (2649, 3080)	181 (165, 197)	9 (5, 23)	115 (95, 135)
(60-64)	2795 (2661, 2929)	162 (153, 170)	28 (3, 53)	111 (99, 122)
(65-69)	2536 (2450, 2621)	154 (147, 160)	26 (12, 39)	95 (88, 102)
(70-74)	2448 (2386, 2510)	138 (134, 142)	60 (42, 78)	97 (91, 102)
(75-79)	2302 (2257, 2347)	118 (115, 121)	100 (80, 120)	83 (80, 86)
(80-84)	2099 (2062, 2136)	93 (91, 95)	139 (121, 157)	70 (67, 72)
(85-89)	1859 (1826, 1892)	69 (67, 71)	187 (167, 206)	58 (56, 60)
(90-max)	1289 (1263, 1316)	33 (31, 34)	180 (162, 199)	35 (34, 36)
Total	2018 (2001, 2034)	91 (90, 93)	142 (132, 152)	66 (65, 67)

Note: the care home total cost estimation does not include the 0 -49 and 50-54 age groups, as none of those patients incurred any cost related to care home.

For peer review only - http://bmjopen.bmj.com/site/about/guidelines.xhtml

STROBE 2007 (v4) Statement—Checklist of items that should be included in reports of *cohort studies*

Section/Topic	Item #	Recommendation	Reported on page #
**Title and abstract**	1	(*a*) Indicate the study's design with a commonly used term in the title or the abstract	1
		(*b*) Provide in the abstract an informative and balanced summary of what was done and what was found	1
**Introduction**			
Background/rationale	2	Explain the scientific background and rationale for the investigation being reported	3
Objectives	3	State specific objectives, including any prespecified hypotheses	3
**Methods**			
Study design	4	Present key elements of study design early in the paper	3-4
Setting	5	Describe the setting, locations, and relevant dates, including periods of recruitment, exposure, follow-up, and data collection	4-5
Participants	6	(*a*) Give the eligibility criteria, and the sources and methods of selection of participants. Describe methods of follow-up	4-5
		(*b*) For matched studies, give matching criteria and number of exposed and unexposed	n/a
Variables	7	Clearly define all outcomes, exposures, predictors, potential confounders, and effect modifiers. Give diagnostic criteria, if applicable	7-8
Data sources/ measurement	8*	For each variable of interest, give sources of data and details of methods of assessment (measurement). Describe comparability of assessment methods if there is more than one group	n/a
Bias	9	Describe any efforts to address potential sources of bias	7
Study size	10	Explain how the study size was arrived at	4
Quantitative variables	11	Explain how quantitative variables were handled in the analyses. If applicable, describe which groupings were chosen and why	7-8
Statistical methods	12	(*a*) Describe all statistical methods, including those used to control for confounding	6-8
		(*b*) Describe any methods used to examine subgroups and interactions	6-7
		(*c*) Explain how missing data were addressed	n/a
		(*d*) If applicable, explain how loss to follow-up was addressed	n/a
		(*e*) Describe any sensitivity analyses	n/a
**Results**			

For peer review only

Participants	13*	(a) Report numbers of individuals at each stage of study—eg numbers potentially eligible, examined for eligibility, confirmed eligible, included in the study, completing follow-up, and analysed	n/a
		(b) Give reasons for non-participation at each stage	n/a
		(c) Consider use of a flow diagram	n/a
Descriptive data	14*	(a) Give characteristics of study participants (eg demographic, clinical, social) and information on exposures and potential confounders	n/a
		(b) Indicate number of participants with missing data for each variable of interest	n/a
		(c) Summarise follow-up time (eg, average and total amount)	n/a
Outcome data	15*	Report numbers of outcome events or summary measures over time	n/a
Main results	16	(*a*) Give unadjusted estimates and, if applicable, confounder-adjusted estimates and their precision (eg, 95% confidence interval). Make clear which confounders were adjusted for and why they were included	9-11
		(*b*) Report category boundaries when continuous variables were categorized	n/a
		(*c*) If relevant, consider translating estimates of relative risk into absolute risk for a meaningful time period	n/a
Other analyses	17	Report other analyses done—eg analyses of subgroups and interactions, and sensitivity analyses	10
**Discussion**			
Key results	18	Summarise key results with reference to study objectives	11-12
**Limitations**			
Interpretation	20	Give a cautious overall interpretation of results considering objectives, limitations, multiplicity of analyses, results from similar studies, and other relevant evidence	12-14
Generalisability	21	Discuss the generalisability (external validity) of the study results	14
**Other information**			
Funding	22	Give the source of funding and the role of the funders for the present study and, if applicable, for the original study on which the present article is based	17

*Give information separately for cases and controls in case-control studies and, if applicable, for exposed and unexposed groups in cohort and cross-sectional studies.

Note: An Explanation and Elaboration article discusses each checklist item and gives methodological background and published examples of transparent reporting. The STROBE checklist is best used in conjunction with this article (freely available on the Web sites of PLoS Medicine at http://www.plosmedicine.org/, Annals of Internal Medicine at http://www.annals.org/, and Epidemiology at http://www.epidem.com/). Information on the STROBE Initiative is available at www.strobe-statement.org.

For peer review only
